# Orphan nuclear receptors recruit TRIM28 to promote telomeric H3K9me3 for the ALT pathway

**DOI:** 10.1038/s44318-026-00760-w

**Published:** 2026-03-31

**Authors:** Chia-Tsen Tsai, Venus Marie Gaela, Hsuan-Yu Hsia, Yu-Chen Huang, Yi-Ling Shen, Liuh-Yow Chen

**Affiliations:** 1 Molecular and Cell Biology, Taiwan International Graduate Program, Academia Sinica and Graduate Institute of Life Sciences, National Defense Medical Center, Taipei, Taiwan; 2 Institute of Molecular Biology, Academia Sinica, Taipei, Taiwan; 3 Institute of Molecular and Cellular Biology, National Taiwan University, Taipei, Taiwan

**Keywords:** Alternative Lengthening of Telomeres, TRIM28, Orphan Nuclear Receptors, H3K9me3, Chromatin, Transcription & Genomics, DNA Replication, Recombination & Repair

## Abstract

Alternative lengthening of telomeres (ALT) is a telomere maintenance mechanism deployed in embryonic stem cells and cancer cells. High levels of the heterochromatin mark H3 lysine 9 trimethylation (H3K9me3) at telomeres are critical for ALT, but how this is achieved remains unclear. Telomeric association of orphan nuclear receptors (NRs)—such as COUP-TF1, COUP-TF2, TR2, and TR4—has been shown previously to facilitate ALT activation. Here, we show that orphan NRs regulate telomeric H3K9me3 through TRIM28, a corepressor of ZNF transcription factors, to promote ALT. We report that H3K9me3 is induced by telomeric association of orphan NRs in cultured human fibroblast and ALT cancer cell lines. Moreover, TRIM28 is required for the orphan-NR-induced H3K9 methylation and ALT phenotypes. Importantly, physical interaction of TRIM28 with orphan NRs induces telomeric localization of TRIM28. A TRIM28 variant defective in orphan-NR interaction fails to localize to telomeres and is unable to promote H3K9me3 and ALT phenotypes. These findings indicate that telomeric orphan NRs recruit TRIM28 for telomeric H3K9me3 and ALT activation, emphasizing the role of chromatin structure in ALT activation.

## Synopsis

Heterochromatin mark H3K9me3 at telomeres plays a critical role in activation of alternative lengthening of telomeres. This study shows that telomeric orphan nuclear receptors (NRs) recruit TRIM28 to promote telomeric H3K9me3 and ALT activity, revealing the regulation of telomere heterochromatin in ALT cells.

- In ALT cells, telomeric H3K9me3 is maintained by orphan NRs and TRIM28.
- Telomeric tethering of orphan NRs or the KRAB transcriptional repressor domain in human fibroblasts induces H3K9me3 and ALT phenotypes via TRIM28.
- Orphan NRs associate with ALT telomeres and interact with TRIM28 for H3K9me3 maintenance and ALT activation in cancer cell lines.

## Abstract (web summary)

The KRAB-ZFP transcriptional corepressor TRIM28 provides a link between the role of nuclear receptors and heterochromatin in alternative lengthening of telomeres.

## Graphical abstract

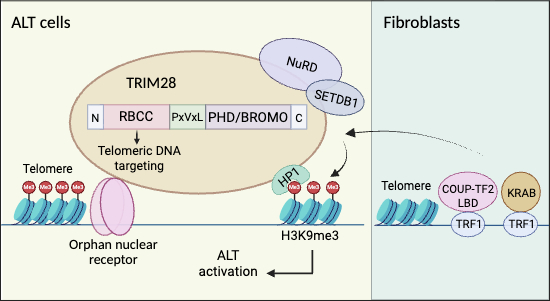

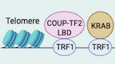

## Introduction

Telomeres, composed of repetitive DNA sequences at chromosome ends, are crucial for genomic stability by preventing their recognition as double-strand breaks. This protective mechanism is mediated by the telomere-associated shelterin protein complex, which safeguards telomeres from initiating the DNA damage response (Moyzis et al, 1988; Palm and De Lange, 2008). In somatic cells, cell division results in telomere shortening due to the end-replication problem, ultimately leading to cellular senescence (Harley et al, 1990; Wright and Shay, 1992). Cancer cells overcome this limitation by adopting telomere maintenance mechanisms, either by reactivating telomerase or activating the alternative lengthening of telomeres (ALT) pathway. ALT enacts homologous recombination (HR), reminiscent of break-induced replication, to lengthen telomeres and enable indefinite proliferation (Bryan et al, 1997; Dilley et al, 2016; Zhang et al, 2019). It is characterized by several molecular hallmarks. For instance, in ALT cells, telomeres are encapsulated in promyelocytic leukemia nuclear bodies (PML-NBs), forming ALT-associated PML-NBs (APBs) (Yeager et al, 1999). APBs are hubs where telomere DNA and DNA damage response factors cluster, together catalyzing ALT recombination and break-induced replication (Cho et al, 2014; Zhang et al, 2021). In addition, telomeric DNA in ALT cells exhibits spontaneous damage due to replication stress, resulting in telomere excision and accumulation of extrachromosomal telomeric repeats (ECTRs) (Cesare and Griffith, 2004; Cesare et al, 2009; Chen et al, 2017; Wang et al, 2004). Moreover, levels of telomeric repeat-containing RNAs (TERRAs), which can form RNA:DNA hybrids that are sources of replication stress, are elevated in ALT cells (Chu et al, 2017; Feretzaki et al, 2020). Thus, these ALT phenotypes reflect ALT activation and influence oncogenesis.

Regulation of telomere chromatin plays a critical role in ALT activation. Zinc finger protein 827 (ZNF827) recruits the nucleosome remodeling and histone deacetylation (NuRD) complex to ALT telomeres, leading to histone deacetylation (Conomos et al, 2014). In turn, histone deacetylation depletes shelterin from telomeres and promotes telomere interaction and compaction. Additionally, the ZNF827-NuRD complex recruits HR-associated proteins to telomeres and, through its chromatin remodeling function, promotes ALT activity (Conomos et al, 2014). Involvement of the ZNF827-NuRD complex in ALT telomere maintenance may be related to the role of the NuRD complex in heterochromatin assembly during DNA replication (Sims and Wade, 2011).

Previous studies have shown that ALT telomeric chromatin is less compact yet highly heterochromatic compared to telomerase-positive telomeres. The heterochromatin mark histone H3 lysine 9 trimethylation (H3K9me3) is enriched at ALT telomeres (Cubiles et al, 2018). Telomeres in mouse embryonic stem cells (mESCs) exhibit heterochromatic features, ALT-like phenotypes, and increased recombination. A proteomic study of telomere chromatin from mESCs revealed the telomeric association of the SET Domain Bifurcated 1 (SETDB1) histone methyltransferase, which is required for telomeric H3K9me3 and also regulates APB formation in mESCs (Gauchier et al, 2019). SETDB1 is also associated with ALT telomeres in cancer cells, where its depletion reduces telomeric H3K9me3 and ALT phenotypes, supporting the functional significance of heterochromatin to ALT activation (Gauchier et al, 2019). Interestingly, SETDB1 also regulates TERRA transcription, indicating that telomeric heterochromatin is compatible with transcriptional elongation at telomeres (Gauchier et al, 2019). The telomeric localization and protein stability of SETDB1 in ALT cells both depend on Tripartite motif-containing 28 (TRIM28), a corepressor of the family of Krüppel-associated box zinc finger proteins (KRAB-ZFP) (Friedman et al, 1996; Moosmann et al, 1996; Schultz et al, 2001). Consistent with its SETDB1 interaction, TRIM28 promotes occupancy of H3K9me3 on telomeres in ALT cells (Wang et al, 2021). However, depletion of TRIM28 results in telomere lengthening and inconsistent ALT phenotypes. This paradox underscores the need for further research to clarify the role of heterochromatin and TRIM28 in ALT telomere maintenance.

Moreover, ALT activation is associated with a deficiency in the histone H3.3 chaperone complex α-thalassemia/mental retardation syndrome X-linked (ATRX) and death-domain-associated protein (DAXX). Restoration of ATRX expression in ATRX-negative ALT cell lines has been shown to suppress many ALT-associated phenotypes (Clynes et al, 2015; Napier et al, 2015). However, knockdown of ATRX or DAXX in either mortal or telomerase-positive cell lines results in reduced histone deposition and telomeric H3K9me3, telomere decompaction, and replication dysfunction, though loss of either protein is not sufficient to immediately activate ALT (Li et al, 2019; Lovejoy et al, 2012; O’Sullivan et al, 2014). Intriguingly, Gauchier et al demonstrated that ATRX depletion induces ALT recombination in mESCs that is dependent on H3K9me3 (Gauchier et al, 2019), indicating that ALT activation may require the combined effects of ATRX deficiency and increased levels of H3K9me3 at telomeres. However, the mechanism driving formation of telomeric H3K9me3 in ALT cells remains to be determined.

Orphan nuclear receptors (NRs) of the NR2C/F class—such as COUP-TF1, COUP-TF2, TR2, TR4, and EAR2—associate with telomeres in ALT cells by binding to TCAGGG telomere variant repeats and regulate ALT activity (Conomos et al, 2012; Dejardin and Kingston, 2009). These orphan NRs facilitate recruitment of the ZNF827-NuRD complex to telomeres, which remodels telomeric chromatin and enhances recombination (Conomos et al, 2014). Additionally, orphan NRs bind directly to FANCD2, a crucial component of the Fanconi anemia repair pathway, to initiate a DNA damage response that boosts ALT activity in ALT cells (Xu et al, 2019). Recently, we developed a system to ectopically express orphan NRs, tethering them to telomeres in non-ALT fibroblasts to explore the molecular mechanisms by which orphan NRs regulate ALT (Gaela et al, 2024). Our previous findings indicate that the telomeric association of orphan NRs is sufficient to trigger APB formation and induce various ALT phenotypes and telomere recombination. This ALT induction is mediated by activation function 2 (AF2) domains of orphan NRs and ZNF827, indicating an involvement of altered chromatin structures in orphan NR-induced ALT activation. These findings highlight the critical role of orphan NRs in ALT regulation (Gaela et al, 2024). Building on this system, in the current study, we aimed to investigate how orphan NRs contribute to ALT chromatin regulation through TRIM28, with a particular focus on elucidating the mechanisms by which H3K9me3-marked heterochromatin is established at telomeres for ALT activation.

## Results

### Orphan NRs promote telomeric H3K9me3

**Figure 1:**
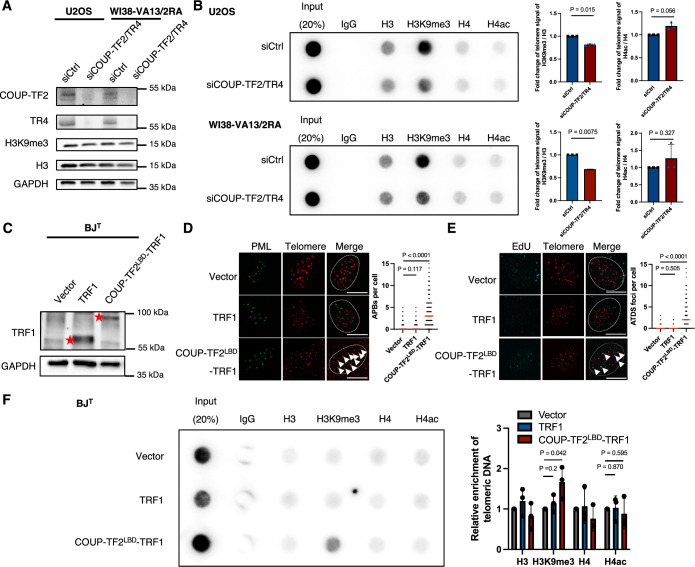
Role of orphan nuclear receptors (NRs) in H3K9me3 activation at telomeres. (**A**) Western blot analysis showing the expression levels of COUP-TF2, TR4, H3, H3K9me3 and GAPDH in U2OS and WI38-VA13/2RA cells following treatment for 6 days with specific siRNAs targeting these NRs. (**B**) Telomere-ChIP analysis of U2OS and WI38-VA13/2RA cells 6 days after COUP-TF2 and TR4 knockdown to assess enrichment for telomeric DNA with the indicated histones and histone modifications. Data are normalized to the corresponding parental histone signal. (me, methylation; ac, acetylation). (**C**) Western blot analysis of BJ^T^ cells ectopically expressing TRF1 alone or COUP-TF2^LBD^-TRF1, confirming successful expression of the constructs with an anti-TRF1 antibody. Red stars indicate ectopic expression of TRF1 or COUP-TF2^LBD^-TRF1. (**D**) Representative images showing PML and telomere co-localization in BJ^T^ cells expressing COUP-TF2^LBD^-TRF1. IF detected PML, and telomeres were detected by FISH using the TelC PNA probe. Co-localization of PML (green) and telomeres (red) appears yellow. White outlines indicate DAPI segmentation. White arrows indicate APBs. Dot plots quantifying the numbers of APBs or telomeres in individual BJ^T^ cells (*n* > 150). (**E**) Representative images showing EdU incorporation at telomeres in BJ^T^ cells expressing vector, TRF1 alone or COUP-TF2^LBD^-TRF1. IF detects EdU, and telomeres are visualized by FISH using the TelC PNA probe. Co-localization of EdU (cyan) and telomeres (red) appears white. White outlines indicate DAPI segmentation. White arrows indicate co-localized EdU and telomere foci (ATDS). A dot plot quantifies the co-localization of telomeres with EdU in BJ^T^ cells (*n* > 150). (**F**) Telomere-ChIP analysis on BJ^T^ cells expressing TRF1 alone or COUP-TF2^LBD^-TRF1, showing enrichment for telomeric DNA with the indicated histones and histone marks. All bar graphs represent quantitation of the telomeric DNA pulled down, normalized to control cells (mean ± SD; *n* = 3 independent biological replicates). Statistical significance is noted as follows: ns*P* > 0.05, *P < 0.05, **P < 0.01, as determined by the unpaired *t* test. (**D**, **E**) Statistical significance is denoted as follows: ns*P* > 0.05, *P < 0.05, **P < 0.01, ***P < 0.001, ****P < 0.0001), as determined by Mann–Whitney *U* test. Source data are available online for this figure.

**Figure EV1:**
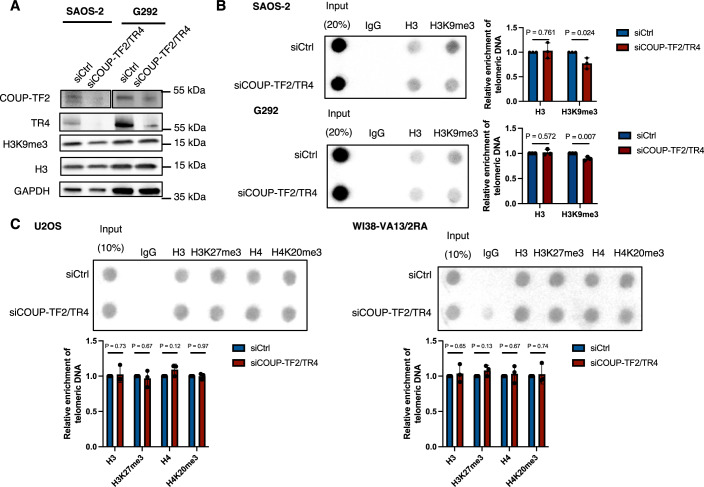
Role of orphan nuclear receptors (NRs) in H3K9me3 activation at telomeres and characterization of COUP-TF2^LBD^-TRF1 in promoting APB formation and ATDS. (**A**) Western blot analysis showing the expression levels of COUP-TF2, TR4, H3, H3K9me3 and GAPDH in SAOS-2 and G292 cells following treatment for 6 days with specific siRNAs targeting these NRs. (**B**) Telomere-ChIP analysis of SAOS-2 and G292 cells 6 days after COUP-TF2 and TR4 knockdown to assess enrichment for telomeric DNA with the indicated histones and histone modifications (mean ± SD; *n* = 3 independent biological replicates). (**C**) Telomere-ChIP analysis of U2OS and WI38-VA13/2RA cells 6 days after COUP-TF2 and TR4 knockdown to assess enrichment for telomeric DNA with the indicated histones and histone modifications (mean ± SD; *n* = 3 independent biological replicates). (**B**, **C**) Statistical significance is noted as follows: ns*P* > 0.05, *P < 0.05, as determined by the unpaired *t* test. Source data are available online for this figure.

The telomeric association of orphan NRs has been shown previously to promote ALT (Conomos et al, 2014; Conomos et al, 2012; Gaela et al, 2024), yet the exact mechanism remains to be established. Structural alterations in telomeric chromatin, including changes in histone modifications such as H3K9me3 and H4 acetylation (H4ac), have been linked to ALT activation (Conomos et al, 2014; Gauchier et al, 2019). To determine how orphan NRs regulate the telomeric epigenetic structures of ALT cells, first, we performed telomere-chromatin immunoprecipitation (telomere-ChIP) to detect histone H3K9me3 and H4ac in cultured ALT cell lines, including U2OS osteosarcoma cells and transformed WI38-VA13/2RA fibroblasts. Our results revealed that both these histone modifications are present on telomeric histones (Fig. 1B). Given that COUP-TF2 and TR4 are highly expressed in U2OS and WI38-VA13/2RA cells and are known to localize to telomeres (Conomos et al, 2012; Dejardin and Kingston, 2009), we silenced their expression by means of small interfering RNAs (siRNAs) (Fig. 1A) to assess their impact on telomeric H3K9me3 and H4ac. We observed that simultaneous knockdown of COUP-TF2 and TR4 from U2OS and WI38-VA13/2RA cells caused a significant decrease in the levels of telomeric H3K9me3, but not in H4ac levels (Fig. 1B), without affecting global H3K9me3 levels (Fig. 1A). The same result of telomere H3K9me3 reduction upon COUP-TF2 and TR4 depletion was observed in two additional ALT osteosarcoma cell lines, SAOS-2 and G292 (Fig. EV1A,B). Moreover, we examined H3K27me3 and H4K20me3 heterochromatin-associated marks on telomeres in WI38-VA13/2RA and U2OS cells and found that these histone modifications were not affected by COUP-TF2 and TR4 knockdown (Fig. EV1C). These results indicate that orphan NRs specifically modulate H3K9me3 at telomeres in ALT cells.

We have previously demonstrated that targeting orphan NRs to telomeres is sufficient to induce ALT activity and various ALT features in primary human fibroblast cells (Gaela et al, 2024). Adopting the same strategy herein, we endeavored to uncover a direct role for orphan NRs in regulating telomere chromatin. Consistently, we found that ectopically stable expression of the COUP-TF2 ligand-binding domain (LBD) fused with TRF1 (COUP-TF2^LBD^-TRF1), but not TRF1 alone, in telomerase-immortalized human BJ fibroblast cells (BJ^T^) led to ALT induction, which was characterized by co-localization of telomeres with PML-NBs, thereby forming APBs, and by the occurrence of non-S phase ALT telomere DNA synthesis (ATDS) (Fig. 1C–E) (Gaela et al, 2024). Furthermore, telomere-ChIP revealed an increase in telomeric H3K9me3 modification, but not of H4ac, in BJ^T^ cells expressing COUP-TF2^LBD^-TRF1 relative to vector (Fig. 1F). This result indicates that telomeric targeting of COUP-TF2 LBD can promote histone H3K9me3 on telomeres. Collectively, these data reveal that the telomere association of orphan NRs in ALT cells promotes structural changes in telomeric chromatin by inducing telomeric H3K9me3 occupancy.

### Alteration of telomere chromatin structures promotes ALT activity and associated features

**Figure 2:**
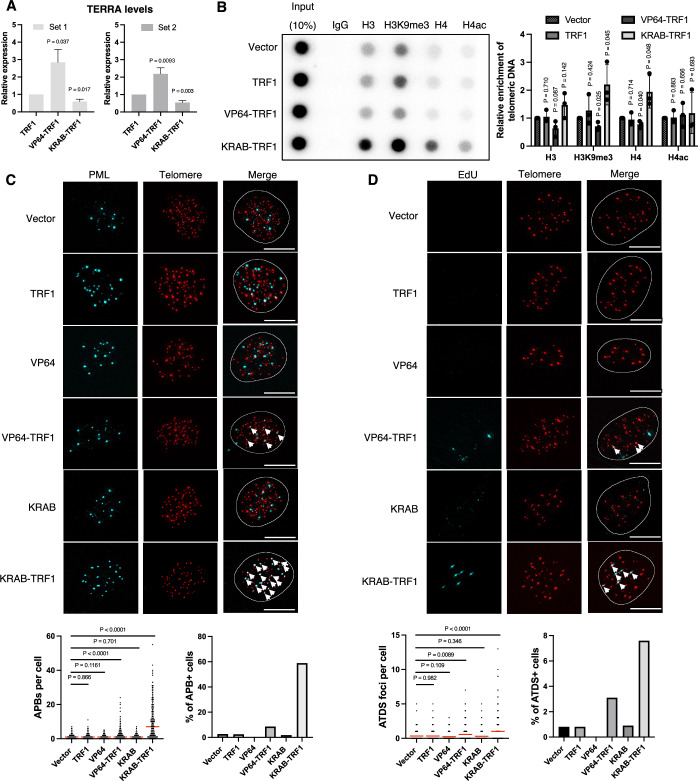
KRAB-TRF1-mediated heterochromatin formation enhances ALT induction. (**A**) Quantification of TERRA expression levels in VP64-TRF1 or KRAB-TRF1 BJ^T^ cells, normalized to those of TRF1 BJ^T^ cells, as determined by qPCR of RNA samples. TERRA transcripts from subtelomeric regions were quantified using primer set 1 targeting Chr10q and primer set 2 targeting Chr15q, with expression levels normalized to GAPDH. (mean ± SEM; *n* = 5 biological independent experiments). (**B**) Telomere-ChIP analysis in BJ^T^ cells expressing TRF1, VP64-TRF1 or KRAB-TRF1 reveals enrichment for telomeric DNA with various histones and histone modifications. Bar graphs represent the quantification of telomeric DNA pulled down, normalized to vector-administered cells (mean ± SD; *n* = 3 independent biological replicates). (**C**) Representative immunofluorescence images depicting the localization of PML and telomeres as APBs in BJ^T^ cells expressing different plasmids. Immunofluorescence (IF) identifies exogenous proteins and PML, whereas telomeres are visualized by FISH using the TelC PNA probe. Co-localization (white arrows) of PML (cyan) and telomeres (red) appears white. White outlines indicate DAPI segmentation. A dot plot quantifies the number of APBs in individual BJ^T^ cells (*n* > 150). A bar chart illustrates the percentages of APB-positive (APB + ) cells, defined as those containing more than five APBs. (**D**) Representative images showing EdU incorporation at telomeres in BJ^T^ cells. IF detects EdU, and telomeres are visualized by FISH using the TelC PNA probe. Co-localization (white arrows) of EdU (cyan) and telomeres (red) appears white. White outlines indicate DAPI segmentation. A dot plot quantifies the co-localization of telomeres with EdU in BJ^T^ cells (*n* > 150). A bar chart displays the percentage of ATDS-positive (ATDS + ) cells containing more than three EdU+ Telomere foci. Red lines indicate the mean. (**C**, **D**) Statistical significance is denoted as follows: ns*P* > 0.05, *P < 0.05), **P < 0.01), ***P < 0.001), ****P < 0.0001, as determined by Mann–Whitney *U* test. (**A**, **B**) Statistical significance is noted as follows: ns*P* > 0.05, *P < 0.05, as determined by the unpaired *t* test. Source data are available online for this figure.

**Figure EV2:**
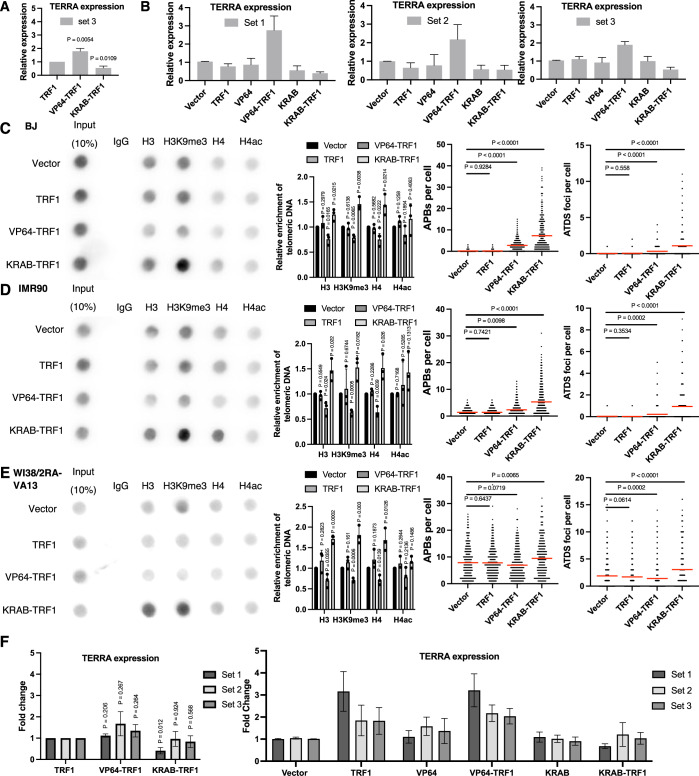
KRAB-TRF1 and VP64-TRF1 modulate telomeric chromatin and ALT-associated phenotypes in fibroblasts and ALT cells. (**A**) Quantification of TERRA expression levels in VP64-TRF1 or KRAB-TRF1 BJ^T^ cells, normalized to those of TRF1 BJ^T^ cells, as determined by qPCR of RNA samples. TERRA transcripts from subtelomeric regions were quantified using primer set 3 targeting 5q, 7q, 9q, 10q, 13q, 16q, 20q, and 22q, with expression levels normalized to GAPDH. (**B**) Quantification of TERRA expression levels in KRAB-TRF1 or VP64-TRF1 BJ^T^ cells normalized to Flag BJ^T^ cells, as determined by qPCR of RNA samples. TERRA transcripts from subtelomeric regions were quantified using primer set 1 targeting Chr10q, primer set 2 targeting Chr15q, and primer set 3 targeting 5q, 7q, 9q, 10q, 13q, 16q, 20q, and 22q, with expression levels normalized to GAPDH. (**C**–**E**) Telomere-ChIP analysis in BJ, IMR90 and WI38-VA13/2RA cells expressing TRF1, KRAB-TRF1, or VP64-TRF1 reveals enrichment for telomeric DNA with various histones and histone modifications. Bar graphs present the quantification of telomeric DNA pulled down, normalized to vector-administered cells (mean ± SD; *n* = 3 independent biological replicates). First dot plot quantifies the number of APBs in individual BJ, IMR90, or WI38-VA13/2RA cells (*n* > 150). Left bar charts illustrate the percentages of APB-positive (APB + ) cells, defined as those containing more than five APBs. Second dot plot quantifies the co-localization of telomeres with EdU in BJ^T^ cells (*n* > 150). Right bar charts display the percentage of ATDS-positive (ATDS + ) cells containing more than three EdU+ Telomere foci. Red lines indicate the mean. (**F**) Quantification of TERRA expression in VP64-TRF1 or KRAB-TRF1 WI38-VA13/2RA cells relative to TRF1 WI38-VA13/2RA cells (left) or Flag WI38-VA13/2RA cells (right). TERRA transcripts were measured using primer set 1 targeting 10q, primer set 2 targeting 15q, and primer set 3 targeting 5q, 7q, 9q, 10q, 13q, 16q, 20q, and 22q. (**A**, **B**, **F**) (mean ± SEM; *n* = 5 biological independent experiments). (**A**, **F**) Statistical significance is noted as follows: ns*P* > 0.05, *P < 0.05, **P < 0.01, as determined by the unpaired *t* test. (**C**–**E**) Statistical significance is denoted as follows: ns*P* > 0.05, *P < 0.05, **P < 0.01, ***P < 0.001, ****P < 0.0001, as determined by Mann–Whitney *U* test. Source data are available online for this figure.

As evidenced above, structural alterations in telomeric chromatin are associated with orphan NR-mediated ALT induction, consistent with a link between distinct telomeric chromatin environments and ALT maintenance (Li et al, 2019; O’Sullivan et al, 2014; Wang et al, 2021). To further investigate the causative role of chromatin states in ALT induction, we ectopically expressed in BJ^T^ cells the transcriptional repressor Krüppel-associated box (KRAB) or the transcriptional activator herpes simplex virus VP64, both of which were fused to TRF1. These stably expressed fusion proteins were used to specifically induce the formation of heterochromatin and euchromatin, respectively, at the telomeres of the BJ^T^ cells. First, we confirmed transcriptional effector activities by assessing expression of TERRAs by means of reverse transcription polymerase chain reaction (RT-PCR) (Feretzaki and Lingner, 2017). As anticipated, we observed that TERRA expression was suppressed by KRAB-TRF1 and upregulated by VP64-TRF1 relative to cells expressing TRF1 alone (Figs. 2A and EV2A,B). Furthermore, we assessed the effect of telomeric targeting of KRAB or VP64 in BJ^T^ cells on chromatin modifications through telomere-ChIP. Our results show that KRAB-TRF1 expression significantly increased telomeric histone occupancies by H3, H4, and H3K9me3, indicative of heterochromatic telomere formation. In contrast, VP64-TRF1 expression reduced telomere histone occupancy and H3K9me3 levels (Fig. 2B). Together, these results demonstrate that KRAB-TRF1 and VP64-TRF1 induce heterochromatin and euchromatin formation, respectively, at telomeres in BJ^T^ cells. Next, we examined ALT induction following these chromatin alterations. Robust APB formation and ATDS were observed in BJ^T^ cells expressing KRAB-TRF1, whereas VP64-TRF1 expression only induced minimal APB formation and ATDS (Fig. 2C,D). Notably, these ALT features were absent from cells expressing empty vector, TRF1 alone, KRAB alone, or VP64 alone. Moreover, similar effects of VP64-TRF1 and KRAB-TRF1 on chromatin changes and ALT induction were also observed in telomerase-negative primary BJ and IMR90 fibroblast cells (Fig. EV2C,D). These results suggest that telomere chromatin changes may drive ALT induction in human fibroblasts.

We also expressed VP64-TRF1 and KRAB-TRF1 in WI38-VA13/2RA ALT cells to analyze chromatin structure and ALT phenotypes (Fig. EV2E). RT-PCR results showed inconsistent effects on TERRA expression, likely due to the already high TERRA expression in the cells (Fig. EV2F). Moreover, VP64-TRF1 reduced histone occupancy and H3K9me3 modification in WI38-VA13/2RA cells, consistent with the observations in human fibroblasts. However, VP64-TRF1 slightly reduced APB and ATDS levels in WI38-VA13/2RA cells, contrary to the results in human fibroblasts. Notably, previous studies have shown that experimentally-induced TERRA expression triggers ALT activity in ALT and non-ALT cells (In et al, 2025; Silva et al, 2022), suggesting that TERRA induction and heterochromatin formation may represent independent pathways for triggering ALT. Thus, VP64-TRF1-induced ALT phenotypes in fibroblasts might be attributed to TERRA induction rather than chromatin alteration per se, whereas VP64-TRF1 reduced H3K9me3 and consequently lowered ALT activity in WI38-VA13/2RA cells. Importantly, KRAB-TRF1 promoted H3K9me3 and ALT activity in WI38-VA13/2RA cells, similar to human fibroblast cells. Altogether, these data underscore the significance of heterochromatin in ALT activation.

### TRIM28 promotes telomeric H3K9me3 and ALT induction

**Figure EV3:**
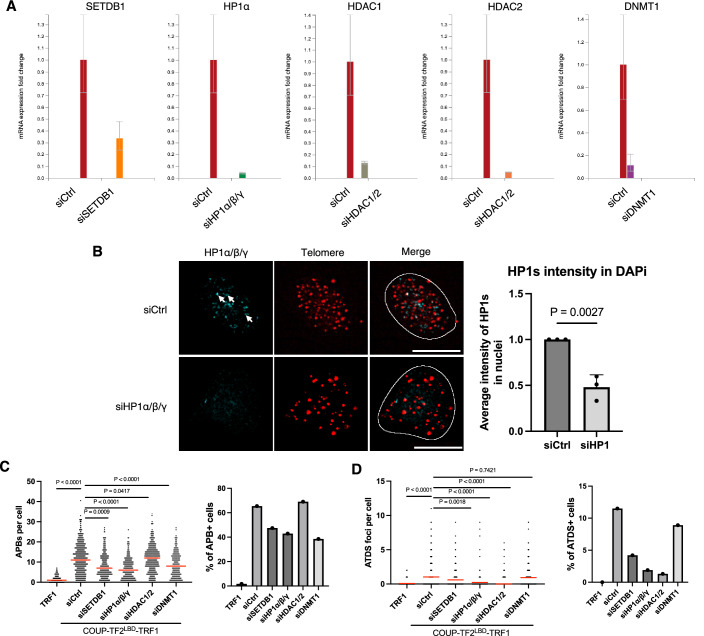
Functional analysis of siRNA-mediated knockdown of heterochromatin-associated proteins in BJ^T^ cells expressing COUP-TF2^LBD^-TRF1. (**A**) qPCR and (**B**) IF data showing the gene silencing efficiency of siRNA sequences targeting specific RNAs in BJ^T^ cells expressing COUP-TF2^LBD^-TRF1. For qPCR, data represent the mean of three technical replicates (mean ± SEM; *n* = 3). For IF, the bar chart indicates the average HP1 signal intensity per nucleus (mean ± SEM; *n* = 3 independent biological replicates). White outlines indicate DAPI segmentation and scale bar, 10 μm. White arrows indicate foci localized at telomeres. (**C**) Dot plots to quantify the number of APBs in TRF1 or COUP-TF2^LBD^-TRF1 BJ^T^ cells treated with specific siRNAs targeting heterochromatin-associated proteins (*n* > 150 cells). (**D**) Dot plots showing quantification of EdU+ APB foci in TRF1 or COUP-TF2^LBD^-TRF1 BJ^T^ cells treated with specific siRNAs targeting heterochromatin-associated proteins (*n* > 150 cells). Red lines in the dot plots indicate the mean. Statistical significance is denoted as follows: ns*P* > 0.05, **P < 0.01, ****P < 0.0001, as determined by Mann–Whitney *U* test. All knockdown experiments involved treating cells with specific siRNAs for 6 days. Source data are available online for this figure.

When associated with telomeres, orphan NRs and KRAB promote H3K9me3 and trigger ALT induction, consistent with the notion that changes in chromatin structure may underlie ALT activation. To further elucidate the significance of chromatin changes for orphan NR-mediated ALT induction, we silenced the expression of KRAB-associated chromatin regulators, such as SET domain bifurcated histone lysine methyltransferase 1 (SETDB1), heterochromatin protein 1 (HP1)α/β/γ, Histone deacetylase (HDAC) 1/2 or DNA (cytosine-5)-methyltransferase 1 (DNMT1) in COUP-TF2^LBD^-TRF1 BJ^T^ cells (Czerwińska et al, 2017) (Fig. EV3A,B). We found that depletion of any of these chromatin regulators reduced APB formation and ATDS (Fig. EV3C,D), supporting that chromatin alterations may contribute to orphan NR-induced ALT activation.

**Figure 3:**
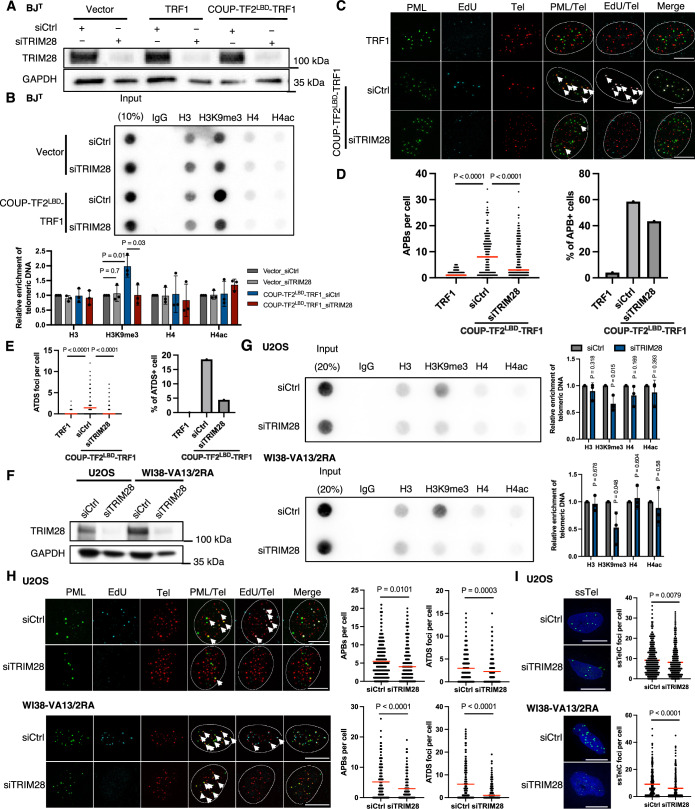
TRIM28 mediates H3K9me3 activation and ALT induction. (**A**) Western blot analysis of TRIM28 expression in BJ^T^ cells after 6 days of treatment with TRIM28-targeting siRNAs. (**B**) Telomere-ChIP analysis of BJ^T^ cells 6 days following TRIM28 knockdown to assess enrichment for telomeric DNA with specific histones and histone modifications. Bar graphs display quantitation of the telomeric DNA pulled down, normalized to control cells (mean ± SD; *n* = 3 independent biological replicates). (**C**) Representative images showing EdU incorporation at telomeres and PML in BJ^T^ cells. EdU and PML are detected by immunofluorescence (IF), and telomeres are visualized by FISH using the TelC PNA probe. Co-localization (white arrows) of EdU (cyan) and telomeres (red) appears white, whereas that of PML (green) and telomeres (red) appears yellow. Scale bar, 10 μm. (**D**) Dot plots quantifying the numbers of APBs or telomeres in individual BJ^T^ cells (*n* > 150). A bar chart displays the percentages of APB-positive (APB + ) cells, defined as those containing more than five APBs. (**E**) Dot plots quantifying the numbers of EdU+ Telomere foci in individual BJ^T^ cells (*n* > 150). A bar chart displays the percentage of EdU+ telomere-positive (ATDS + ) cells containing more than three EdU+ Telomere co-localizing foci. (**F**) Western blot analysis of TRIM28 expression in U2OS and WI38-VA13/2RA cells after 6 days of treatment with TRIM28-targeting siRNAs. (**G**) Telomere-ChIP analysis of U2OS and WI38-VA13/2RA cells 6 days following TRIM28 knockdown to assess enrichment for telomeric DNA with specific histones and histone modifications. Bar graphs show quantitation of the telomeric DNA pulled down, normalized to input and control cells (mean ± SD; *n* = 3 independent biological replicates). (**H**) Representative images showing EdU incorporation at telomeres and PML in U2OS and WI38-VA13/2RA cells. EdU and PML are detected by IF, and telomeres are visualized by FISH using the TelC PNA probe. Co-localization (white arrows) of EdU (cyan) and telomeres (red) appears white, whereas that of PML (green) and telomeres (red) appears yellow. Scale bar, 10 μm. Dot plots show the quantification of APBs or EdU+ Telomere (ATDS) foci in individual U2OS and WI38-VA13/2RA cells (*n* > 150). (**I**) Representative images of single-stranded C-rich telomeric DNA (ssTelC) in U2OS and WI38-VA13/2RA cells. Scale bar, 10 μm. A dot plot quantifies the numbers of ssTelC foci in individual cells. ssTelC was detected by native FISH using the TelG PNA probe. Red lines in the dot plots indicate the median. (**B**, **G**) Statistical significance is noted as follows: ns*P *> 0.05, *P < 0.05, as determined by the unpaired *t* test. (**D**, **E**, **H**, **I**) Statistical significance is indicated as follows: ns*P* > 0.05, *P < 0.05, **P < 0.01, ***P < 0.001, ****P < 0.0001, as determined by Mann–Whitney *U* test. Source data are available online for this figure.

Intriguingly, SETDB1, HP1α/β/γ, HDAC1/2, and DNMT1 have all been shown previously to associate with TRIM28 and are recruited together for gene regulation by KRAB domain-containing zinc-finger transcription factors via TRIM28-KRAB domain interactions (Friedman et al, 1996). Moreover, Wang et al recently reported that TRIM28 localizes to telomeres in ALT cells and impacts H3K9me3 (Wang et al, 2021), suggesting that TRIM28 might be involved in orphan NR-mediated ALT regulation. To examine this possibility, we silenced TRIM28 expression in BJ^T^ cells by means of siRNAs to investigate its effects on COUP-TF2^LBD^-TRF1-mediated telomeric H3K9me3 and ALT induction (Fig. 3A). Telomere-ChIP experiments revealed that TRIM28 depletion had a limited effect on telomeric H3K9me3 in vector-administered BJ^T^ cells, whereas levels of H3K9me3 in the COUP-TF2^LBD^-TRF1-expressing cells were significantly reduced (Fig. 3B). These results indicate that TRIM28 regulates COUP-TF2^LBD^-induced telomeric H3K9me3, but not maintenance of basal telomeric H3K9me3 in BJ^T^ cells (Fig. 3B). Furthermore, knockdown of TRIM28 reduced APB formation and ATDS in BJ^T^ cells expressing COUP-TF2^LBD^-TRF1 (Fig. 3C–E). Together, these findings support that TRIM28 plays a crucial role in COUP-TF2-induced ALT induction by regulating telomere chromatin in human fibroblasts.

**Figure EV4:**
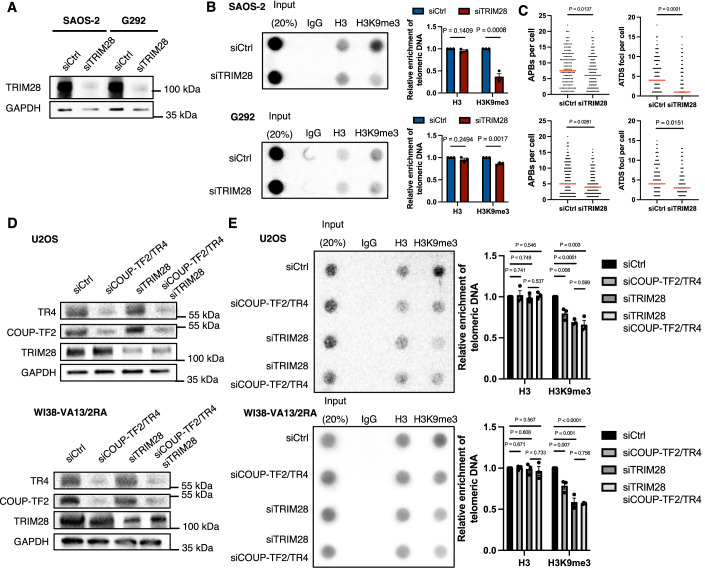
TRIM28 depletion reduces telomeric H3K9me3, APBs, and ATDS in ALT cells and shows an epistatic effect with orphan NRs. (**A**) Western blot analysis of TRIM28 expression in SAOS-2 and G292 cells after 6 days of treatment with TRIM28-targeting siRNAs. (**B**) Telomere-ChIP analysis of U2OS and WI38-VA13/2RA cells 6 days following TRIM28 knockdown to assess enrichment for telomeric DNA with specific histones and histone modifications. Bar graphs show quantitation of the telomeric DNA pulled down, normalized to input and control cells (mean ± SD; *n* = 3 independent biological replicates). (**C**) Dot plots show the quantification of APBs or EdU+ Telomere (ATDS) foci in individual SAOS-2 and G292 cells (*n* > 150). (**D**) Western blot analysis showing the expression levels of COUP-TF2, TR4, TRIM28 and GAPDH in U2OS and WI38-VA13/2RA cells following treatment for 6 days with specific siRNAs targeting these NRs, TRIM28 or both. (**E**) Telomere-ChIP analysis of U2OS and WI38-VA13/2RA cells 6 days after COUP-TF2/TR4, TRIM28 or both knockdown to assess enrichment for telomeric DNA with the indicated histones and histone modifications (mean ± SD; *n* = 3 independent biological replicates). (**B**, **E**) Statistical significance is noted as follows: ns*P* > 0.05, *P < 0.05, as determined by the unpaired *t* test. (**C**) Statistical significance is denoted as follows: ns*P* > 0.05, *P < 0.05, **P < 0.01, ***P < 0.001, ****P < 0.0001, as determined by Mann–Whitney *U* test. Source data are available online for this figure.

Next, we examined the ALT-promoting function of TRIM28 in ALT cells. To this end, we depleted TRIM28 from U2OS, SAOS-2, G292, and WI38-VA13/2RA ALT cells by means of siRNAs (Figs. 3F and EV4A) to investigate how TRIM28 is involved in regulating H3K9me3 and ALT activity. Through telomere-ChIP, we detected that telomeric H3K9me3 was significantly reduced upon depleting TRIM28 from these ALT cell lines, supporting that TRIM28 promotes telomeric H3K9me3 in ALT cells (Figs. 3G and EV4B). In addition, we examined whether ALT activity is reduced upon TRIM28 depletion. Indeed, knockdown of TRIM28 from U2OS, SAOS-2, G292 and WI38-VA13/2RA cells reduced their APB formation and ATDS (Figs. 3H and EV4C). We also performed native fluorescence in situ hybridization (FISH) assay to detect single-stranded C-rich telomeric DNA (ssTelC), representing another hallmark of ALT activity (Frank et al, 2022). We found that TRIM28 knockdown limited ssTelC levels in U2OS and WI38-VA13/2RA cells (Fig. 3I), indicative of reduced ALT activity. Together, these findings suggest a role of TRIM28 in promoting ALT by enhancing telomere H3K9me3 in ALT cells.

### Orphan NRs recruit TRIM28 to telomeres

Given that both orphan NRs and TRIM28 promote H3K9me3 at telomeres, we investigated whether they interact epistatically to regulate H3K9me3 at ALT telomeres. In U2OS and WI38-VA13/2RA cells, we depleted orphan NRs (COUP-TF2 and TR4) and TRIM28 individually and in combination using siRNAs, followed by telomere-ChIP analysis (Fig. EV4D,E). Depletion of either orphan NRs or TRIM28 significantly reduced H3K9me3 levels in the ALT cells. However, simultaneous depletion of both did not further decrease H3K9me3 levels (Fig. EV4E). This lack of additivity suggests that orphan NRs and TRIM28 function within the same pathway to regulate telomeric H3K9me3 in ALT cells, indicating an epistatic relationship of orphan NRs and TRIM28.

**Figure 4:**
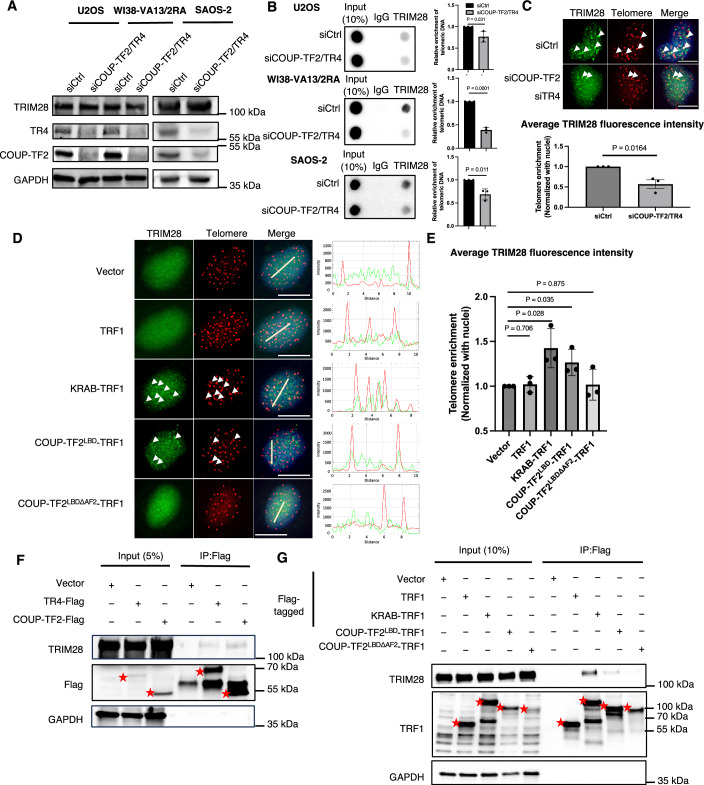
Orphan NRs recruit TRIM28 to telomeres. (**A**) Western blot analysis of TRIM28, COUP-TF2, and TR4 expression in U2OS, WI38-VA13/2RA and SAOS-2 cells following 6 days of treatment with COUP-TF2- and TR4-targeting siRNAs. (**B**) Telomere-ChIP analysis of U2OS, WI38-VA13/2RA and SAOS-2 cells 6 days after NR knockdown to quantify enrichment of TRIM28-associated telomeric DNA. The bar graphs show quantitation of the telomeric DNA pulled down, normalized to control cells (mean ± SD; *n* = 3 independent biological replicates). (**C**) Immunofluorescence images depicting TRIM28 localization at telomeres in WI38-VA13/2RA cells after 6 days of NR-targeting siRNA treatment. The bar chart quantifies the average intensity of TRIM28 in telomeric regions (mean ± SD; *n* = 3 independent biological replicates). (**D**) Representative immunofluorescence images showing TRIM28 localization and telomeres in BJ^T^ cells expressing various plasmids. White arrows indicate foci localized at telomeres. Scale bar, 10 μm. The white lines indicate the region used for fluorescence intensity profiling. Intensity plots across the line section are shown beside each image. (**E**) The bar chart quantifies the average TRIM28 fluorescence intensity in telomeric regions normalized with fluorescence intensity in the nucleus (mean ± SD; *n* = 3 independent biological replicates). (**F**) Co-immunoprecipitation (IP) of TRIM28 with COUP-TF2/TR4. Lysates from WI38-VA13/2RA cells (2 × 10^7^) were subjected to IP with TRIM28 and Flag antibodies and analyzed by Western blot. Red stars indicate the ectopically expressed or immunoprecipitated Flag-tagged COUP-TF2 and TR4 proteins. (**G**) Co-immunoprecipitation of TRIM28 with fusion proteins. Lysates from BJ^T^ cells were subjected to IP with TRIM28 and TRF1 antibodies and analyzed by western blot. Red stars indicate ectopically expressed or immunoprecipitated Flag-tagged TRF1 and TRF1 fusion proteins containing different protein domains. Statistical significance is indicated as follows: ns*P* > 0.05, *P < 0.05, as determined by unpaired *t* test. Source data are available online for this figure.

Furthermore, since TRIM28 is required for orphan NR-induced telomeric H3K9me3 and ALT induction, we wondered if orphan NRs mediate the telomeric localization of TRIM28. To test this possibility, we performed ChIP analysis to examine the association of TRIM28 with telomeres in U2OS, WI38-VA13/2RA, and SAOS-2 cells. Our ChIP results reveal that anti-TRIM28 antibodies pulled down telomere DNA, evidencing the telomeric association of TRIM28 (Fig. 4B). Furthermore, following simultaneous depletion of COUP-TF2 and TR4, the enrichment of TRIM28 at telomeres of the ALT cells was diminished (Fig. 4A,B), supporting that orphan NRs regulate the telomere association of TRIM28 in ALT cells. In addition, by combining immunofluorescence (IF) staining of TRIM28 and telomere-FISH in WI38-VA13/2RA cells, we consistently detected a telomeric localization of TRIM28 and then quantified TRIM28 telomeric enrichment by measuring the signal intensity ratio between the telomere region and the nucleus (Fig. 4C). TRIM28 signal intensity at telomeres was reduced following COUP-TF2 plus TR4 depletion (Fig. 4C). These results indicate that orphan NRs promote TRIM28 telomere localization in ALT cells.

**Figure EV5:**
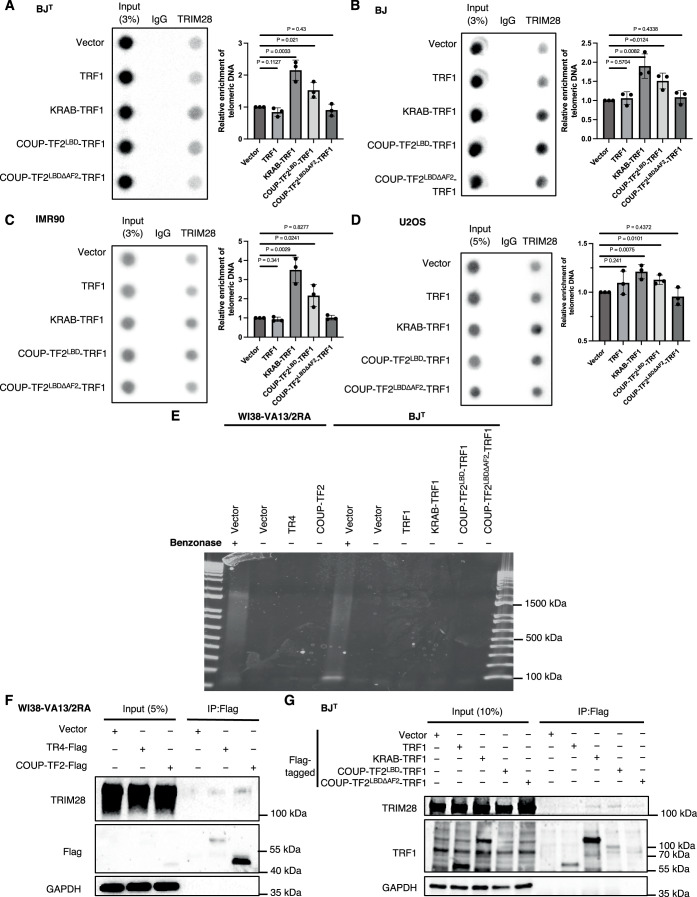
COUP-TF2/TR4-dependent recruitment of TRIM28 to telomeres through DNA-independent interaction. (**A**–**D**) Telomere-ChIP analysis of BJ^T^, BJ, IMR90 and U2OS cells stably expressing fusion proteins to quantify enrichment of TRIM28-associated telomeric DNA. The bar graphs show quantitation of the telomeric DNA pulled down, normalized to control cells (mean ± SD; *n* = 3 independent biological replicates). (**E**) Agarose gel analysis of cell lysates from WI38-VA13/2RA and BJ^T^ cells with or without Benzonase treatment, showing effective DNA removal upon Benzonase digestion, as evidenced by the reduced nucleic acid signal. (**F**) Co-immunoprecipitation (IP) of TRIM28 with COUP-TF2/TR4 in WI38-VA13/2RA cells. Lysates (2 × 10⁷ cells) were treated with Benzonase to degrade DNA, then subjected to IP using TRIM28 or Flag antibodies and analyzed by western blot, demonstrating that the interaction is DNA-independent. (**G**) Co-immunoprecipitation of TRIM28 with TRF1-based fusion proteins in BJ^T^ cells. Lysates were treated with Benzonase, followed by IP with TRIM28 or TRF1 antibodies and Western blot analysis, confirming that TRIM28 interacts with COUP-TF2^LBD^-TRF1 and KRAB-TRF1 in a DNA-independent manner. (**A**–**D**) Statistical significance is noted as follows: ns*P* > 0.05, *P < 0.05, as determined by the unpaired *t* test. Source data are available online for this figure.

Similarly, TRIM28-IF and telomere-FISH detected a telomeric localization for TRIM28 in BJ^T^ cells expressing COUP-TF2^LBD^-TRF1, but not in those expressing vector or TRF1 alone (Fig. 4D,E). Importantly, telomeric tethering of the COUP-TF2 LBD having a truncated AF2 domain (COUP-TF2^LBDΔAF2^-TRF1) in BJ^T^ cells failed to induce TRIM28 telomeric localization (Fig. 4D,E). Since the AF2 domain of orphan NRs is important for recruitment of co-activators (Kruse et al, 2008) and ALT induction (Gaela et al, 2024), this finding supports that the transcriptional regulatory activity of the COUP-TF2 LBD is critical for the telomeric recruitment of TRIM28. Robust telomeric TRIM28 localization was also observed for KRAB-TRF1-expressing cells (Fig. 4D,E), consistent with the direct physical interaction between KRAB and TRIM28 reported previously (Stoll et al, 2022), and serving as a positive control for our experiments. By telomere-ChIP, induction of TRIM28 telomere association by COUP-TF2^LBD^-TRF1 in an AF2 domain-dependent manner was also confirmed in BJ^T^ cells, as well as in primary fibroblasts BJ and IMR90 and U2OS ALT cells (Fig. EV5A–D). Together, these data suggest that telomere binding of orphan NRs can facilitate the localization of TRIM28 to telomeres.

We further investigated whether orphan NRs interact directly with TRIM28 and thereby facilitate TRIM28 telomeric localization. To this end, first, we transiently expressed Flag-tagged COUP-TF2 or TR4 in WI38-VA13/2RA cells for co-immunoprecipitation (Co-IP) using anti-Flag antibodies (Fig. 4F). Western blotting revealed that endogenous TRIM28 co-immunoprecipitated with ectopically expressed COUP-TF2 or TR4 in the WI38-VA13/2RA cells (Fig. 4F), supporting an association between COUP-TF2/TR4 and TRIM28. We also performed Co-IP with anti-Flag antibodies on BJ^T^ cells stably expressing vector, TRF1, KRAB-TRF1, COUP-TF2^LBD^-TRF1, or COUP-TF2^LBDΔAF2^-TRF1, all tagged with the Flag epitope. The Co-IP results demonstrate that TRIM28 is associated with COUP-TF2^LBD^-TRF1, but not TRF1 alone (Fig. 4G). Consistent with its known function, KRAB-TRF1 also co-immunoprecipitated TRIM28 (Fig. 4G). Intriguingly, the ability of COUP-TF2^LBD^-TRF1 to interact with TRIM28 and to promote its telomere localization was dependent on the AF2 domain (Fig. 4G). In addition, pre-incubation of cell lysates with endonuclease to degrade DNA prior to Co-IP did not disrupt TRIM28 association with COUP-TF2 or TR4 in WI38-VA13/2RA and BJ^T^ cells, indicating a DNA-independent interaction between COUP-TF2/TR4 and TRIM28 (Fig. EV5E–G). Together, these findings indicate that orphan NRs may facilitate recruitment of TRIM28 to telomeres through physical interactions.

### The RBCC domain of TRIM28 mediates the COUP-TF2 interaction responsible for telomeric recruitment

**Figure 5:**
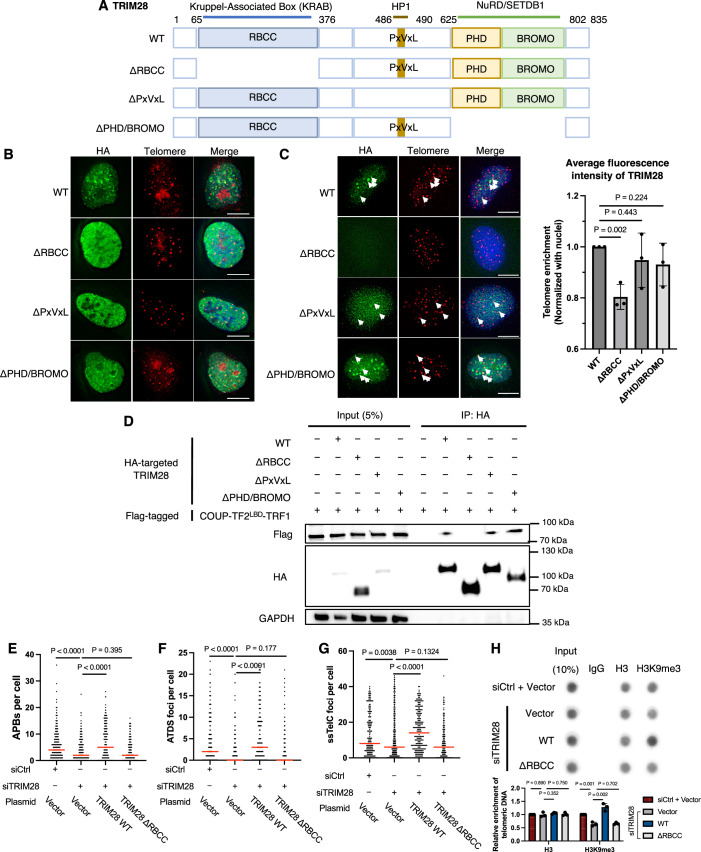
The RBCC domain functions in recruiting TRIM28 to telomeres. (**A**) Schematic representations of wild-type (WT) TRIM28 and its mutant variants. WT TRIM28 comprises an N-terminal RBCC (KRAB binding domain), a central PxVxL (HP1-binding) domain, and a C-terminal PHD/BROMO (NuRD- and SETDB1-interacting) domain. (**B**) Immunofluorescence images showing TRIM28 localization in the nucleus of WI38-VA13/2RA cells following ectopic expression of TRIM28 and its mutants without CSK buffer pretreatment. Scale bar, 10 μm. (**C**) Immunofluorescence images showing TRIM28 localization at telomeres in WI38-VA13/2RA cells after ectopic expression of TRIM28 and its mutants with CSK buffer pretreatment. Scale bar, 10 μm. White arrows indicate foci localized at telomeres. The bar chart quantifies the average signal intensity of TRIM28 in telomeric regions normalized with the intensity in the nucleus (mean ± SD; *n* = 3 independent biological replicates). (**D**) Co-immunoprecipitation of COUP-TF2^LBD^-TRF1 with WT TRIM28 or mutant forms. Lysates from HEK-293T cells were subjected to IP with HA and Flag antibodies and analyzed by Western blot. (**E**) Dot plots quantifying the numbers of APBs in individual WI38-VA13/2RA cells after 6 days of control or TRIM28 knockdown and 2 days of vector, WT TRIM28 or mutant TRIM28 expression (*n* > 150). (**F**) Dot plots quantifying the numbers of EdU+ Telomere (ATDS) foci in individual WI38-VA13/2RA cells after 6 days of control or TRIM28 knockdown and 2 days of vector, WT TRIM28 or mutant TRIM28 expression (*n* > 150). (**G**) The dot plot quantifies numbers of ssTelC foci in individual WI38-VA13/2RA cells after 6 days of control or TRIM28 knockdown and 2 days of vector, WT TRIM28 or mutant TRIM28 expression. ssTelC was detected by native FISH using the TelG PNA probe (*n* > 150). (**H**) Telomere-ChIP analysis of WI38-VA13/2RA cells after 6 days of control or TRIM28 knockdown and 2 days of vector, WT TRIM28 or mutant TRIM28 expression to assess enrichment for telomeric DNA with specific histones and histone modifications. Bar graphs show quantitation of the telomeric DNA pulled down, normalized to input and control cells (mean ± SD; *n* = 3 independent biological replicates). (**C**, **H**) Statistical significance is noted as follows: ns*P* > 0.05, *P < 0.05, **P < 0.01, as determined by the unpaired *t* test. (**E**–**G**) Statistical significance is indicated as follows: ns*P* > 0.05, *P < 0.05, **P < 0.01), ***P < 0.001, ****P < 0.0001, as determined by Mann–Whitney *U* test. Source data are available online for this figure.

To determine the domain(s) of TRIM28 responsible for its telomeric recruitment and ALT-promoting functions, we constructed plasmids to express wild-type TRIM28 protein or mutant variants lacking either the RING Finger-B Box-Coiled Coil (RBCC), PxVxL, or plant homeodomain and bromodomain (PHD/BROMO) domain (Fig. 5A). The RBCC domain mediates the interaction between TRIM28 and KRAB domain-containing zinc finger proteins, facilitating TRIM28’s association with specific genomic loci (Wolf et al, 2015). The PxVxL domain is responsible for binding heterochromatin protein 1 (HP1), whereas the PHD/BROMO domain recruits SETDB1 methyltransferase and the NuRD complex, both essential for heterochromatin formation and maintenance (Wolf et al, 2015). We ectopically expressed individual TRIM28 proteins hosting an HA tag in WI38-VA13/2RA cells and then assessed their expression levels and cellular localization by IF-based staining of samples directly fixed with paraformaldehyde. We found that the ectopically expressed wild-type and mutant TRIM28 proteins primarily localized in the nucleus (Fig. 5B), indicating that the RBCC, PxVxL, and PHD/BROMO domains are not required for TRIM28 nuclear localization. To further investigate the chromatin or telomere localization of the TRIM28 proteins, we isolated cytoplasmic and nuclear soluble fractions by incubating the samples with permeabilization buffer containing 0.5% Triton X-100 prior to fixation for IF-telomere FISH (Fig. 5C). The results revealed significant retention of wild-type TRIM28 signal in the nucleus, with puncta co-localizing with telomeres (Fig. 5C), confirming the association of TRIM28 with chromatin and telomeres in WI38-VA13/2RA cells shown in Fig. 4B,C. However, deletion of the TRIM28 RBCC domain abolished its nuclear localization and telomeric enrichment (Fig. 5C), revealing that the RBCC domain is required for the TRIM28 chromatin association. This result is consistent with the finding previously that TRIM28 associates with chromatin by interacting through its RBCC domain with DNA-binding factors (Wolf et al, 2015). Intriguingly, deletion of the PxVxL domain or PHD/BROMO domain did not affect TRIM28 telomeric enrichment (Fig. 5C), so the co-factor recruitment associated with these domains may not be essential for TRIM28’s chromatin or telomeric localization.

Thus, the RBCC domain is required for TRIM28 to localize at telomeres, indicating that it may also mediate the TRIM28 and COUP-TF2 interaction. To address this possibility, we transiently expressed Flag-tagged COUP-TF2^LBD^-TRF1 with HA-tagged wild-type or mutant TRIM28 in HEK-293T cells for Co-IP using anti-HA antibodies. The Co-IP results revealed that COUP-TF2^LBD^-TRF1 interacts with wild-type TRIM28, as well as with TRIM28 lacking the PxVxL or PHD/BROMO domains, but not with TRIM28 lacking the RBCC domain (Fig. 5D), supporting that the RBCC domain is necessary for the interaction between COUP-TF2^LBD^ and TRIM28. Collectively, these findings provide evidence that TRIM28 is recruited to telomeres by COUP-TF2 through interaction between the RBCC domain of TRIM28 and the LBD of COUP-TF2.

**Figure EV6:**
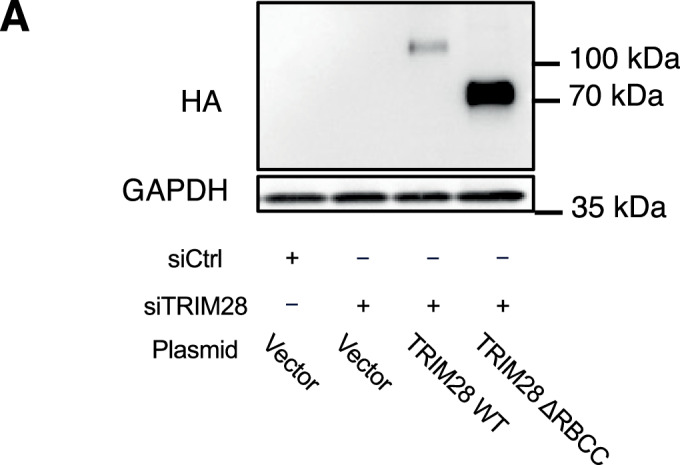
Western blot analysis of mutant TRIM28 expression in WI38-VA13/2RA cells. (**A**) Western blot analysis of WI38-VA13/2RA cells after 6 days of TRIM28 knockdown and 2 days of WT TRIM28 or mutant TRIM28 expression, confirming expression of the constructs using an anti-HA antibody. Source data are available online for this figure.

Since the RBCC domain of TRIM28 appears to be critical for recruitment of the protein to telomeres, we anticipated its loss would impair TRIM28’s role in maintaining ALT features and telomeric H3K9me3 levels. To test this hypothesis, we ectopically expressed vector control, wild-type TRIM28 or RBCC-deleted TRIM28 in WI38-VA13/2RA cells from which endogenous TRIM28 had been depleted by means of siRNAs (Fig. EV6A), and then assessed the impact on ALT phenotypes. Expression of wild-type TRIM28 restored APB formation, ATDS, ssTelC, and telomeric H3K9me3 levels in those cells, whereas RBCC-deleted TRIM28 failed to do so (Fig. 5E–H). These findings demonstrate that the RBCC domain of TRIM28 is essential for its function in mediating ALT induction and telomeric chromatin modifications, underscoring the crucial role of the RBCC domain in facilitating TRIM28 telomeric recruitment by COUP-TF2.

## Discussion

**Figure 6:**
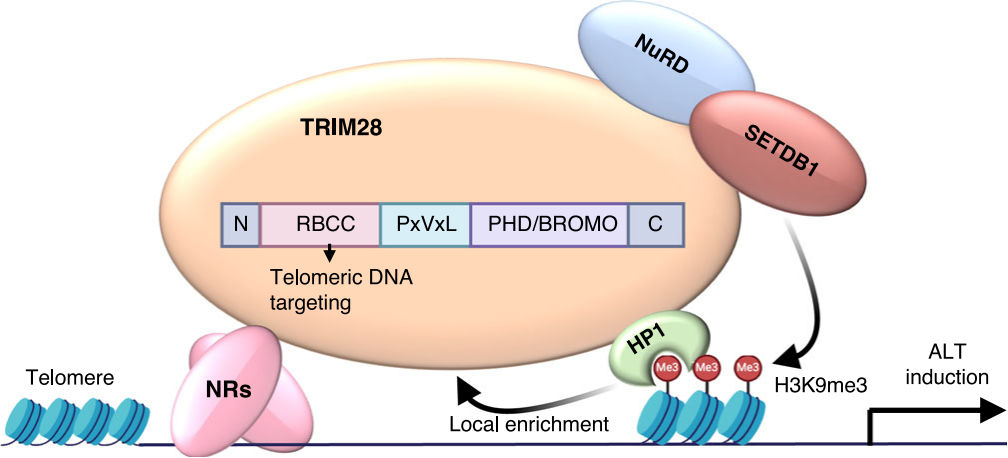
TRIM28 mediates orphan NR-induced H3K9me3 formation ALT activation. Orphan NRs interact with TRIM28 for its recruitment to telomeres, enabling NuRD, SETDB1, and HP1 to act on telomeres to promote epigenetic alterations — including H3K9me3 modifications — and enforce heterochromatin formation for ALT activation.

We have demonstrated previously that orphan NRs initiate APB formation and activate ALT pathways when binding to telomeres (Gaela et al, 2024). However, the specific role of chromatin modifications, particularly H3K9me3, in this process had not been defined. Herein, we reveal that targeting the COUP-TF2^LBD^ to telomeres in BJ^T^ fibroblasts enhances telomeric H3K9me3 levels (Fig. 1F). Conversely, depletion of COUP-TF2 or TR4 in ALT cells (U2OS, SAOS-2, G292, and WI38-VA13/2RA) reduces telomeric H3K9me3 (Figs. 1B and EV1B). These results establish a direct link between orphan NR-mediated ALT activation and telomeric enrichment of H3K9me3. Mechanistically, we show that orphan NRs recruit TRIM28 to telomeres to promote H3K9me3 and ALT activity (Figs. 3, 4, EV4, and EV5). This finding is consistent with prior studies reporting that TRIM28 preferentially localizes to ALT telomeres for H3K9me3 enrichment (Czerwińska et al, 2017; Wang et al, 2021). Crucially, we demonstrate here that the interaction between orphan NRs and TRIM28 depends on the RBCC domain of TRIM28 (Fig. 5D). This domain is essential for TRIM28’s recruitment to telomeres, its interaction with COUP-TF2, and its role in facilitating ALT induction and telomeric H3K9me3 activation (Fig. 5C–H). Since SETDB1 functions as a cofactor of TRIM28 and is recruited by it to establish the H3K9me3 mark (Wang et al, 2021), we propose that orphan NRs recruit TRIM28, which in turn interacts with SETDB1 to promote telomeric H3K9me3. Supporting this hypothesis, we found that SETDB1 depletion significantly reduced COUP-TF2-mediated ALT phenotypes (Fig. EV3C,D). Beyond its role in H3K9me3 activation, TRIM28 interacts with other chromatin regulators, including HP1α/β/γ, HDAC1/2, and DNMT1, suggesting a more extensive role in chromatin remodeling (Czerwińska et al, 2017). These interactions may coordinate additional modifications, such as histone deacetylation or DNA methylation, to create a chromatin environment conducive to ALT maintenance (Fig. 6). Further studies are needed to elucidate any additional chromatin-regulatory roles of TRIM28 in orchestrating orphan NR-driven chromatin remodeling and ALT pathway activation at telomeres. It is also worth exploring whether orphan NRs and TRIM28 have a broader collaborative role in global genomic regulation.

H3K9me3, a hallmark of heterochromatin, is significantly enriched at ALT telomeres compared to non-ALT telomeres, defining a distinct feature of the chromatin landscape in ALT cells (Episkopou et al, 2014; Gauchier et al, 2019). While H3K9me3 is known for its role in chromatin compaction, its specific contribution to ALT activation remains incompletely understood. A key function of H3K9me3 is to recruit heterochromatin protein 1 (HP1), a family of proteins integral to heterochromatin formation and maintenance (Bannister et al, 2001; Lachner et al, 2001; Sims et al, 2003). All three HP1 isoforms—HP1α, HP1β, and HP1γ—have been shown to colocalize with APBs (Jiang et al, 2009). Notably, depletion of HP1α or HP1γ from the ALT cell line IIICF-T/B3 leads to significantly reduced APB formation, indicating essential roles for both isoforms in organizing ALT-specific chromatin structures (Jiang et al, 2009). Beyond chromatin association, HP1 proteins mediate liquid–liquid phase separation (LLPS), a critical process in heterochromatin organization (Larson et al, 2017; Tortora et al, 2023). Given that APB formation is triggered by LLPS processes (Banani et al, 2016; Corpet et al, 2020) and requires HP1 (Jiang et al, 2009), we propose that HP1 facilitates APB assembly at ALT telomeres through LLPS mechanisms. This model suggests that the enrichment of H3K9me3 at ALT telomeres promotes APB formation by serving as a scaffold for HP1 recruitment and LLPS-driven chromatin condensation. In addition, HP1 interacts with TRIM28 through its PxVxL motif and plays a pivotal role in stabilizing the TRIM28-containing chromatin complex, which reinforces further H3K9me3 and the establishment of heterochromatin (Czerwińska et al, 2017; Sripathy et al, 2006). Future studies could investigate whether the interaction between TRIM28 and HP1 plays a regulatory role in APB formation and subsequent ALT activation.

In our study, expression of COUP-TF2^LBD^-TRF1 and KRAB-TRF1 in human fibroblasts induced telomeric H3K9me3 and APB formation, demonstrating their ability to alter telomeric chromatin and initiate ALT activity. However, their effectiveness in promoting telomeric DNA synthesis differs significantly. Despite both constructs inducing comparable levels of APB formation (~60%), KRAB-TRF1 promoted ATDS in less than 8% of the cells, whereas COUP-TF2^LBD^-TRF1 induced ATDS in approximately 20% of the cells (Figs. 2C,D and 3D,E). This disparity may arise from the following possibilities. First, the KRAB-TRF1 fusion protein effectively compacts telomeric chromatin, as indicated by increased histone H3 and H4 occupancy (Fig. 2B), which likely impedes access by the recombination machinery and thus limits DNA synthesis. In contrast, COUP-TF2^LBD^-TRF1 leaves histone occupancy unchanged (Fig. 1F), maintaining an accessible chromatin state that supports telomeric DNA synthesis. Second, ATRX, an ALT suppressor, is recruited to telomeres in an H3K9me3-dependent manner (Gauchier et al, 2019). Since KRAB-TRF1 induces greater enrichment of telomeric H3K9me3 than COUP-TF2^LBD^-TRF1 (Figs. 1F and 2B), it may also recruit more ATRX, thereby more effectively inhibiting ALT activity. Third, since COUP-TF2 is a transcription factor, it may recruit additional co-activators (Kruse et al, 2008; Xu et al, 2019) to promote ALT activity. Together, these findings emphasize that though moderate telomeric H3K9me3 enrichment is positively associated with ALT activation, excessive H3K9me3 enrichment or chromatin compaction can hinder telomeric DNA synthesis. This scenario highlights the importance of considering multiple chromatin features, such as histone modifications, chromatin accessibility, and factor recruitment, to understand chromatin’s role in ALT regulation rather than relying on a single characteristic.

Orphan NRs have been shown to promote the recruitment of the ZNF827-NuRD complex to ALT telomeres (Conomos et al, 2014); however, the molecular mechanism remains unclear. TRIM28 interacts with KRAB-domain zinc-finger proteins (Czerwińska et al, 2017). Thus, it is plausible that TRIM28 may act as a protein hub by binding to both orphan NRs and ZNF827, thereby bridging the telomeric recruitment of the NuRD complex and other chromatin remodeling factors for ALT chromatin regulation. Future investigations should examine whether TRIM28 is required for orphan NR-mediated targeting of the ZNF827-NuRD complex to telomeres. Moreover, additional zinc-finger proteins, such as ZNF524 and ZBTB40 (BTB/POZ-domain zinc-finger protein), have been implicated in ALT telomere (Braun et al, 2023; Zhou et al, 2023). Thus, it would be valuable to determine if TRIM28 similarly regulates telomeric recruitment of ZNF524 and ZBTB40, potentially uncovering broader mechanisms underlying ALT-dependent telomere maintenance.

## Methods

**Table:** Reagents and tools table

Reagent/resource	Reference or source	Identifier or catalog number
**Experimental models**		
WI38-VA13/2RA	ATCC	CCL-75.1
WI38-VA13/2RA-Vector	This study	
WI38-VA13/2RA-TRF1	This study	
WI38-VA13/2RA-KRAB-TRF1	This study	
WI38-VA13/2RA-VP64-TRF1	This study	
WI38-VA13/2RA-TRIM28 WT	This study	
WI38-VA13/2RA-TRIM28 ΔRBCC	This study	
WI38-VA13/2RA-TRIM28 ΔPxVxL	This study	
WI38-VA13/2RA-TRIM28 ΔPHD/BROMO	This study	
U2OS	ATCC	HTB-96
U2OS-Vector	This study	
U2OS-TRF1	This study	
U2OS-COUP-TF2^LBD^-TRF1	This study	
U2OS-COUP-TF2^LBDΔAF2^-TRF1	This study	
U2OS-KRAB-TRF1	This study	
SAOS-2	ATCC	HTB-85
G292	ATCC	CRL-1423
BJ^T^	Gaela et al, 2024	
BJ^T^-Vector	Gaela et al, 2024	
BJ^T^-TRF1	Gaela et al, 2024	
BJ^T^-COUP-TF2^LBD^-TRF1	Gaela et al, 2024	
BJ^T^-COUP-TF2^LBDΔAF2^-TRF1	Gaela et al, 2024	
BJ^T^-KRAB	This study	
BJ^T^-KRAB-TRF1	This study	
BJ^T^-VP64	This study	
BJ^T^-VP64-TRF1	This study	
BJ	ATCC	CRL-2522
BJ-Vector	This study	
BJ-TRF1	This study	
BJ-COUP-TF2^LBD^-TRF1	Gaela et al, 2024	
BJ-COUP-TF2^LBDΔAF2^-TRF1	Gaela et al, 2024	
BJ-KRAB-TRF1	This study	
BJ-VP64-TRF1	This study	
IMR90	ATCC	CCL-186
IMR90-Vector	This study	
IMR90-TRF1	This study	
IMR90-COUP-TF2^LBD^-TRF1	Gaela et al, 2024	
IMR90-COUP-TF2^LBDΔAF2^-TRF1	Gaela et al, 2024	
IMR90-KRAB-TRF1	This study	
IMR90-VP64-TRF1	This study	
HEK293T	ATCC	CRL-3216
**Recombinant DNA**		
pCL-Ampho	Gaela et al, 2024	
pCL-Vector	Gaela et al, 2024	
pCL-TRF1	Gaela et al, 2024	
pCL-COUP-TF2^LBD^-TRF1	Gaela et al, 2024	
pCL-COUP-TF2^LBDΔAF2^-TRF1	Gaela et al, 2024	
pCL-KRAB	This study	
pCL-KRAB-TRF1	This study	
pCL-VP64	This study	
pCL-VP64-TRF1	This study	
pCL-TRIM28 WT	This study	
pCL-TRIM28 ΔRBCC	This study	
pCL-TRIM28 ΔPxVxL	This study	
pCL-TRIM28 ΔPHD/BROMO	This study	
pCL-COUP-TF2	Gaela et al, 2024	
pCL-TR4	Gaela et al, 2024	
**Antibodies**		
Anti-COUP-TF2	R&D Systems	PP-H7147-00
Anti-TR4	R&D Systems	PP-H0107B-00
Anti-TRF1	GeneTex	90549
Anti-PML	Merck (MilliporeSigma in USA)	05-718
Anti-TRIM28	Proteintech	15202-1-AP
Anti-HP1α/β/γ	Santa Cruz	sc-515341
Anti-Flag	Sigma-Aldrich	F3165
Anti-HA	Thermo Fisher Scientific	26183
Anti-GAPDH	Proteintech	HRP60004
Anti-Histone 3	Abcam	ab1791
Anti-Histone 3 lysine 9 trimethylation	Abcam	ab8898
Anti-Histone H4	Cell Signaling Technology	14149S
Anti-acetyl-Histone H4	Merck (MilliporeSigma in USA)	06-866
Anti-Histone H3 lysine 27 trimethylation	Cell Signaling Technology	9733T
Anti-Histone H4 lysine 20 trimethylation	Abcam	ab9053
IgG	Merck (MilliporeSigma in USA)	PP64
Protein G Sepharose™ 4 Fast Flow	Cytiva	17-0618-01
ANTI-FLAG® M2 Affinity Gel (agarose beads)	Sigma-Aldrich	A2220
**Oligonucleotides and other sequence-based reagents**	**Source**	**Sequence (5’-3’)**
siRNA 1 target COUP-TF2	Dharmacon (On-TARGETplus)	CCAACCAGCCGACGAGAUU
siRNA 2 target COUP-TF2	Dharmacon (On-TARGETplus)	GCGAGCUGUUUGUGUUGAA
siRNA 3 target COUP-TF2	Dharmacon (On-TARGETplus)	ACUCGUACCUGUCCGGAUA
siRNA 4 target COUP-TF2	Dharmacon (On-TARGETplus)	AGUCAUAGAGCAAUUGUUU
siRNA 1 target TR4	Dharmacon (On-TARGETplus)	GCACUGGGCUCGGUCAAUC
siRNA 2 target TR4	Dharmacon (On-TARGETplus)	GAUAGCUACUCCCACGUUU
siRNA 3 target TR4	Dharmacon (On-TARGETplus)	GCAGGACUAUGUUCAGAAA
siRNA 4 target TR4	Dharmacon (On-TARGETplus)	AAGAUAAACUUUCUGGUGA
siRNA 1 target TRIM28	Dharmacon (On-TARGETplus)	GAAAUGUGAGCGUGUACUG
siRNA 2 target TRIM28	Dharmacon (On-TARGETplus)	GCGAUCUGGUUAUGUGCAA
siRNA 3 target TRIM28	Dharmacon (On-TARGETplus)	GAACGAGGCCUUCGGUGAC
siRNA 4 target TRIM28	Dharmacon (On-TARGETplus)	AGACAGCACUGGCGUGGUG
siRNA 1 target SETDB1	Dharmacon (On-TARGETplus)	AAGAUGGGCUUUCAUGUUA
siRNA 2 target SETDB1	Dharmacon (On-TARGETplus)	GAACUAAGACUUGGCACAA
siRNA 3 target SETDB1	Dharmacon (On-TARGETplus)	GGGAUCAACCAGACAUAUA
siRNA 4 target SETDB1	Dharmacon (On-TARGETplus)	GAGCGCACCUGUUCGUAAG
siRNA 1 target HP1α	Dharmacon (On-TARGETplus)	GGAUUGCCCUGAGCUAAUU
siRNA 2 target HP1α	Dharmacon (On-TARGETplus)	UAGACAGGCGCGUGGUUAA
siRNA 3 target HP1α	Dharmacon (On-TARGETplus)	GGGAGAAGUCAGAAAGUAA
siRNA 4 target HP1α	Dharmacon (On-TARGETplus)	GAAAGAAACAGCAAAGAGC
siRNA 1 target HP1β	Dharmacon (On-TARGETplus)	GCCCACAGGUUGUCAUAUC
siRNA 2 target HP1β	Dharmacon (On-TARGETplus)	GAACUAACGCUCCUGAGUA
siRNA 3 target HP1β	Dharmacon (On-TARGETplus)	GAGAGGAGAGCAAACCAAA
siRNA 4 target HP1β	Dharmacon (On-TARGETplus)	GAAAACAGCACAUGAGACA
siRNA 1 target HP1γ	Dharmacon (On-TARGETplus)	AGUACUAGAUCGACGUGUA
siRNA 2 target HP1γ	Dharmacon (On-TARGETplus)	CAGAAUUGAUUGAAGCGUU
siRNA 3 target HP1γ	Dharmacon (On-TARGETplus)	AGACAGCAGUGGAGAAUUG
siRNA 4 target HP1γ	Dharmacon (On-TARGETplus)	AAUAUGAAGUGUCCUCAAA
siRNA 1 target HDAC1	Dharmacon (On-TARGETplus)	ACUAUGGUCUCUACCGAAA
siRNA 2 target HDAC1	Dharmacon (On-TARGETplus)	GCAAGUAUUAUGCUGUUAA
siRNA 3 target HDAC1	Dharmacon (On-TARGETplus)	CCGGUCAUGUCCAAAGUAA
siRNA 4 target HDAC1	Dharmacon (On-TARGETplus)	CCACAGCGAUGACUACAUU
siRNA 1 target HDAC2	Dharmacon (On-TARGETplus)	GCGGAUAGCUUGUGAUGAA
siRNA 2 target HDAC2	Dharmacon (On-TARGETplus)	GCAAAGAAAGCUAGAAUUG
siRNA 3 target HDAC2	Dharmacon (On-TARGETplus)	GAUAACAUGUCUGAGUAUA
siRNA 4 target HDAC2	Dharmacon (On-TARGETplus)	GAUCGUGUAAUGACGGUAU
siRNA 1 target DNMT1	Dharmacon (On-TARGETplus)	GCACCUCAUUUGCCGAAUA
siRNA 2 target DNMT1	Dharmacon (On-TARGETplus)	AUAAAUGAAUGGUGGAUCA
siRNA 3 target DNMT1	Dharmacon (On-TARGETplus)	CCUGAGCCCUACCGAAUUG
siRNA 4 target DNMT1	Dharmacon (On-TARGETplus)	GGACGACCCUGACCUCAAA
TERRA-specific RT primer	Feretzaki and Lingner, 2017	CCCTAACCCTAACCCTAACCCTAACCCTAA
TERRA forward primer set 1	Feretzaki and Lingner, 2017	ATGCACACATGACACACACTAAA
TERRA reverse primer set 1	Feretzaki and Lingner, 2017	TACCCGAACCTGAACCCTAA
TERRA forward primer set 2	Feretzaki and Lingner, 2017	GCAAATGCAGCAGTCCTAATG
TERRA reverse primer set 2	Feretzaki and Lingner, 2017	GACCCTGACCCTAACCCTAA
TERRA forward primer set 3	Feretzaki and Lingner, 2017	CCTGCGCACCGAGATTCT
TERRA reverse primer set 3	Feretzaki and Lingner, 2017	GCACTTGAACCCTGCAATACAG
GAPDH-specific RT-primer	Feretzaki and Lingner, 2017	GCCCAATACGACCAAATCC
SETDB1 forward primer	This study	GCCTACAGCAAGGAACGTATCC
SETDB1 reverse primer	This study	GTTGATGGCAGGCACACTTGGA
HP1α forward primer	This study	CAATTTCTCAAACAGTGCCGA
HP1α reverse primer	This study	CACCACAGGAATCTGTTGCC
HDAC1 forward primer	This study	GGTCCAAATGCAGGCGATTCCT
HDAC1 reverse primer	This study	TCGGAGAACTCTTCCTCACAGG
HDAC2 forward primer	This study	CTCATGCACCTGGTGTCCAGAT
HDAC2 reverse primer	This study	GCTATCCGCTTGTCTGATGCTC
DNMT1 forward primer	This study	GATTTGTCCTTGGAGAACGGTG
DNMT1 reverse primer	This study	TGAGATGTGATGGTGGTTTGCC
GAPDH forward primer	This study	GGATTTGGTCGTATTGGG
GAPDH reverse primer	This study	GGAAGATGGTGATGGGATT
**Chemicals, enzymes and other reagents**		
Goat anti-Mouse IgG (H + L) Secondary Antibody, HRP	Thermo Fisher Scientific	31430
Goat anti-Rabbit IgG (H + L) Secondary Antibody, HRP	Thermo Fisher Scientific	31460
SuperSignal™ West Femto	Thermo Fisher Scientific	34096
Lipofectamine RNAiMax	Thermo Fisher Scientific	13778150
Lipofectamine 3000	Thermo Fisher Scientific	L3000015
SYBR Select Master Mix	Thermo Fisher Scientific	4472919
iScript cDNA Synthesis Kit	Bio-Rad	170-8891
SuperScript™ III Reverse Transcriptase	Invitrogen	LS18080085
In-Fusion® Snap Assembly Value Bundle (In-Fusion HD Cloning)	Clontech	638946 (638910)
Click-iT Plus EdU Alexa Fluor 647 Imaging Kit	Invitrogen	C10640
PNA probeTelC-Cy5	Panagene	F1003-5
PNA probeTelC-TMR	Panagene	F2001-5
PNA probeTelG-FITC	Panagene	F1010-5
DAPI Nucleic Acid Stains	Invitrogen	D1306
ProLong Gold antifade Reagent	Thermo Fisher Scientific	P36934
cOmplete™, EDTA-free Protease Inhibitor Cocktail	Roche	04693132001
Benzonase nuclease	Sigma-Aldrich	E1014-5KU
RNase	Invitrogen	12091021
Proteinase K	Merck (MilliporeSigma in USA)	1.24568.0500
T4 Polynucleotide Kinase (T4 PNK)	NEB	M0201S
QIAquick PCR Purification Kit (250)	Qiagen	28106
Dulbecco’s modified Eagle’s medium (DMEM)	Thermo Fisher Scientific	11995-040
Fetal Bovine Serum (FBS)	Thermo Fisher Scientific	26140079
Penicillin-Streptomycin	Gibco	15140-122
Minimum Essential Medium (MEM)	Thermo Fisher Scientific	11095114
McCoy’s 5 A medium	Thermo Fisher Scientific	16600-082
RNASpin Mini Kit	Cytiva	25-0500-72
Thymidine	Sigma-Aldrich	T1895
CDK1i (RO-3306)	Selleckchem	S7747
**Software**		
GraphPad Prism 9	GraphPad Software	
FIJI	ImageJ distribution, NIH	
ilastik	Berg et al, 2019	

### Cell lines

BJ^T^ cells, derived from BJ normal fibroblasts stably expressing hTERT, and the human osteosarcoma U2OS and human embryonic kidney HEK-293T cells, were cultured in Dulbecco’s Modified Eagle’s Medium (DMEM) supplemented with 10% fetal bovine serum (FBS) and 0.5% penicillin/streptomycin. BJ, IMR90, and G292 cells were also cultured in DMEM under the same conditions. SV40-transformed WI38-VA13/2RA fibroblasts were cultured in Minimum Essential Medium (MEM) containing 10% FBS and 0.5% penicillin/streptomycin. SAOS-2 cells were cultured in McCoy’s 5A medium supplemented with 10% FBS and 0.5% penicillin/streptomycin. All cell lines were maintained at 37 °C in a humidified atmosphere with 5% CO₂.

Retroviral transduction was used to generate cell lines stably expressing specific proteins. BJ^T^ cells were transduced with Flag-tagged constructs, including vector control, TRF1, KRAB-TRF1, VP64-TRF1, COUP-TF2^LBD^-TRF1, COUP-TF2^LBDΔAF2^-TRF1, COUP-TF2, and TR4. HA-tagged constructs of full-length TRIM28 (amino acids 1–835), TRIM28ΔRBCC (lacking amino acids 65–376), TRIM28ΔPxVxL (lacking amino acids 486–490), and TRIM28ΔPHD/BROMO (lacking amino acids 625–802) were stably expressed in WI38-VA13/2RA fibroblasts. The TRIM28 plasmid was a gift from Ching-Jin Chang (Chang et al, 2023).

### RNA interference

The siRNAs used in this study were On-TARGETplus reagents (Dharmacon) containing individual or a mixture of four siRNA duplexes generated by the SMARTselection algorithm, with chemical modifications to eliminate off-target effects. Cells were transfected with 25 nM siRNAs using Lipofectamine RNAiMAX following the manufacturer’s instructions. Four siRNAs for each of COUP-TF2, TR4, SETDB1, HP1α/β/γ, HDAC1/2, DNMT1, and TRIM28 were employed. After transfection, cells underwent two rounds of siRNA treatment over a 6-day incubation period before being subjected to subsequent analyses.

### Protein extraction and western blotting

Whole-cell extracts were prepared using 1× SDS sample buffer containing 62.5 mM Tris-HCl (pH 6.8), 10% (v/v) glycerol, 2% (w/v) SDS, 0.01% (w/v) bromophenol blue, and 10% (v/v) β-mercaptoethanol. The extracts were denatured by heating at 90 °C for 10 min. Denatured protein samples were resolved on SDS-PAGE gels and electrophoretically transferred to nitrocellulose membranes (Amersham™, GE Healthcare). The membranes were blocked for 1 h at 25 °C in Tris-buffered saline (pH 8.0) containing 5% (w/v) dry milk and 0.1% (v/v) Tween-20. Subsequently, membranes were incubated overnight at 4 °C with primary antibodies. The primary antibodies used included anti-COUP-TF2, anti-TR4, anti-Flag, anti-HP1, anti-TRIM28, anti-TRF1, anti-HA, and anti-GAPDH. After washing, the membranes were incubated with horseradish peroxidase (HRP)-conjugated secondary antibodies. Protein bands were detected using SuperSignal West Femto Maximum Sensitivity Substrate and visualized via chemiluminescence.

### Detection of telomeric repeat-containing RNA (TERRA)

To detect TERRA expression, total RNA was isolated from BJ^T^ cells using a RNASpin Mini kit according to the manufacturer’s instructions. DNase treatments were performed to remove residual genomic DNA, ensuring high RNA purity. Complementary DNA (cDNA) synthesis was carried out using SuperScript III Reverse Transcriptase with a TERRA-specific RT primer and a housekeeping gene-specific RT primer. The RNA isolation, DNase treatment, and cDNA synthesis procedures were adapted from an established protocol described by Feretzaki & Lingner (Feretzaki and Lingner, 2017).

Quantitative real-time PCR (qPCR) was performed using the Applied Biosystems 7500 Fast Real-Time PCR system (Life Technologies) with a reaction mixture containing 1× SYBR Green Select Master Mix, cDNA, and specific primer pairs. Primers targeting subtelomeric regions of human chromosomes were designed based on the protocol of the previous study (Feretzaki and Lingner, 2017) and validated using the T2T CHM13v2.0/hs1 genome dataset from Genome Browser (https://hgdownload.soe.ucsc.edu/goldenPath/hs1/bigZips/) to ensure specificity and accuracy. The primer sequences were as follows: primer set 1 was designed to target the q-arm of chromosome 10; primer set 2 was designed to target the q-arm of chromosome 15; primer set 3 was designed to target multiple subtelomeric regions (5q, 7q, 9q, 10q, 13q, 16q, 20q and 22q).

### Real-time RT-PCR

Total cellular RNA was extracted using an RNASpin Mini kit following the manufacturer’s instructions. RNA samples were reverse-transcribed into cDNA using iScript Reverse Transcriptase Supermix. Real-time PCR analyses were conducted on an Applied Biosystems 7500 Fast Real-Time PCR system (Life Technologies) using a reaction mixture containing 1× SYBR Green Select Master Mix, cDNA, and specific primer pairs. Cycle threshold (Ct) values were normalized to GAPDH, which was used as an internal control. Relative gene expression levels were calculated using the comparative Ct (ΔΔCt) method.

### Detection of telomeric DNA synthesis

To synchronize cells in the G2 phase, a 21-h treatment with 2 mM thymidine was applied, followed by a 4-h release into fresh medium. Subsequently, cells were exposed to 15 µM CDK1 inhibitor for 12 h. To detect DNA synthesis, cells were incubated with 20 µM EdU for 3 h, after which EdU incorporation was visualized using a Click-iT reaction with fluorescently-labeled picolylazide.

### Immunofluorescence (IF) and fluorescence in situ hybridization (FISH)

Cells grown on coverslips within 12-well plates were fixed using 4% paraformaldehyde for 10 min. Cells were optionally pre-extracted with CSK buffer (10 mM PIPES, pH 6.8; 300 mM sucrose; 100 mM NaCl; 3 mM MgCl₂; 1 mM EGTA; 0.5% Triton X-100) to remove soluble proteins. Subsequently, all cells were permeabilized with 0.5% Triton X-100 in PBS for 5 min. The cells were then blocked in 2% FBS diluted in PBS for 30 min and incubated with anti-Flag or anti-PML primary antibodies for 1 h. Then, Alexa 488-conjugated or Alexa 568-conjugated secondary antibodies diluted in 2% FBS were applied for another hour. After immunostaining, the cells were re-fixed with 4% paraformaldehyde for 10 min and dehydrated through a series of ethanol treatments (70%, 95%, and 100%) for 5 min each. Once air-dried, the coverslips were subjected to hybridization using a mixture containing 10 mM Tris–HCl (pH 7.5), 70% formamide, 10% blocking reagent (Roche), and 0.25 μM TMR-conjugated (CCCTAA)₃ telomere PNA probe. Denaturation was conducted at 80 °C for 3 min on a heat plate, followed by overnight hybridization at room temperature. Post-hybridization washes consisted of two rinses with wash buffer A (70% formamide, 10 mM Tris–HCl, pH 7.5) and three with wash buffer B (10 mM Tris–HCl, pH 7.5, 4 M NaCl, 0.08% Tween-20). The nuclei were stained with DAPI, followed by a second ethanol dehydration series and air-drying of the coverslips. Finally, the coverslips were mounted using ProLong Gold Antifade Mountant.

### Telomere-ChIP

Cells were cultured in 15-cm dishes and cross-linked with 1% formaldehyde at room temperature for 10 min. The reaction was quenched by washing the cells twice with PBS containing 200 mM glycine (PBS/Glycine). The cells were scraped into 5 mL of PBS/Glycine, centrifuged at 4000 rpm, and washed with PBS. The cell pellet was resuspended in 1 mL of Swelling Buffer (25 mM HEPES, pH 7.9, 0.25% Triton X-100, 10 mM KCl, 1.5 mM MgCl2, 1 mM EDTA, 1 mM DTT + protease inhibitor cocktail), incubated on ice for 10 min, and centrifuged at 4000 rpm. The pellet was resuspended in 600 μL of sonication buffer (50 mM HEPES, pH 7.9, 150 mM NaCl, 1 mM EDTA, 0.1% Na deoxycholate, 0.1% SDS, 1% Triton X-100 + protease inhibitor cocktail) and subjected to 40 cycles of sonication (30 s on, 30 s off) using a Bioruptor. The lysate was clarified by centrifugation at maximum speed for 15 min, and the supernatant was divided into five 120 μL aliquots.

For immunoprecipitation (IP), the supernatant was diluted four-fold with IP dilution buffer (16.7 mM HEPES, 150 mM NaCl, 1 mM EDTA, 0.1% sodium deoxycholate, 0.1% SDS, 1% Triton X-100, and protease inhibitors) and incubated overnight at 4 °C with 5 μg of antibody and 20 μg of bacterial DNA, reserving 5–10% of the IP mix as input. The following day, 40 μL of Protein G Sepharose preincubated with 5 μg of bacterial DNA and 30 μg of BSA was added to each sample and incubated for 30 min at 4 °C. The beads were washed sequentially with Wash A (20 mM Tris-HCl, pH 8.0, 1 mM EDTA, 0.1% SDS, 1% Triton X-100, 150 mM NaCl), Wash B (20 mM Tris-HCl, pH 8.0, 1 mM EDTA, 0.1% SDS, 1% Triton X-100, 500 mM NaCl), Wash C (20 mM Tris-HCl, pH 8.0, 1 mM EDTA, 0.5% NP40, 0.5% sodium deoxycholate, 250 mM LiCl), and TE (10 mM Tris-HCl, pH 8.0, 1 mM EDTA) buffers. Chromatin was eluted in three 150 μL aliquots of Elution Buffer, and reverse cross-linking was performed by adding 200 mM NaCl and incubating the samples at 65 °C for 4 h. The antibodies used included anti-Histone 3, anti-Histone H3K9me3, anti-Histone H4, anti-acetyl-Histone H4, anti-TRIM28 or corresponding antibodies.

The eluted DNA was treated with 10 μL of 0.5 M EDTA, 20 μL of 1 M Tris-HCl (pH 6.5), and 5 μg of RNase A, followed by incubation at 37 °C for 1 h. Subsequently, 40 μg of Proteinase K was added, and the samples were incubated at 50 °C for 1 h. DNA was purified using a Qiagen PCR purification kit. The purified DNA (50 μL) was mixed with 50 μL of 0.46 M NaOH, heated at 95 °C for 5 min, and cooled on ice before neutralization.

\Dot blotting was performed on Hybond XL membranes (GE Healthcare Life Sciences) following the manufacturer’s protocol. Membranes were air-dried for 5 min, cross-linked with 240 mJ using a Stratalinker at 254 nm, and prehybridized in Church hybridization buffer (1% BSA, 1 mM EDTA, 500 mM NaHPO_4_ [pH 7.2], and 7% SDS). Hybridization was carried out overnight with a 5’-(CCCTAA)_3_-3’ DNA oligonucleotide probe end-labeled with [γ-32P] ATP using T4 polynucleotide kinase. The membranes were washed three times for 5 min in 2× SSC (0.3 M NaCl and 0.03 M sodium citrate), exposed to a phosphor screen (Amersham™ Typhoon™ laser scanner), and imaged. Signal quantification was performed using the array analysis function in FIJI, with background subtraction from unused wells. The amount of immunoprecipitated telomeric DNA was normalized for each sample based on input telomeric DNA signal intensities determined from standard curves.

### Immunoprecipitation (IP)

Cells cultured in 10 cm plates were prepared for immunoprecipitation as follows. The supernatant was removed, and the cells were washed with 1 mL of PBS. The cell suspension was transferred to an Eppendorf tube, centrifuged at 4 °C, and the supernatant was discarded. The pellet was washed twice with 1 mL of PBS, with centrifugation performed after each wash. The washed pellet was resuspended in 570 μL of NETN lysis buffer (1 mM EDTA, 40 mM Tris, 100 mM NaCl, 0.5% V/V NP-40) containing protease inhibitors, vortexed, and centrifuged at maximum speed for 10 min at 4 °C. For nucleic acid digestion, 3 mM MgCl₂ and benzonase can be added at this step, followed by incubation at 4 °C for 1 h. The resulting supernatant was transferred to a new tube, centrifuged again, and 930 μL of the cleared lysate was used for immunoprecipitation, with 25–50 μL reserved as an input control. For immunoprecipitation, 30 μL of anti-Flag agarose beads were added to each sample, and the mixture was incubated at 4 °C for 30 min with gentle rotation. The beads were washed with NETN buffer and resuspended in 70 μL of 2× SDS loading buffer containing 5% β-mercaptoethanol. The samples were incubated at room temperature for 10 min, centrifuged, and the supernatant containing the immunoprecipitated proteins was collected for western blot analysis.

### Image acquisition and quantification

Fluorescence 3D images were acquired using a GE Healthcare DeltaVision Deconvolution microscope with 0.2 μm spacing for a total of 5 μm. The images were processed with software version 5.5.1 for deconvolution and analyzed using ImageJ/FIJI for quantification. Initially, a maximum intensity projection was applied to convert the Z-stack into a 2D image. Background subtraction was performed using the ‘tophat’ filter. To identify foci, we applied Ilastik, a tool trained with an artificial intelligence (AI) model (Berg et al, 2019).

For TRIM28 and telomere colocalization analysis and representative images, a single optical section from the middle of the Z-stack was used to minimize false overlap introduced by projection, whereas maximal intensity Z-projections were used for quantitative analyses.

### Statistical analyses

Data visualization, including tables and graphs, was performed using GraphPad Prism 9 and Microsoft Excel. For imaging-based cell analysis, each experiment was independently repeated three times, with a minimum of 50 cells analyzed per replicate, yielding a total of approximately 150–300 cells per experimental condition. In scatter plots, each point represents a quantified value per cell, with red lines denoting either the mean or median for inter-sample comparison. Statistical analyses were conducted using two-tailed Mann–Whitney *U* tests or unpaired *t* tests, depending on the data distribution and experimental design.

## Supplementary information

Source data Fig. 1

Source data Fig. 2

Source data Fig. 3

Source data Fig. 4

Source data Fig. 5

Figure EV1 Source Data

Figure EV2 Source Data

Figure EV3 Source Data

Figure EV4 Source Data

Figure EV5 Source Data

Figure EFV6 Source Data

Expanded View Figures

## Data Availability

The bioimaging data from this publication have been deposited to the BioImage Archive database (https://www.ebi.ac.uk/bioimage-archive/) and assigned the BioImages accession number S-BIAD2934. The source data of this paper are collected in the following database record: biostudies:S-SCDT-10_1038-S44318-026-00760-w.
