# Supplementary figures and images for: Orphan nuclear receptors recruit TRIM28 to promote telomeric H3K9me3 for the ALT pathway
Source: EMBO J. 2026 Mar 31;45(10):3444–70. doi: 10.1038/s44318-026-00760-w (PMC13187458; doi:10.1038/s44318-026-00760-w)

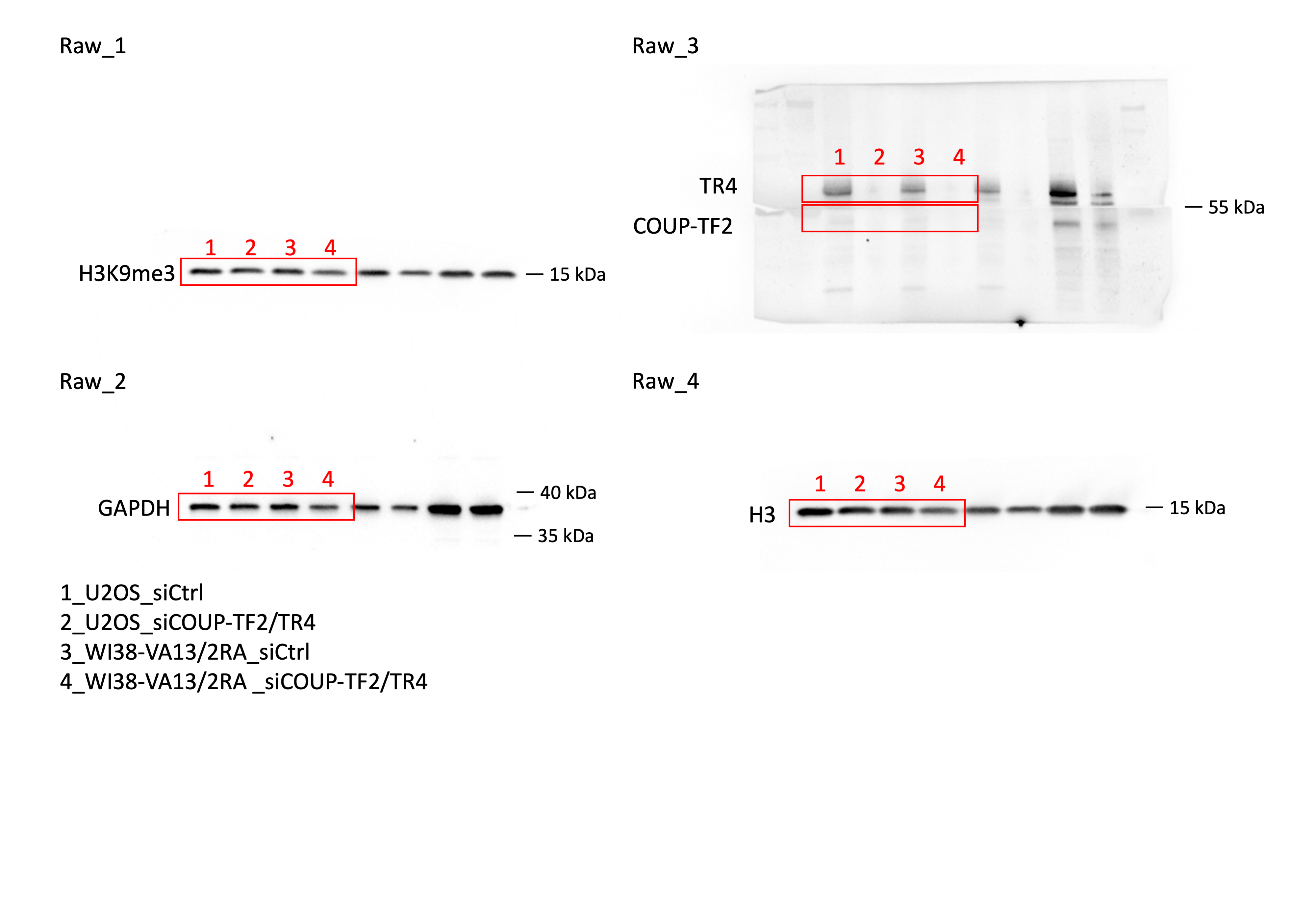

Supplement: Supplementary file 1 — Source data Fig. 1 [file 44318_2026_760_MOESM1_ESM.zip › Figure 1/A/RawBlot_annotated.tiff]

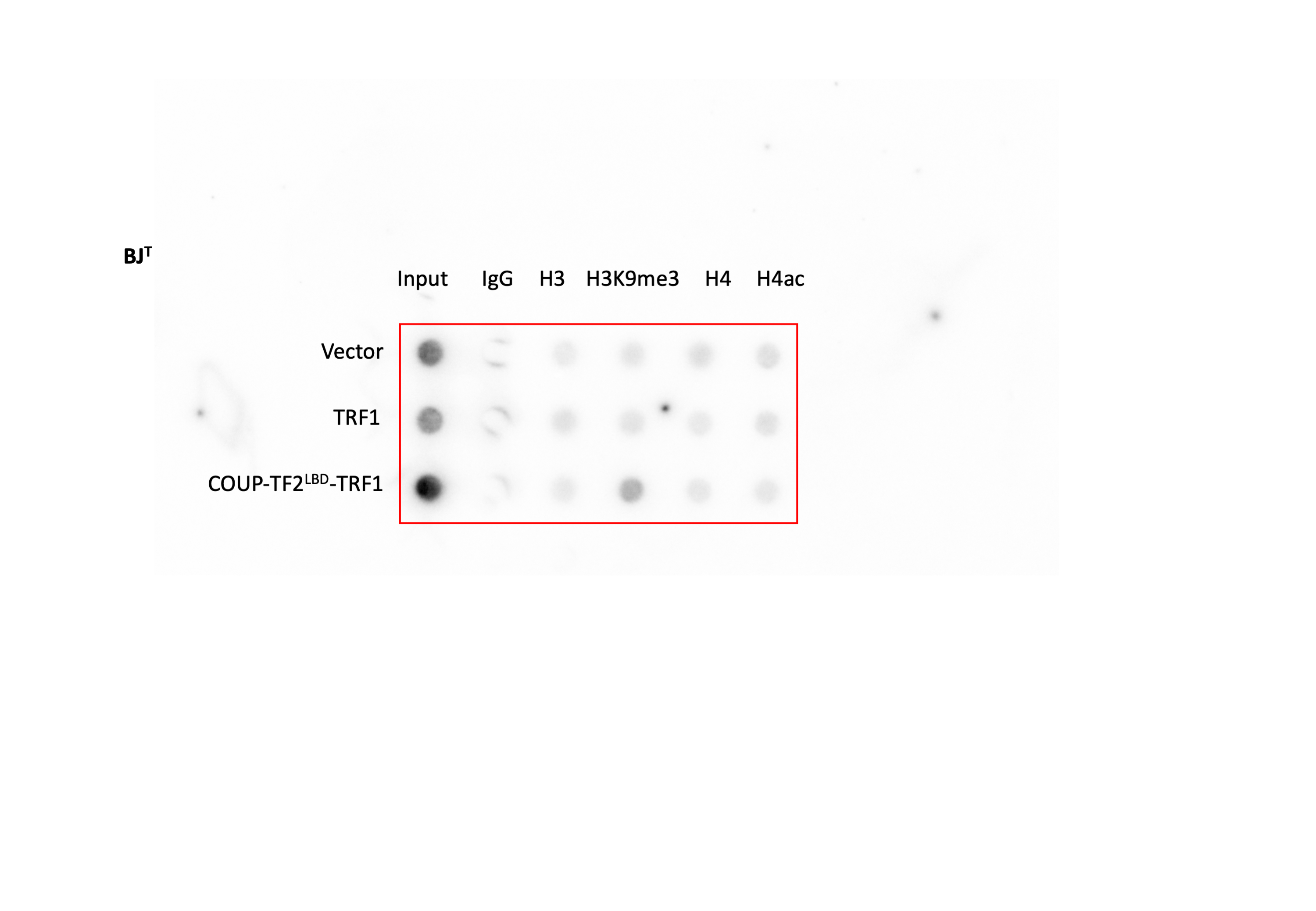

Supplement: Supplementary file 1 — Source data Fig. 1 [file 44318_2026_760_MOESM1_ESM.zip › Figure 1/F/RawBlot_annotated.tiff]

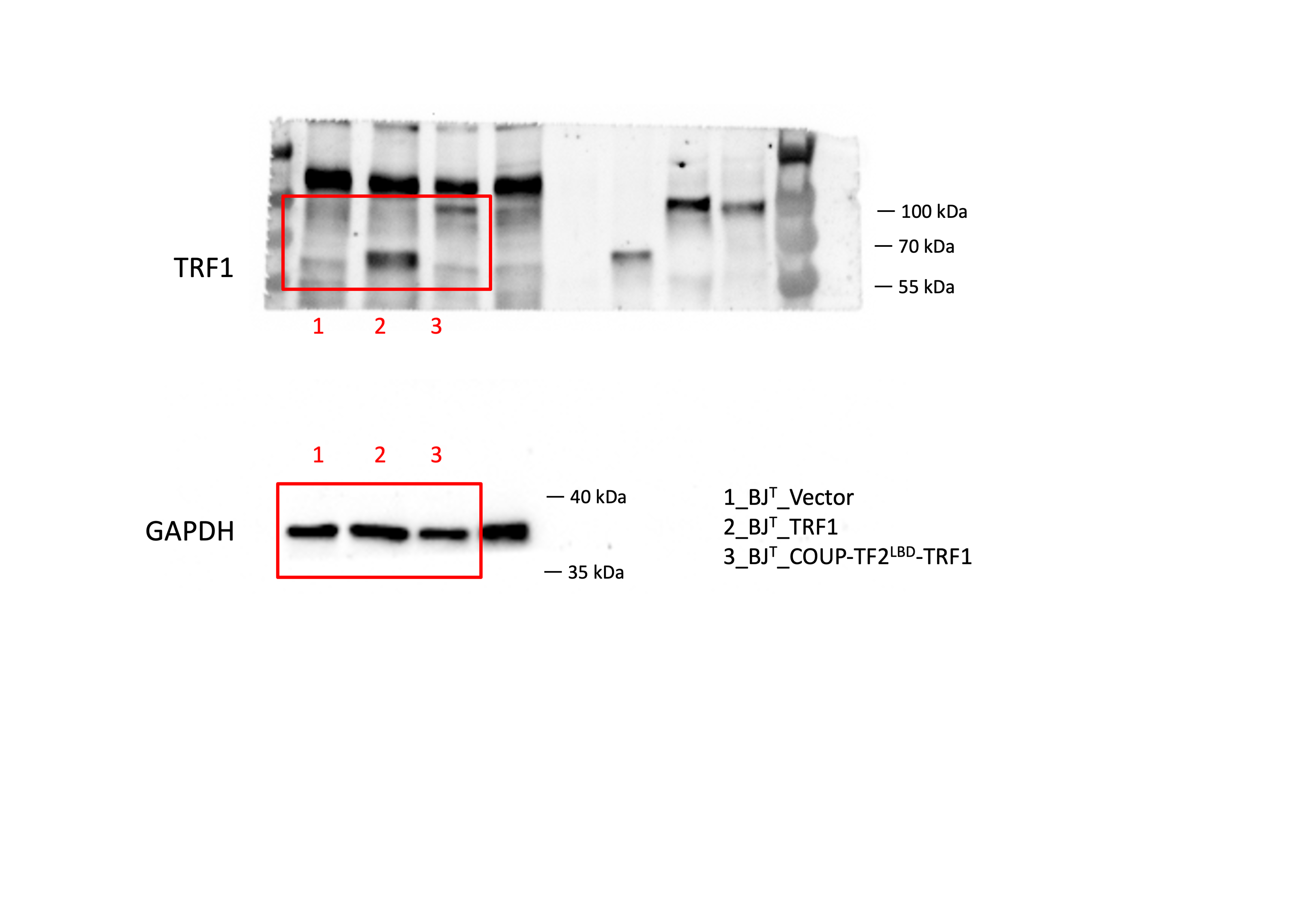

Supplement: Supplementary file 1 — Source data Fig. 1 [file 44318_2026_760_MOESM1_ESM.zip › Figure 1/C/RawBlot_annotated.tiff]

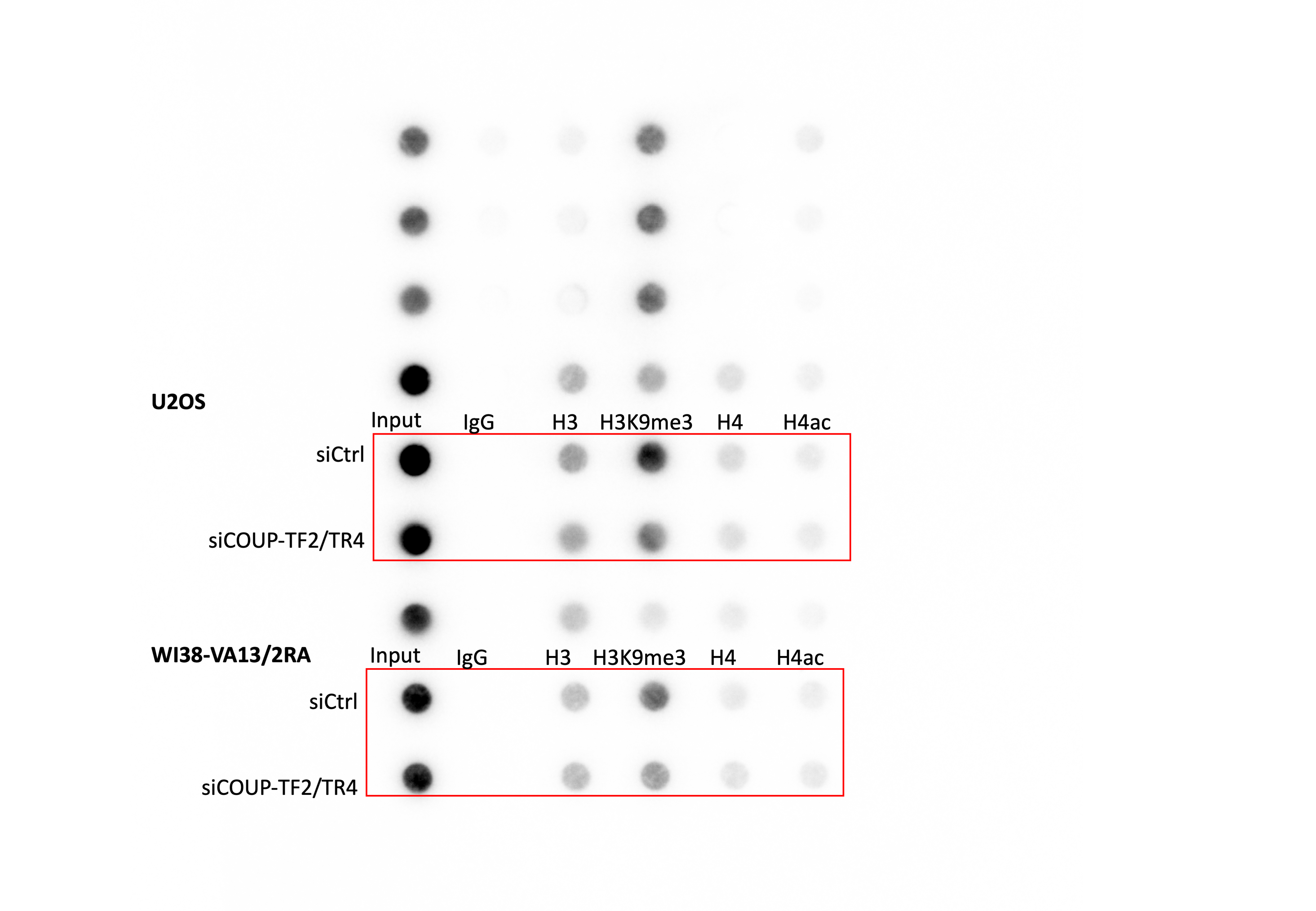

Supplement: Supplementary file 1 — Source data Fig. 1 [file 44318_2026_760_MOESM1_ESM.zip › Figure 1/B/Raw_dotblot_annotated.tiff]

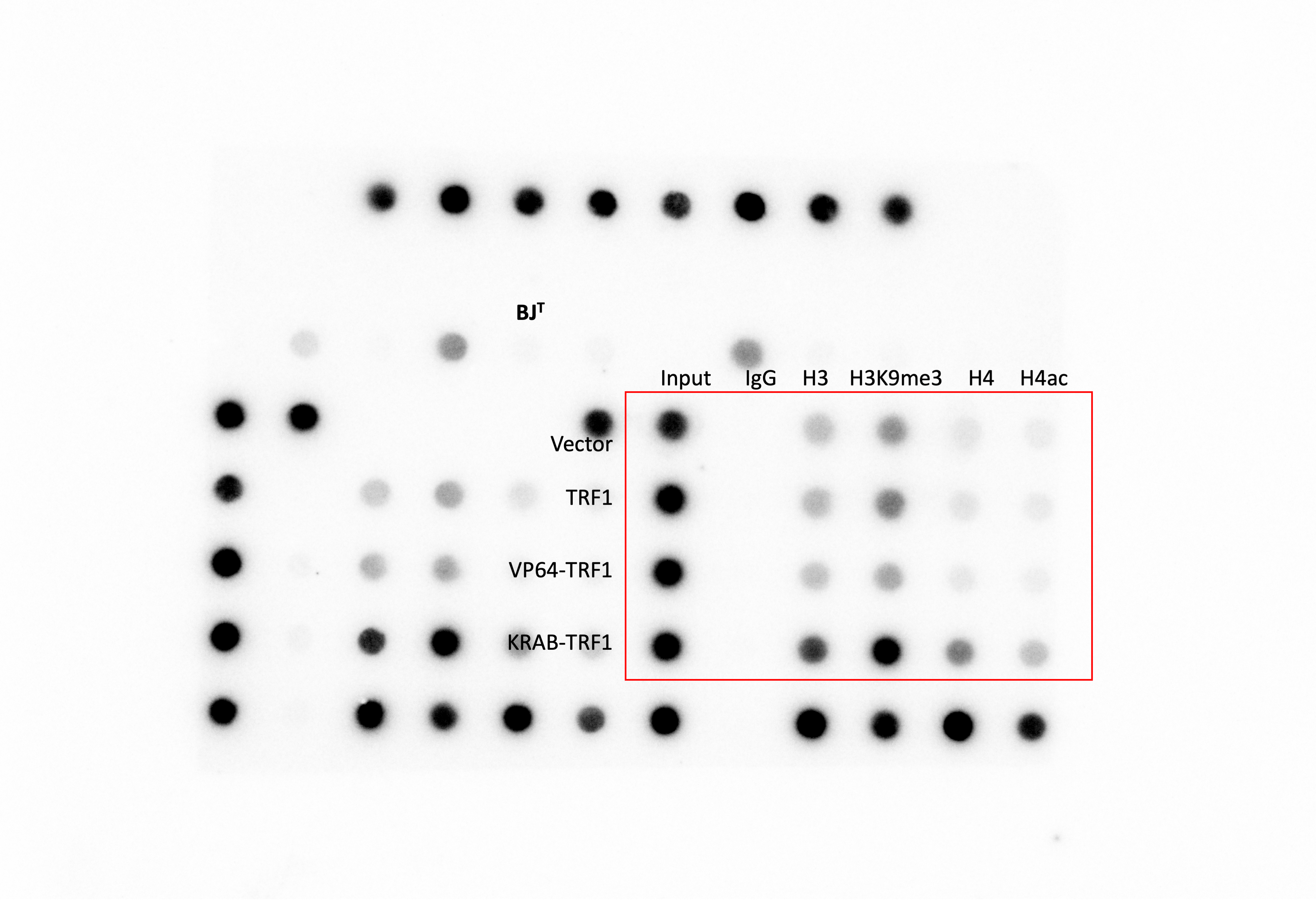

Supplement: Supplementary file 2 — Source data Fig. 2 [file 44318_2026_760_MOESM2_ESM.zip › Figure 2/B/Raw_dotblot_annotated.tiff]

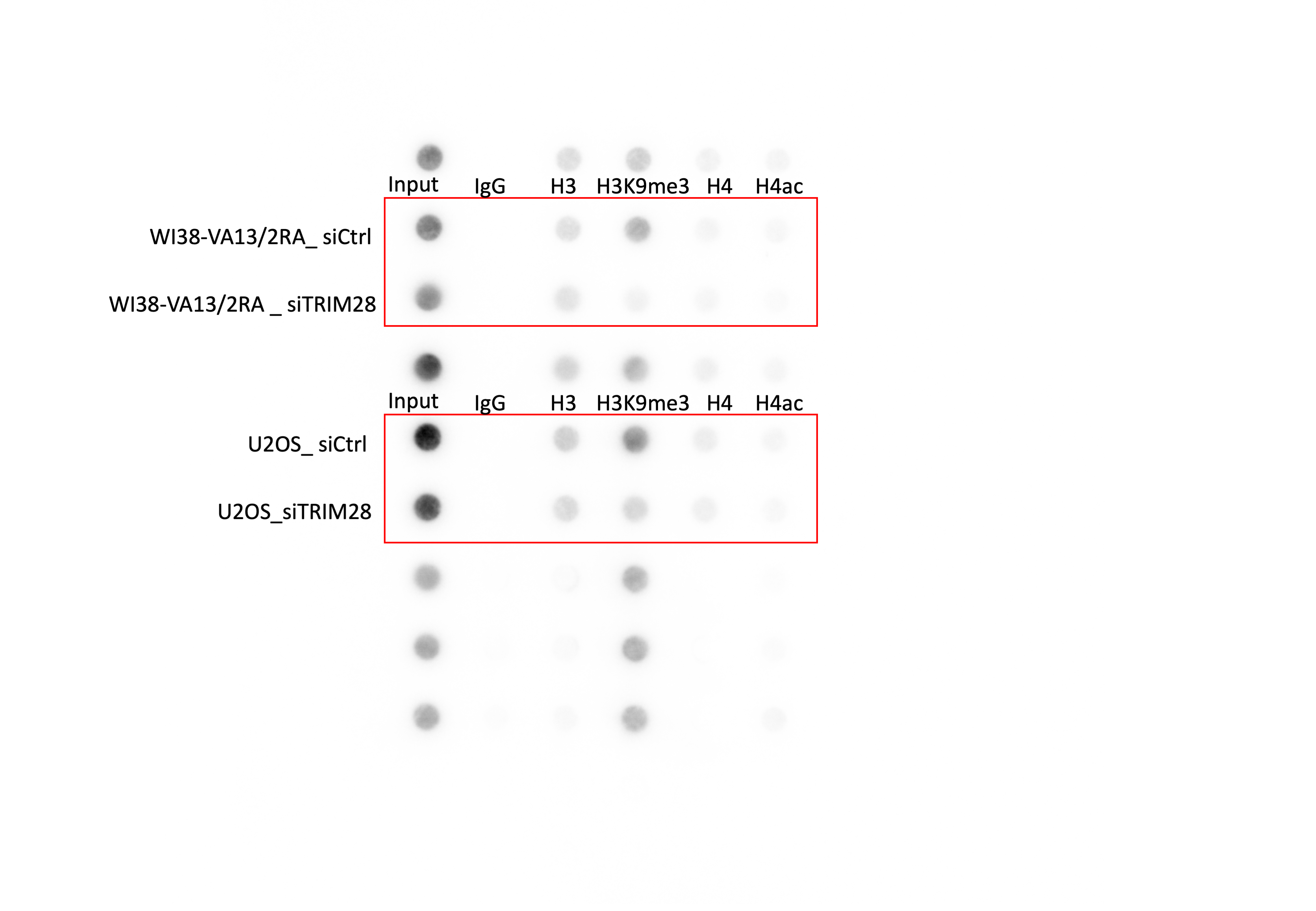

Supplement: Supplementary file 3 — Source data Fig. 3 [file 44318_2026_760_MOESM3_ESM.zip › Figure 3/G/Raw_dotblot_annotated.tiff]

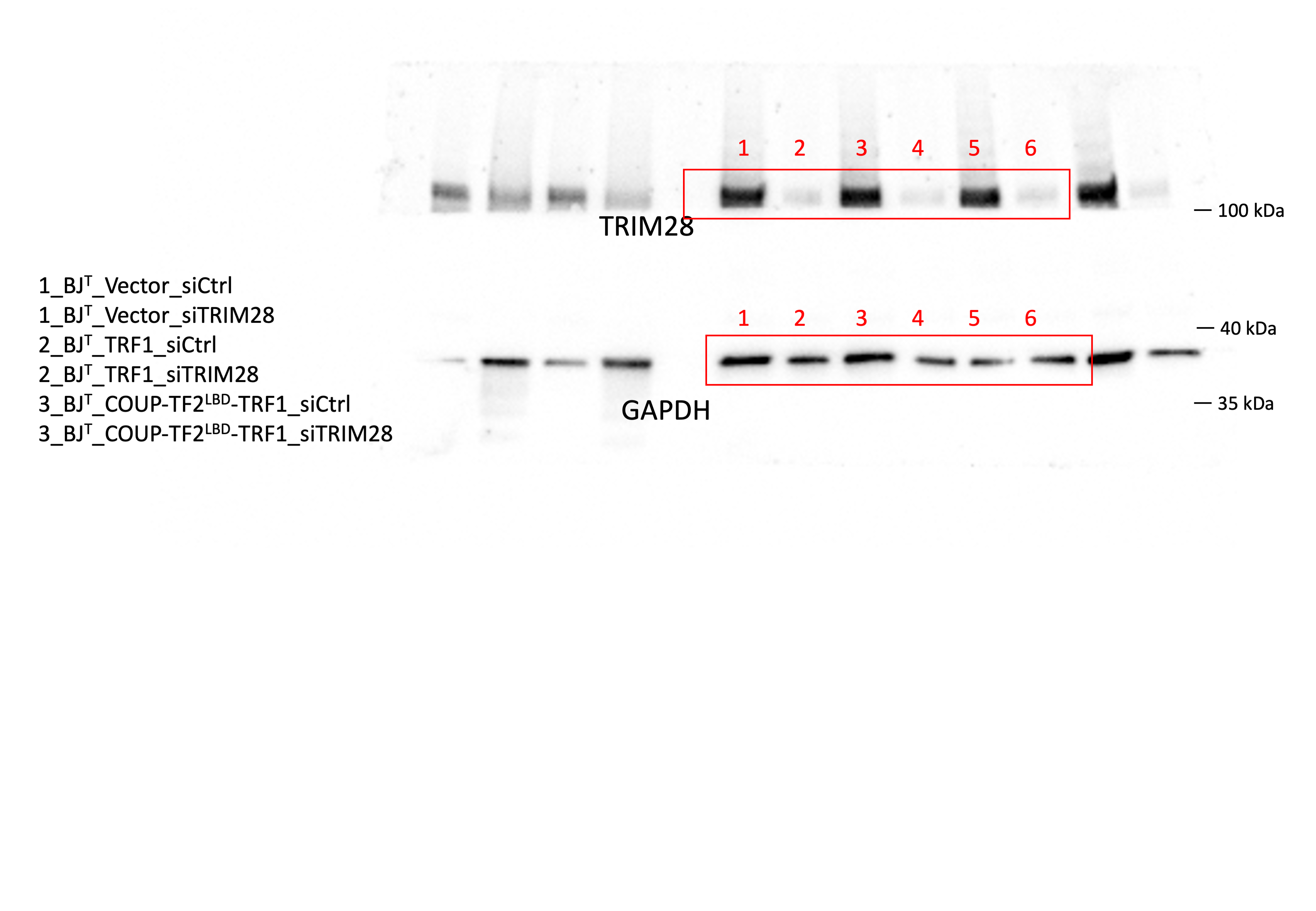

Supplement: Supplementary file 3 — Source data Fig. 3 [file 44318_2026_760_MOESM3_ESM.zip › Figure 3/A/RawBlot_annotated.tiff]

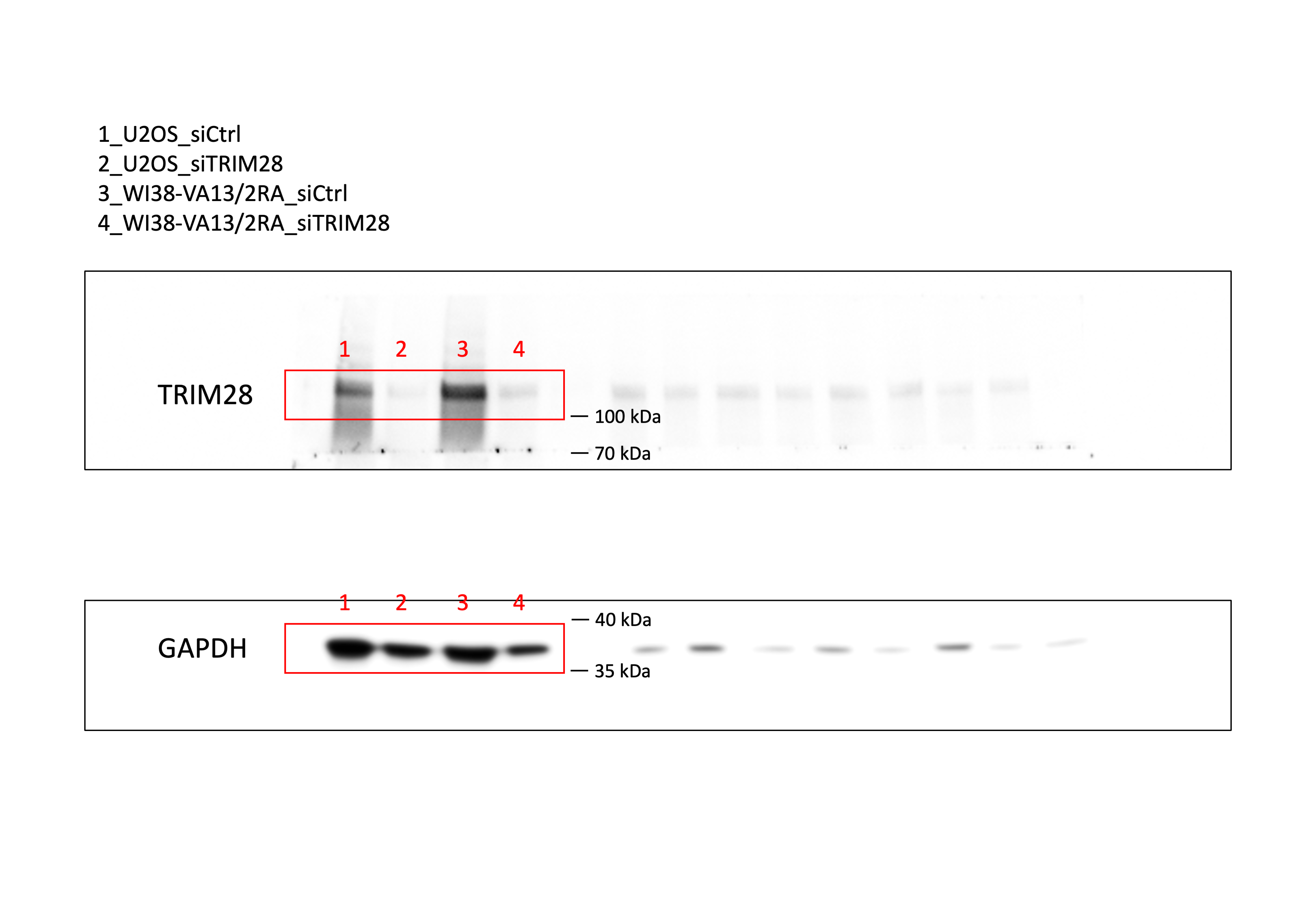

Supplement: Supplementary file 3 — Source data Fig. 3 [file 44318_2026_760_MOESM3_ESM.zip › Figure 3/F/RawBlot_annotated.tiff]

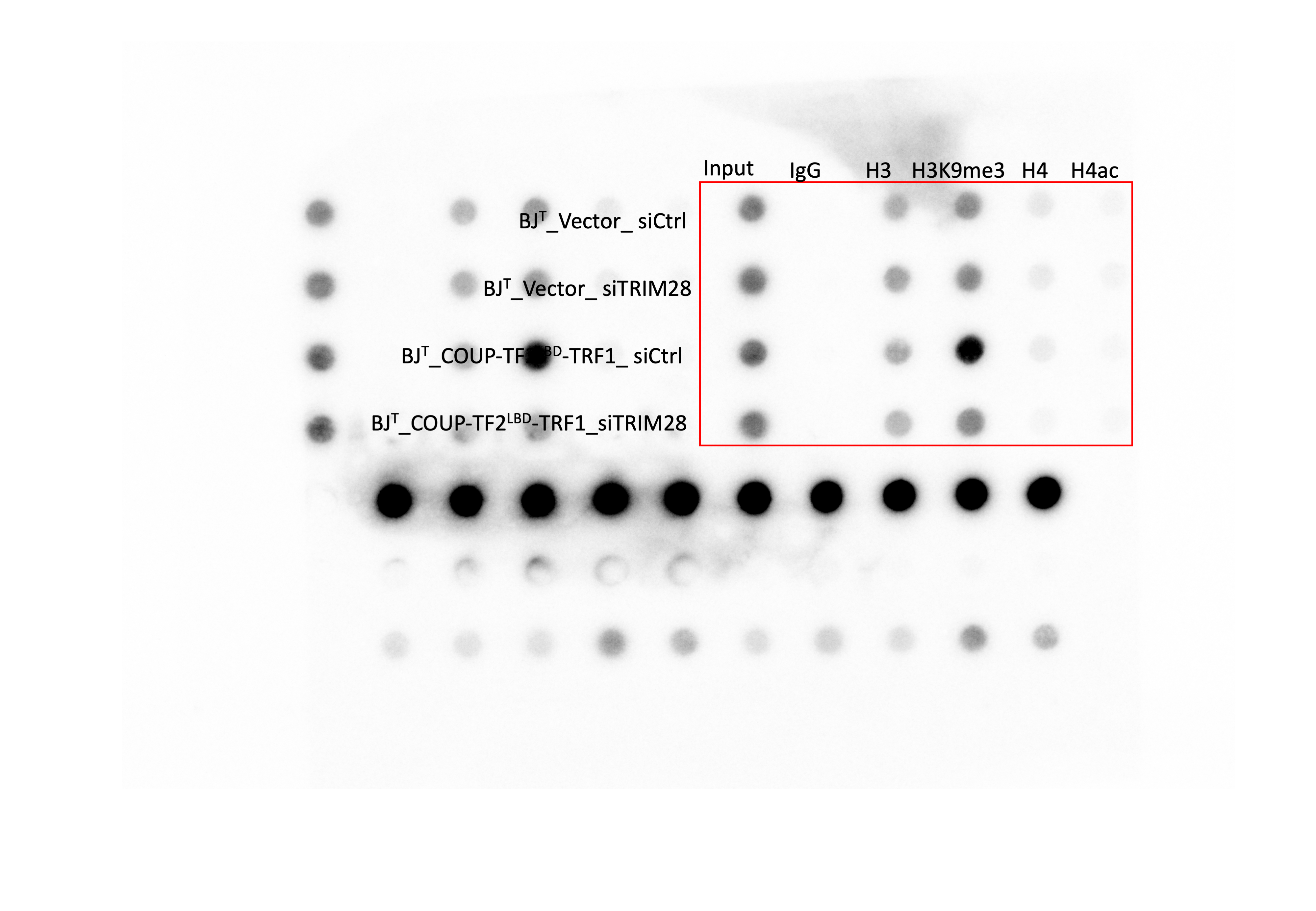

Supplement: Supplementary file 3 — Source data Fig. 3 [file 44318_2026_760_MOESM3_ESM.zip › Figure 3/B/Raw_dotblot_annotated.tiff]

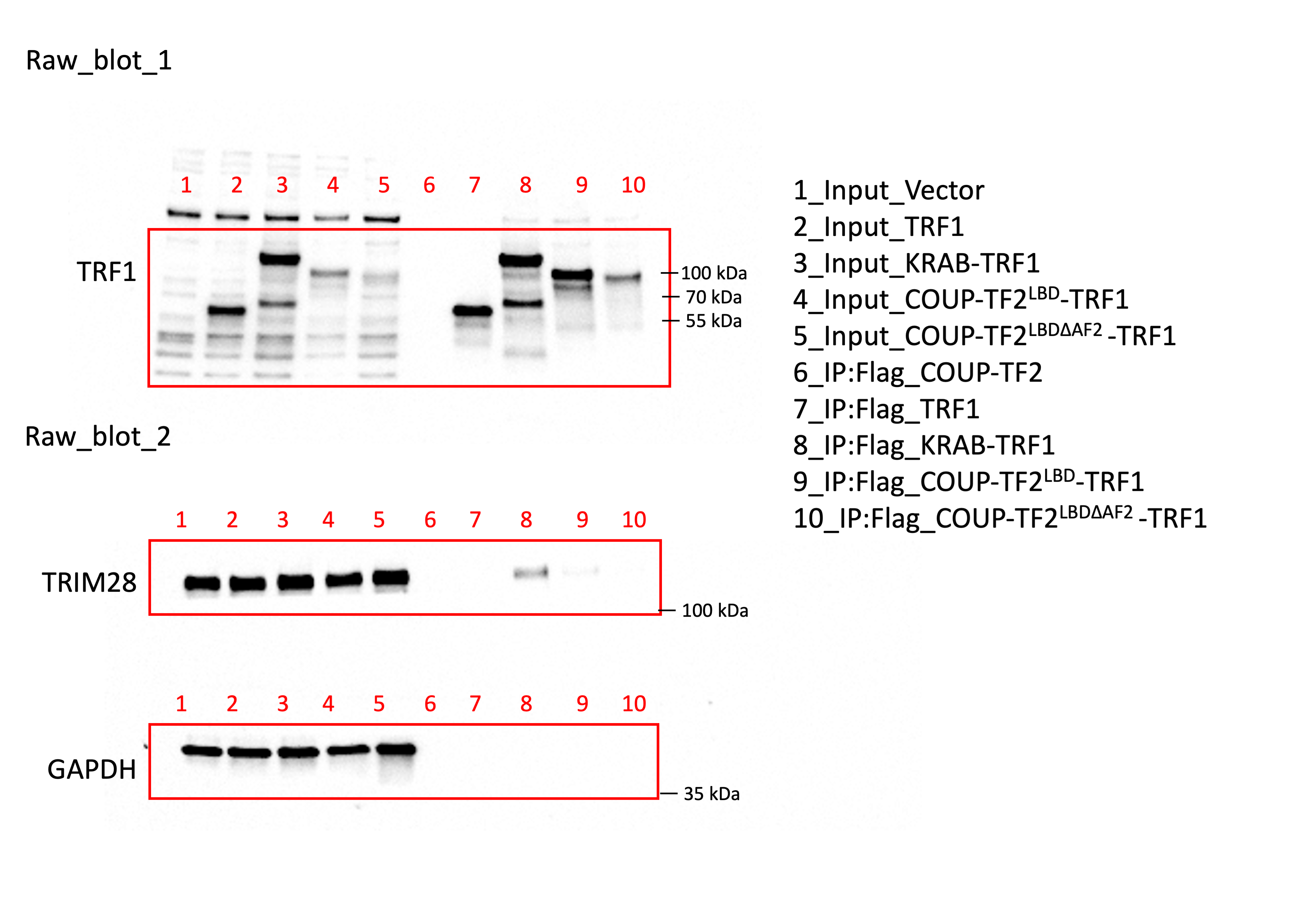

Supplement: Supplementary file 4 — Source data Fig. 4 [file 44318_2026_760_MOESM4_ESM.zip › Figure 4/G/RawBlot_annotated.tiff]

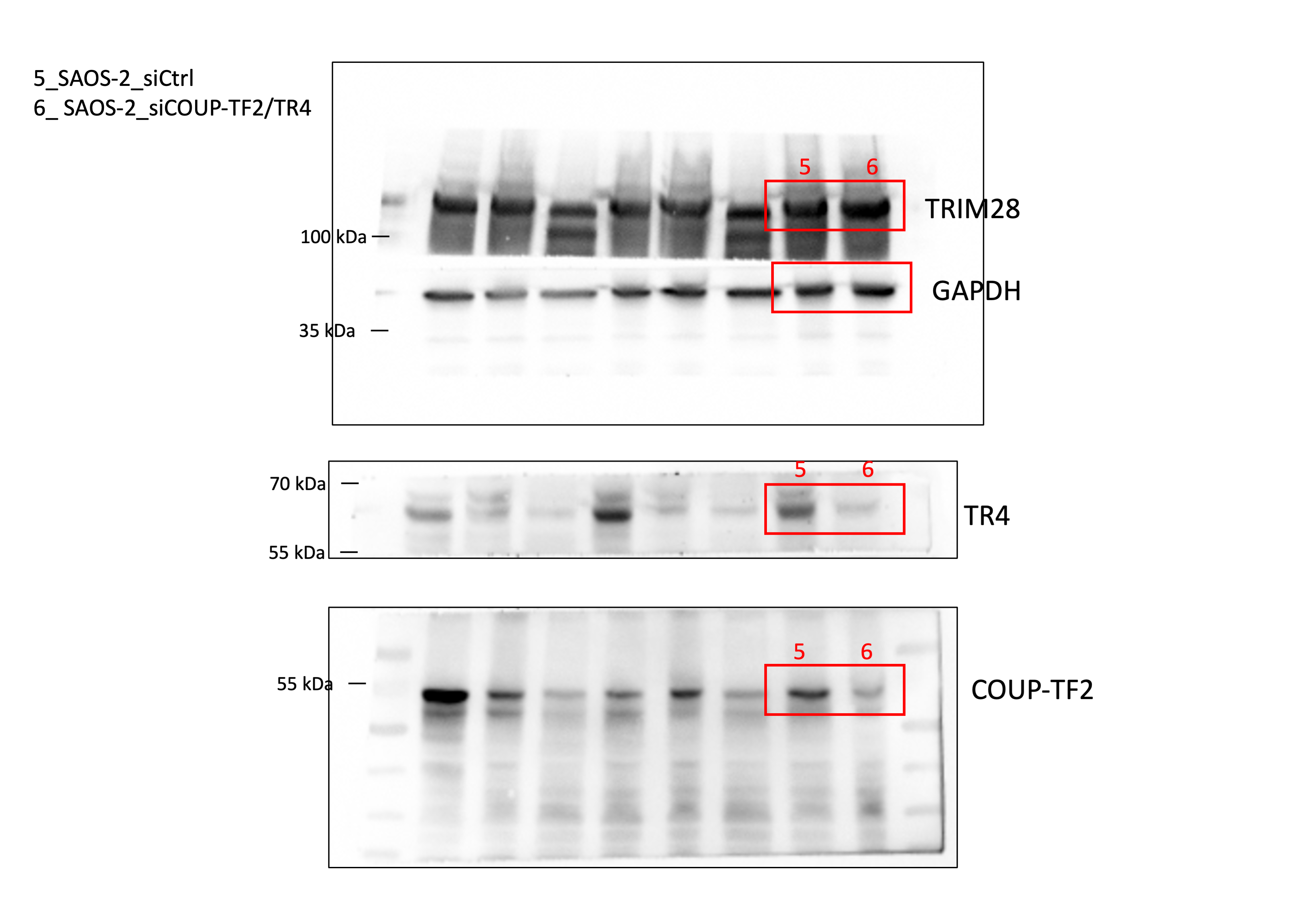

Supplement: Supplementary file 4 — Source data Fig. 4 [file 44318_2026_760_MOESM4_ESM.zip › Figure 4/A/Raw2_blot_annotated.tiff]

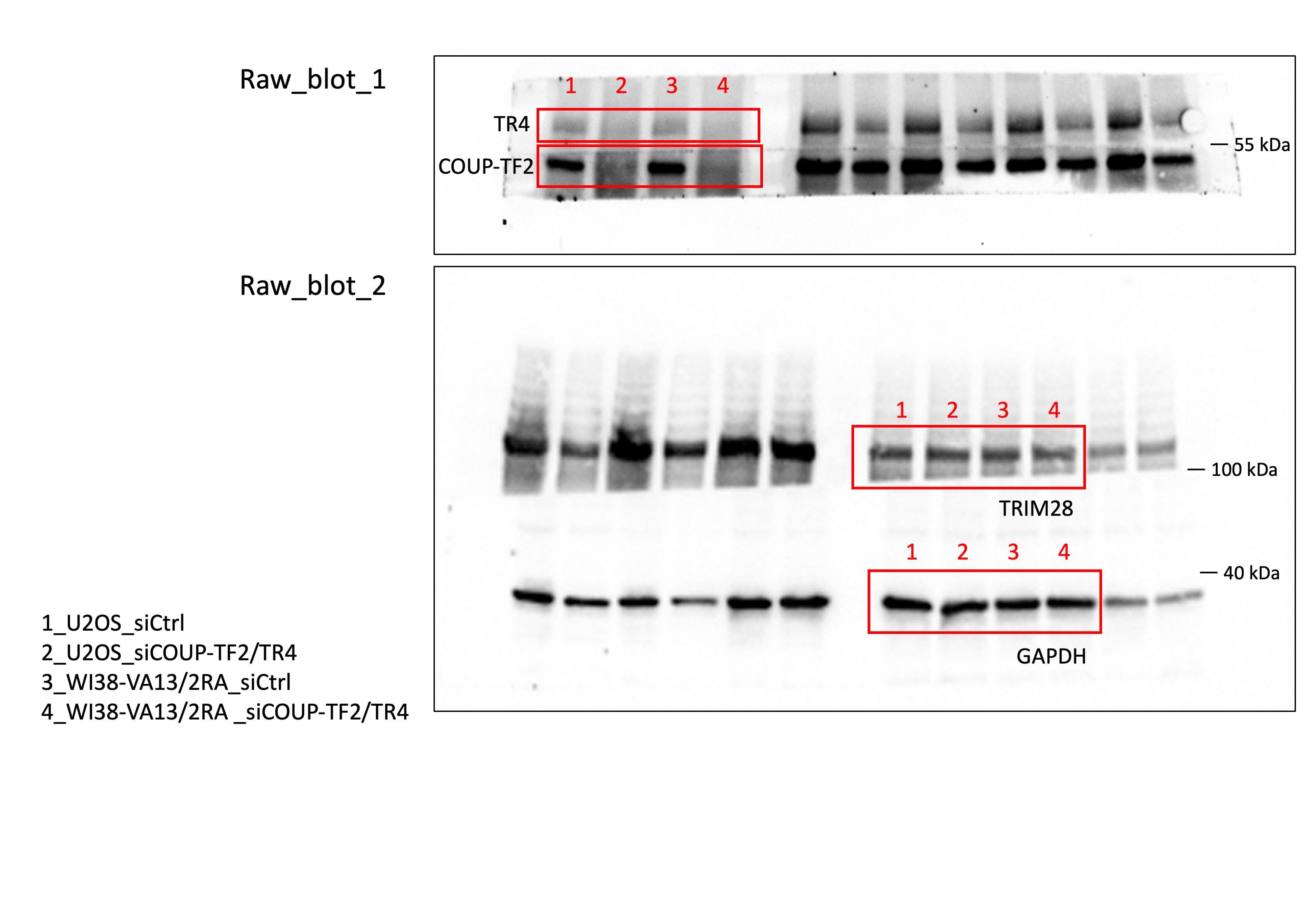

Supplement: Supplementary file 4 — Source data Fig. 4 [file 44318_2026_760_MOESM4_ESM.zip › Figure 4/A/Raw1_blot_annotated.tiff]

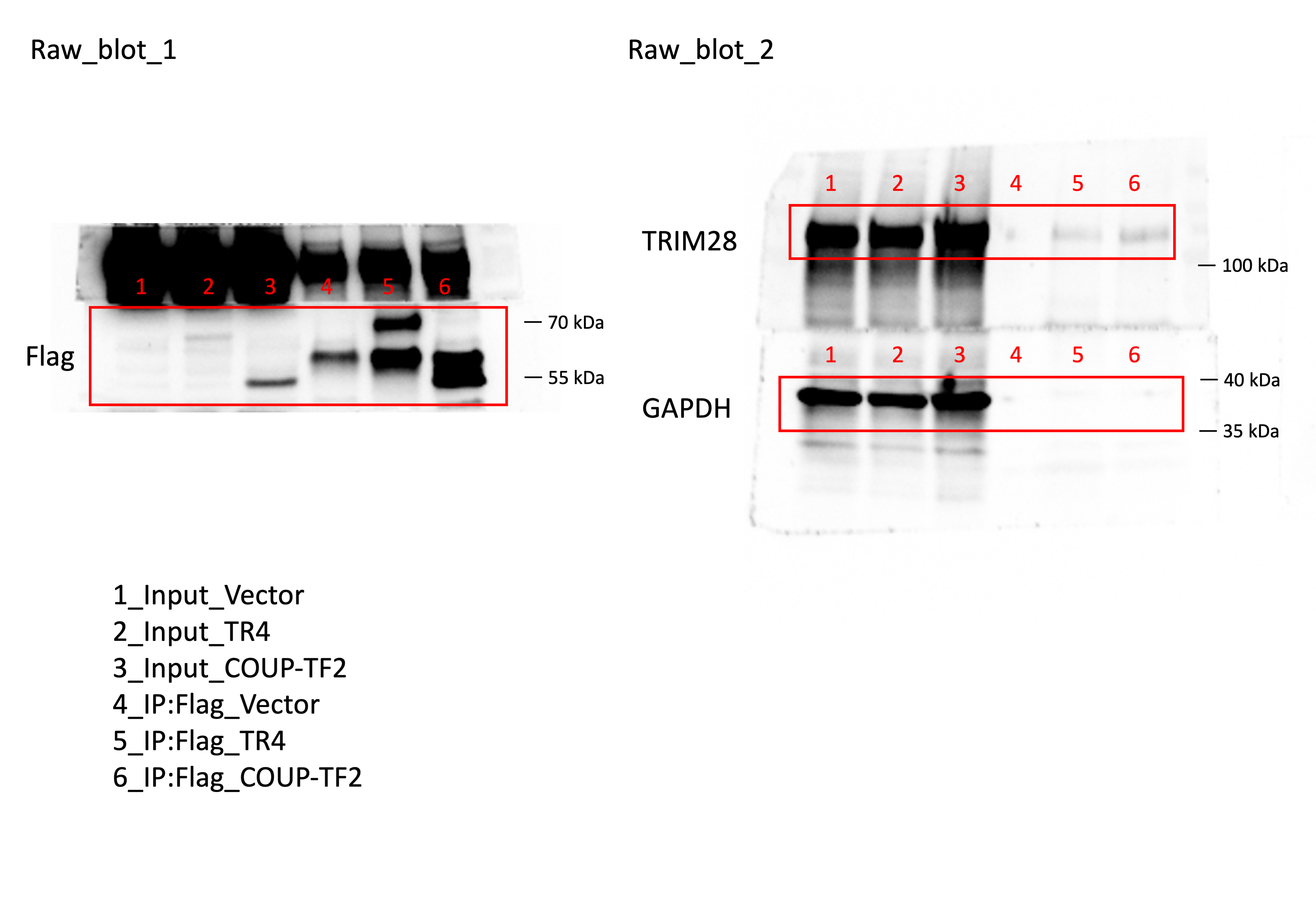

Supplement: Supplementary file 4 — Source data Fig. 4 [file 44318_2026_760_MOESM4_ESM.zip › Figure 4/F/RawBlot_annotated.tiff]

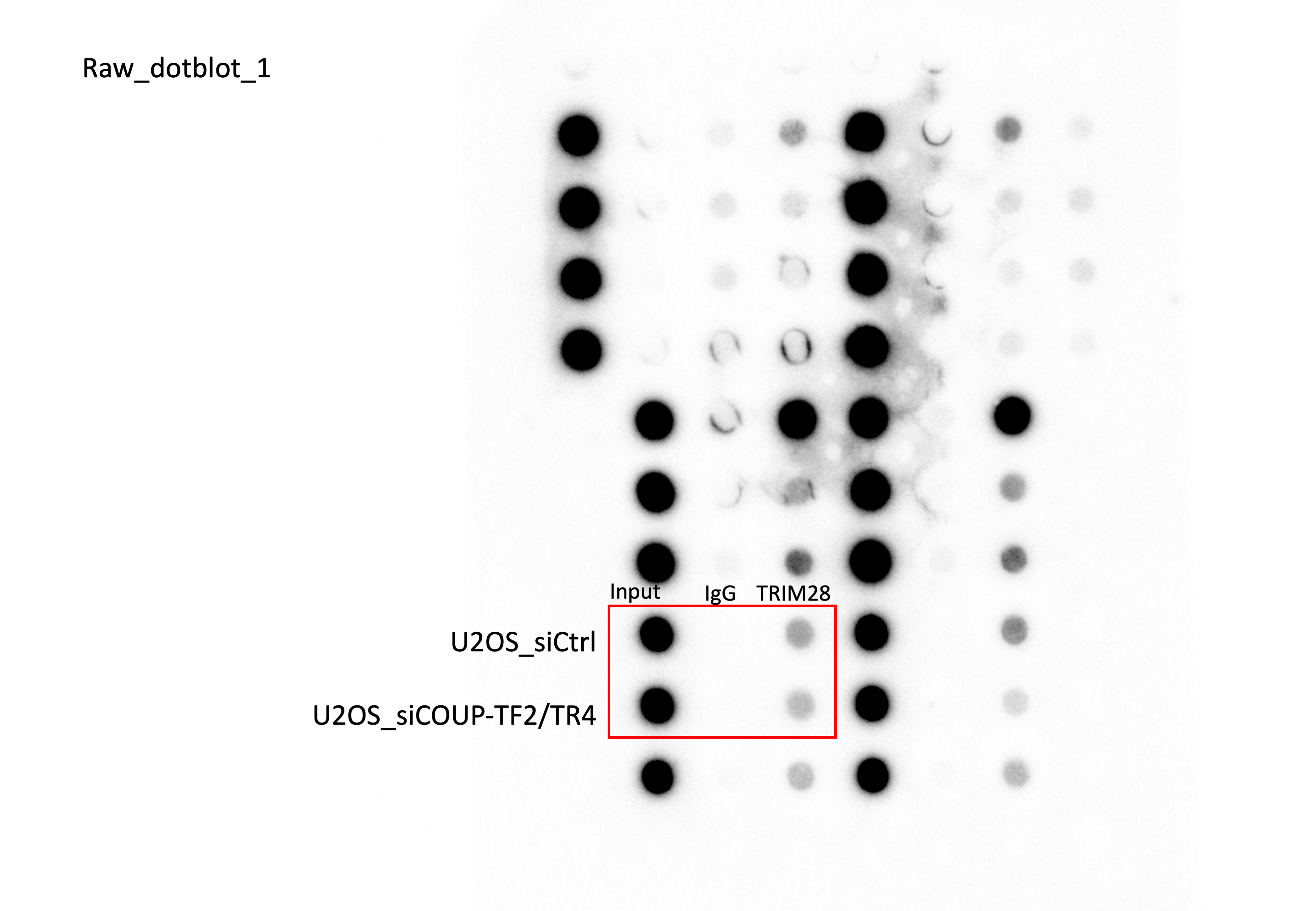

Supplement: Supplementary file 4 — Source data Fig. 4 [file 44318_2026_760_MOESM4_ESM.zip › Figure 4/B/Raw_dotblot_annotated_1.tiff]

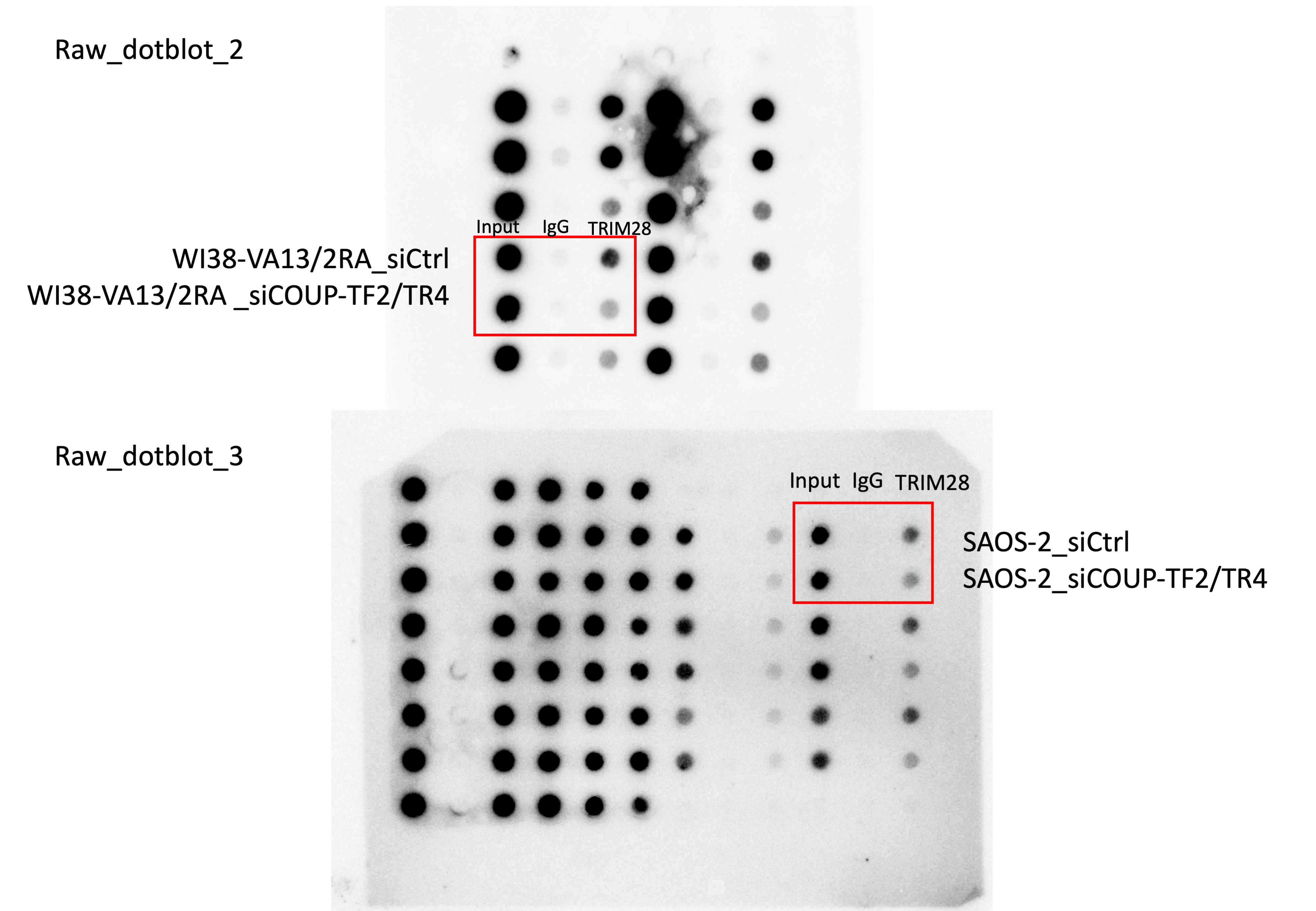

Supplement: Supplementary file 4 — Source data Fig. 4 [file 44318_2026_760_MOESM4_ESM.zip › Figure 4/B/Raw_dotblot_annotated_2.tiff]

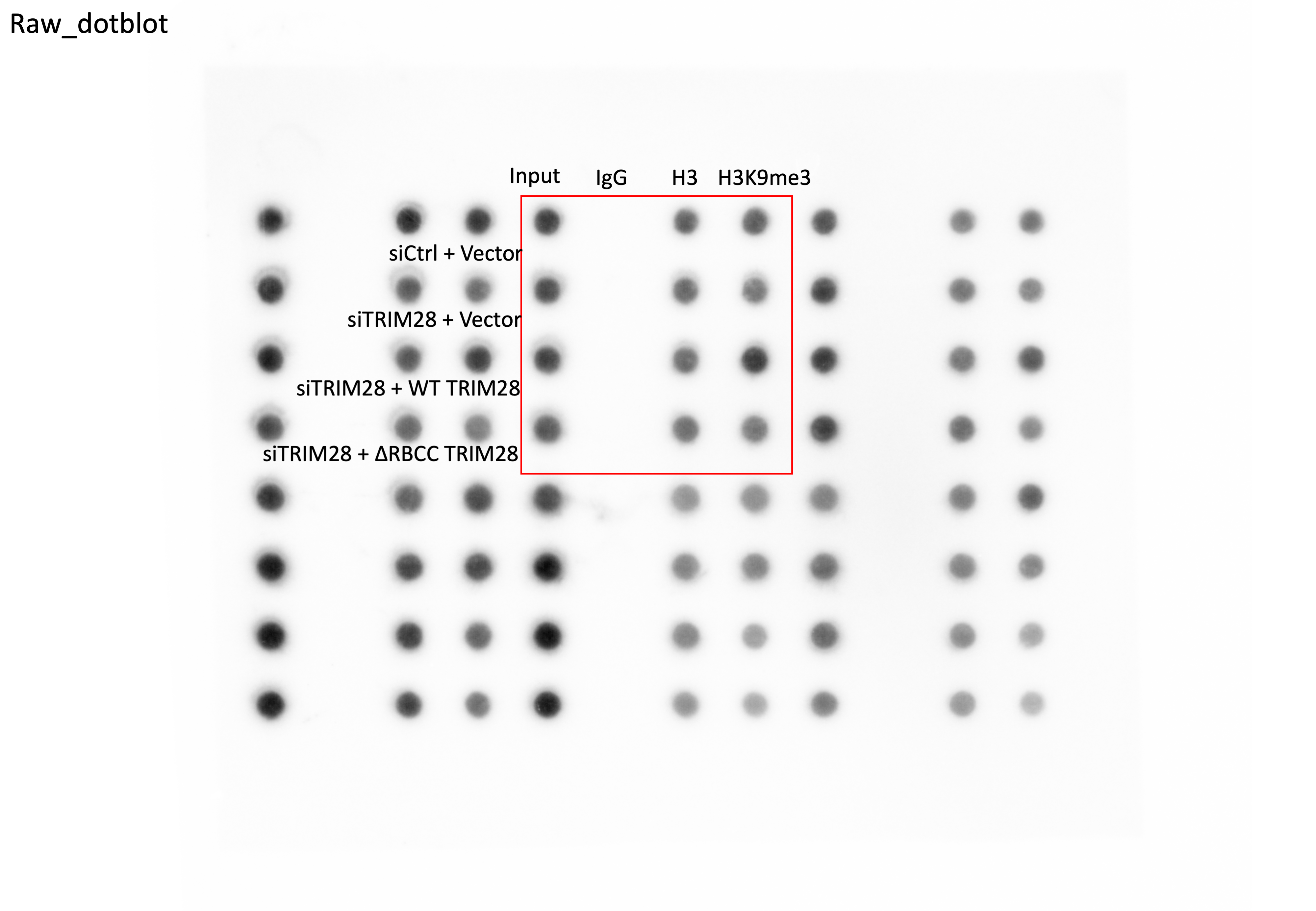

Supplement: Supplementary file 5 — Source data Fig. 5 [file 44318_2026_760_MOESM5_ESM.zip › Figure 5/H/Raw_dotblot_annotated.tiff]

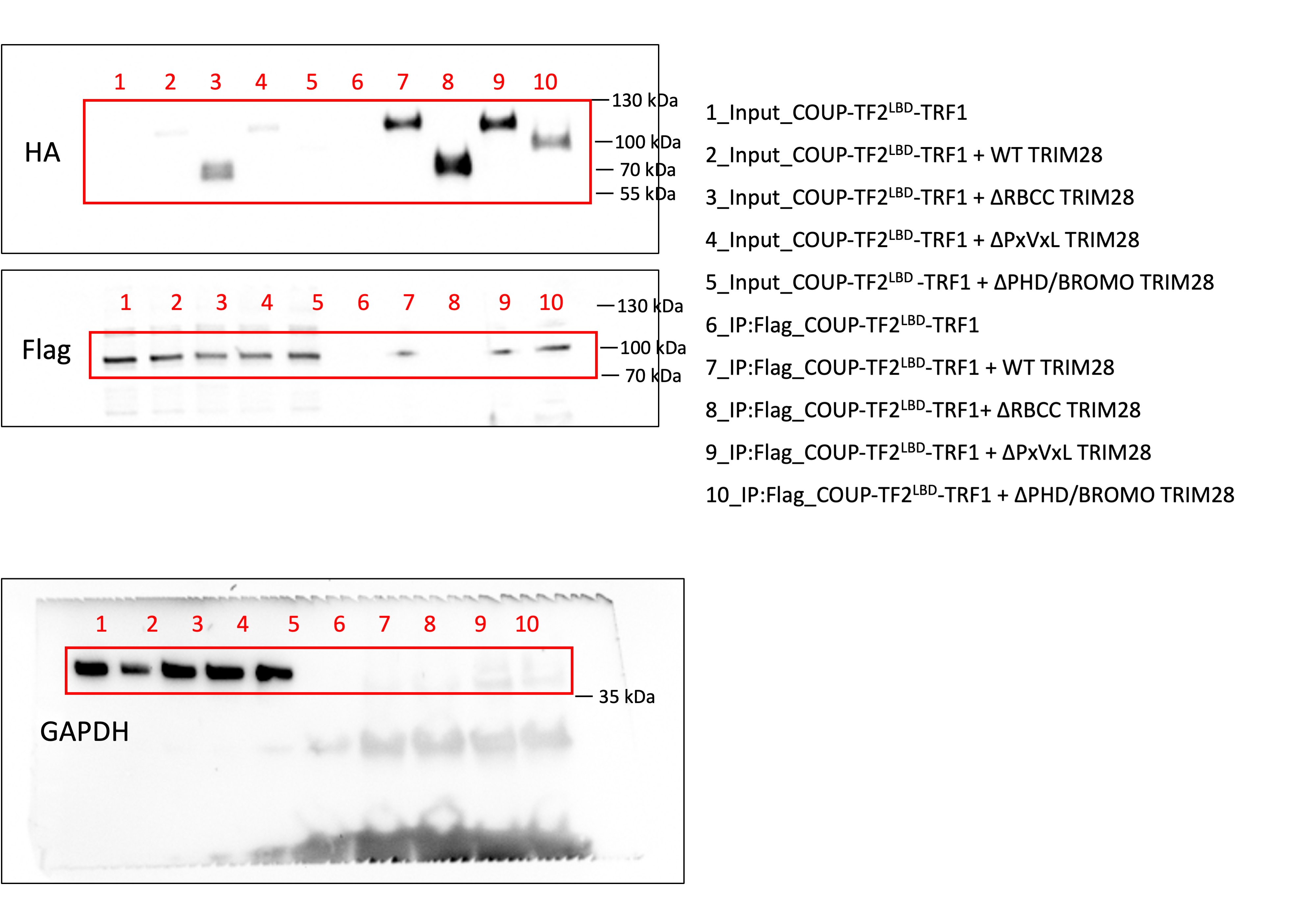

Supplement: Supplementary file 5 — Source data Fig. 5 [file 44318_2026_760_MOESM5_ESM.zip › Figure 5/D/RawBlot_annotated.tiff]

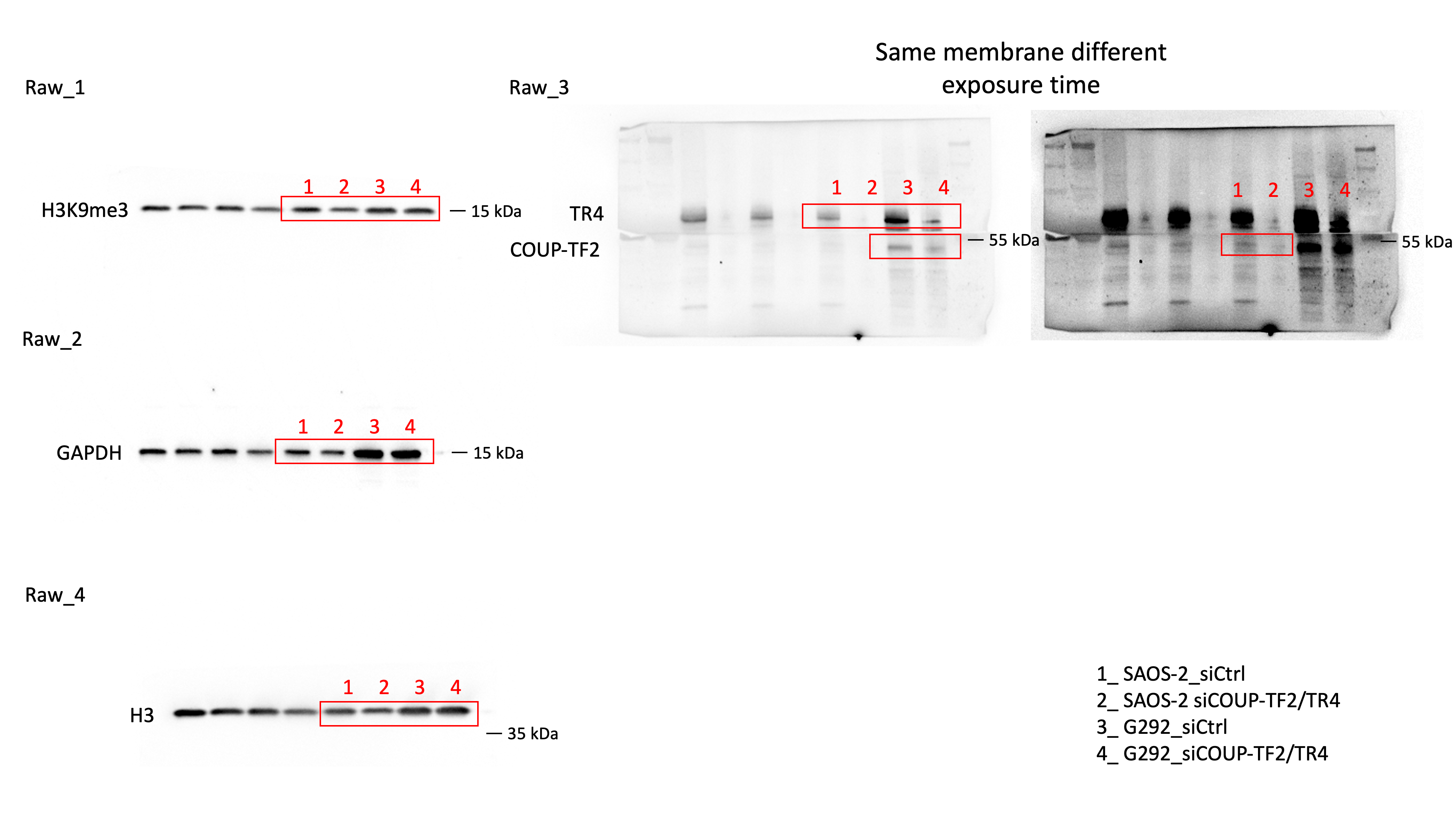

Supplement: Supplementary file 6 — Figure EV1 Source Data [file 44318_2026_760_MOESM6_ESM.zip › Figure EV1/A/RawBlot_annotated_Supplymentary.tiff]

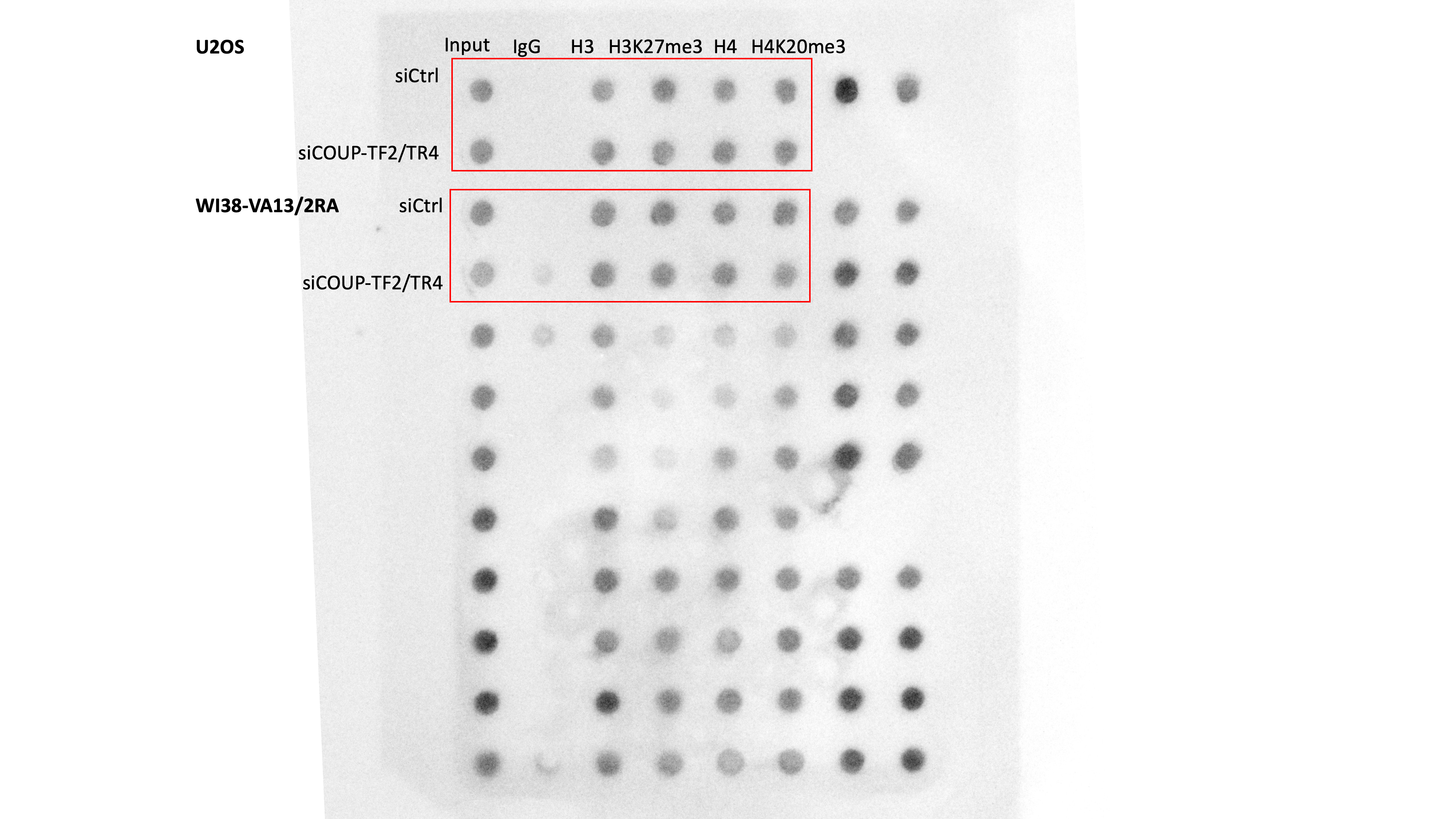

Supplement: Supplementary file 6 — Figure EV1 Source Data [file 44318_2026_760_MOESM6_ESM.zip › Figure EV1/C/RAW_dotblot_annotated_Supplymentary.tiff]

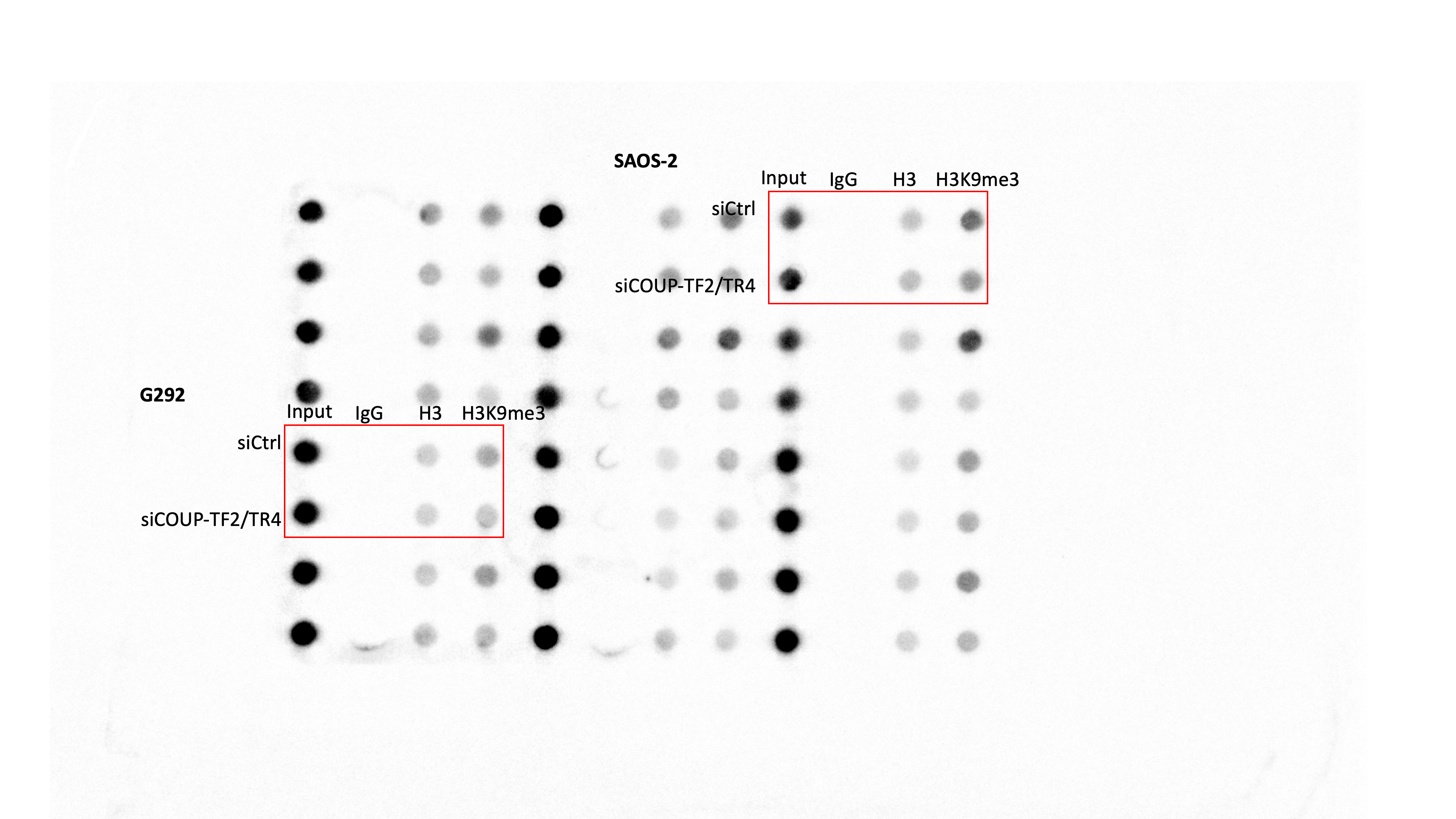

Supplement: Supplementary file 6 — Figure EV1 Source Data [file 44318_2026_760_MOESM6_ESM.zip › Figure EV1/B/RAW_dotblot_annotated_Supplymentary.tiff]

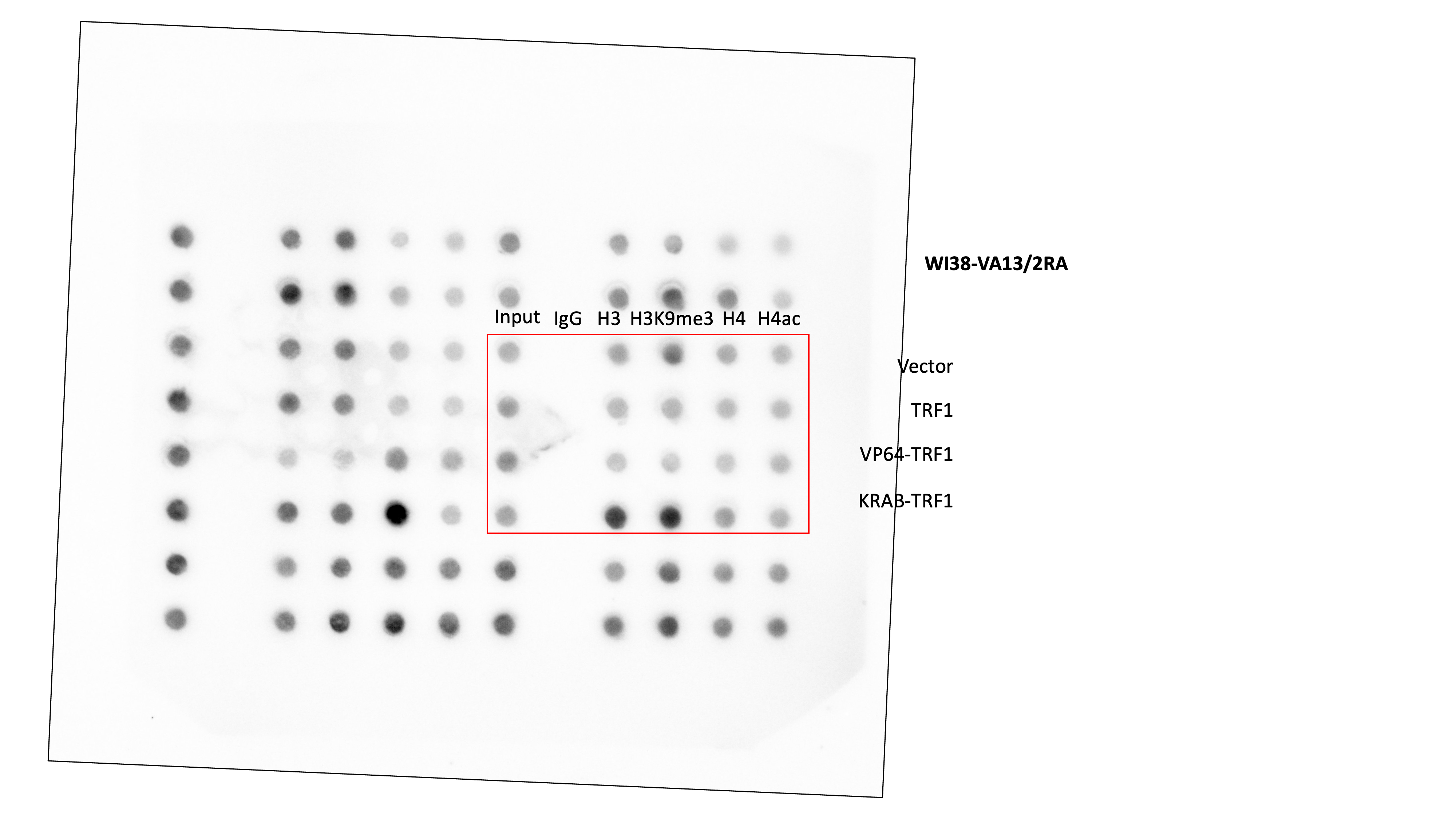

Supplement: Supplementary file 7 — Figure EV2 Source Data [file 44318_2026_760_MOESM7_ESM.zip › Figure EV2/C-E/Raw2_dotblot_annotated_Supplymentary.tiff]

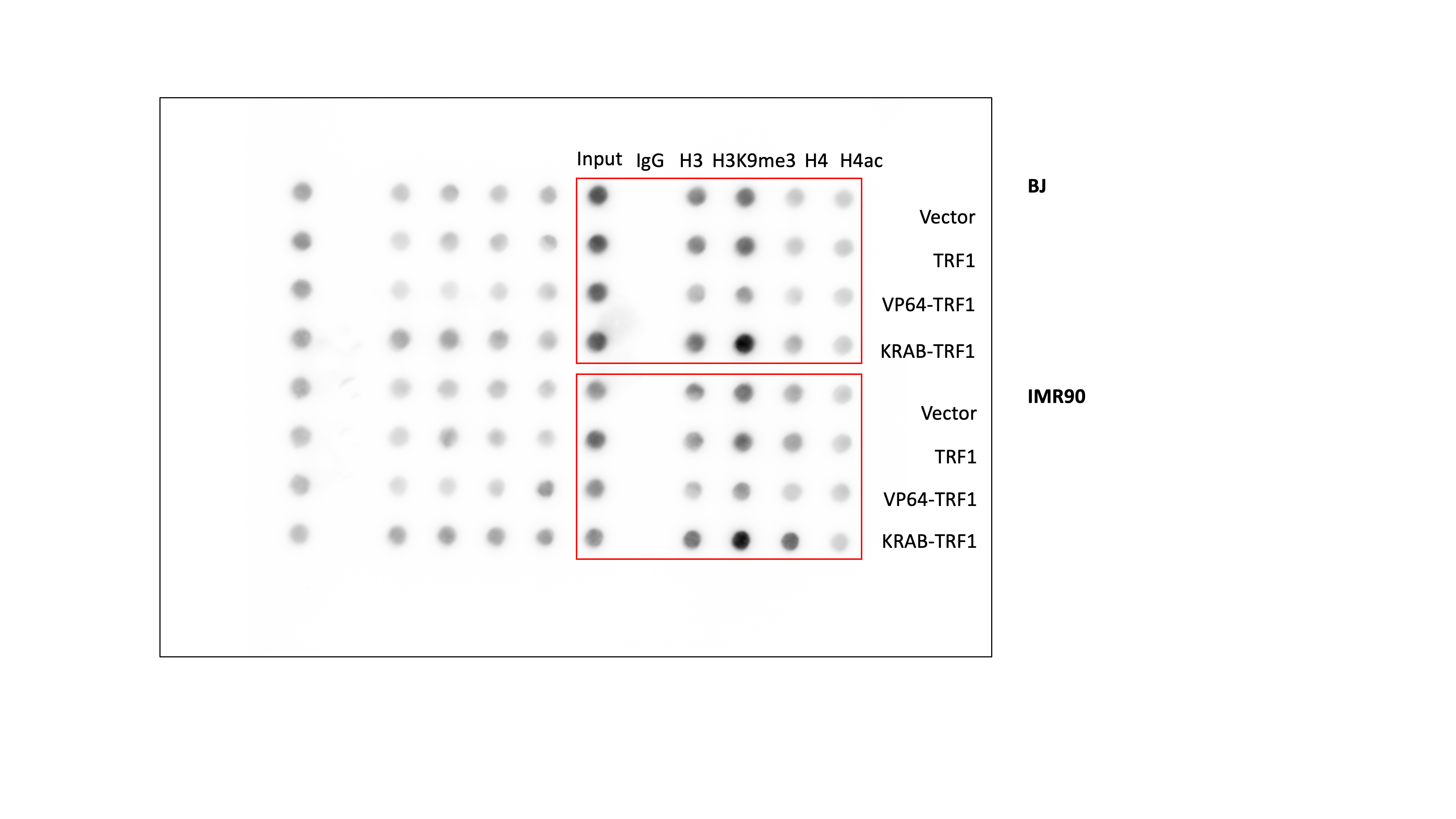

Supplement: Supplementary file 7 — Figure EV2 Source Data [file 44318_2026_760_MOESM7_ESM.zip › Figure EV2/C-E/Raw1_dotblot_annotated_Supplymentary.tiff]

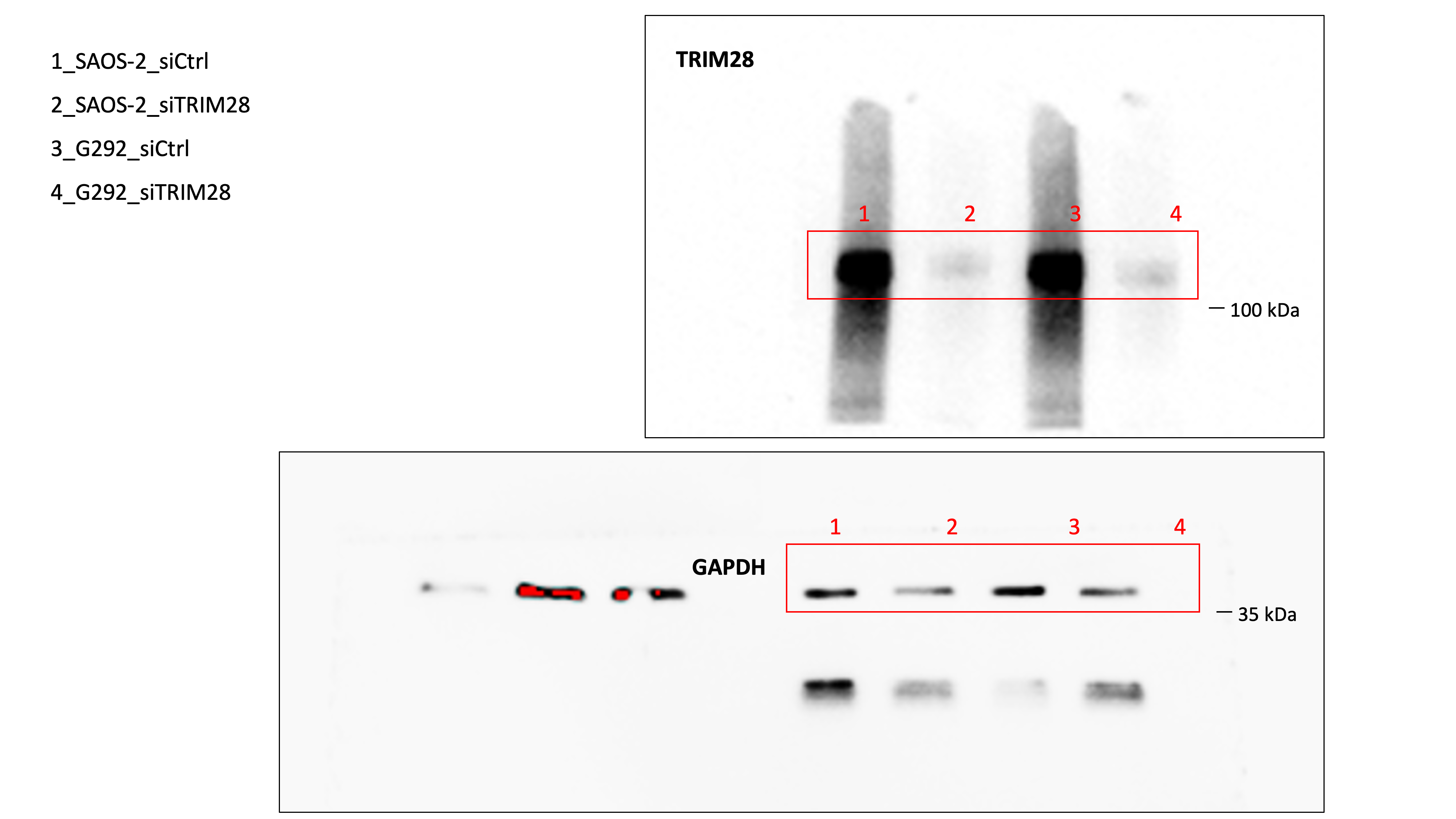

Supplement: Supplementary file 9 — Figure EV4 Source Data [file 44318_2026_760_MOESM9_ESM.zip › Figure EV4/A/RawBlot_annotated_Supplymentary.tiff]

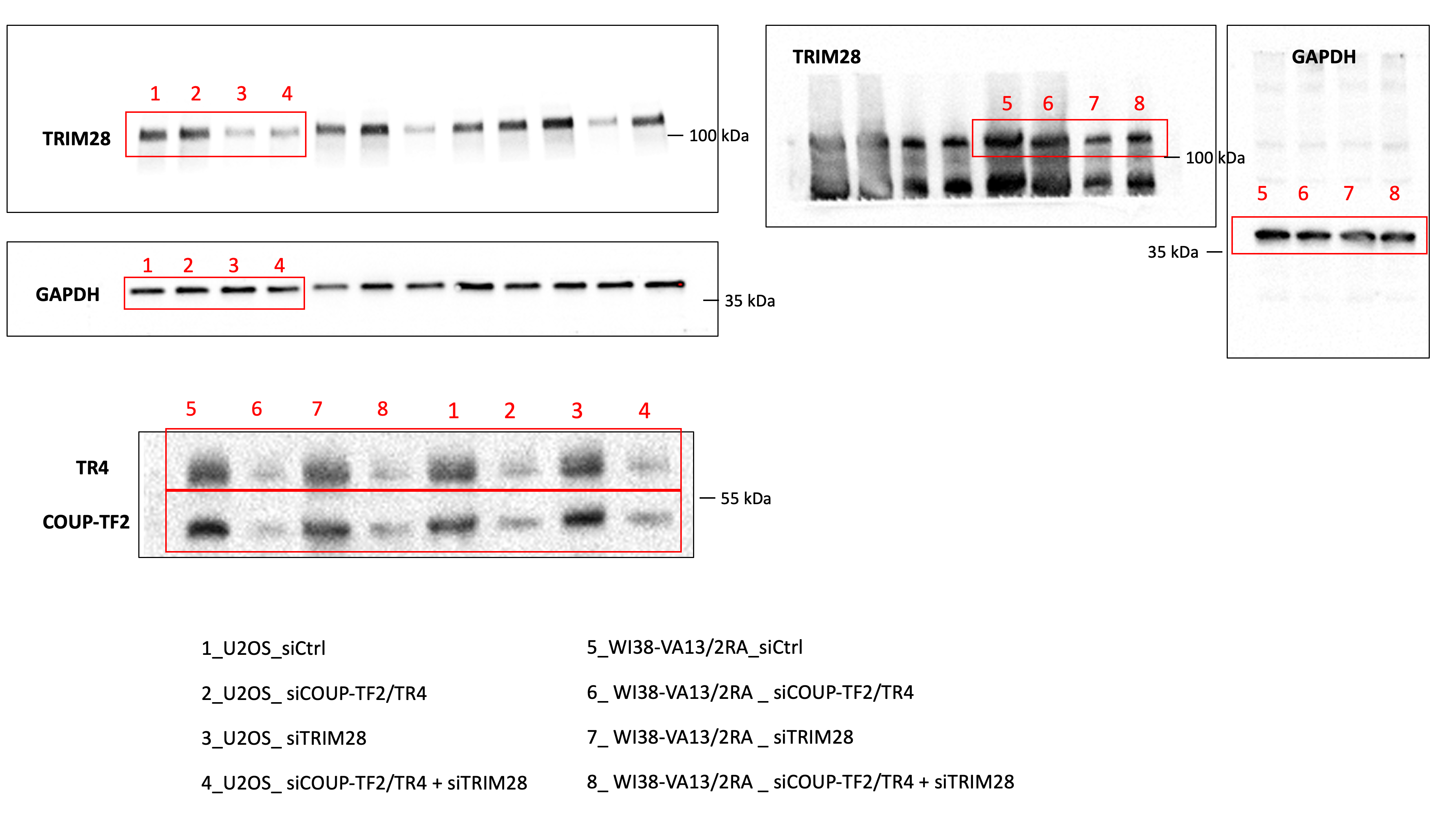

Supplement: Supplementary file 9 — Figure EV4 Source Data [file 44318_2026_760_MOESM9_ESM.zip › Figure EV4/D/RawBlot_annotated_Supplymentary.tiff]

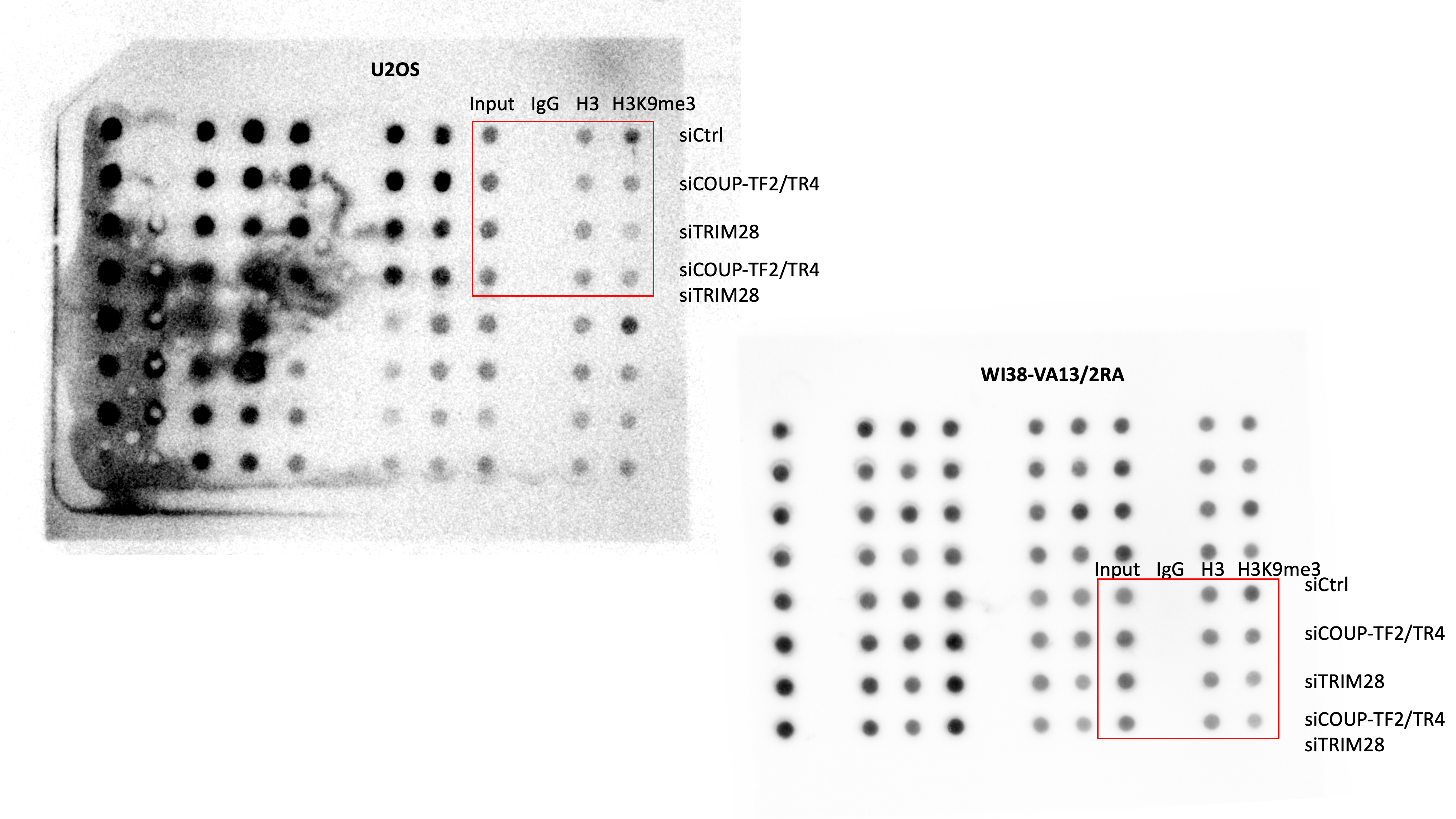

Supplement: Supplementary file 9 — Figure EV4 Source Data [file 44318_2026_760_MOESM9_ESM.zip › Figure EV4/E/Raw_dotblot_annotated_Supplymentary.tiff]

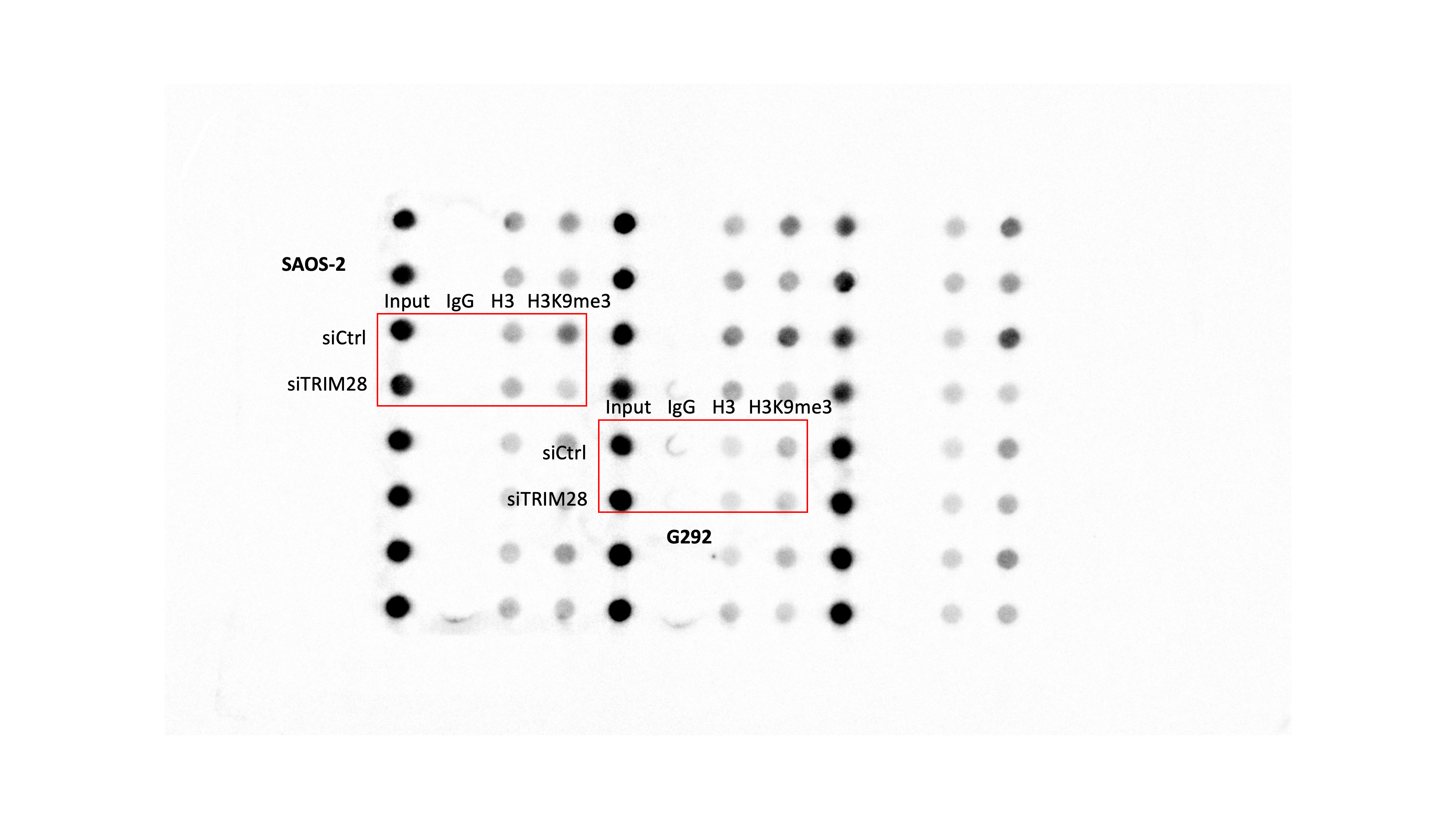

Supplement: Supplementary file 9 — Figure EV4 Source Data [file 44318_2026_760_MOESM9_ESM.zip › Figure EV4/B/Raw_dotblot_annotated_Supplymentary.tiff]

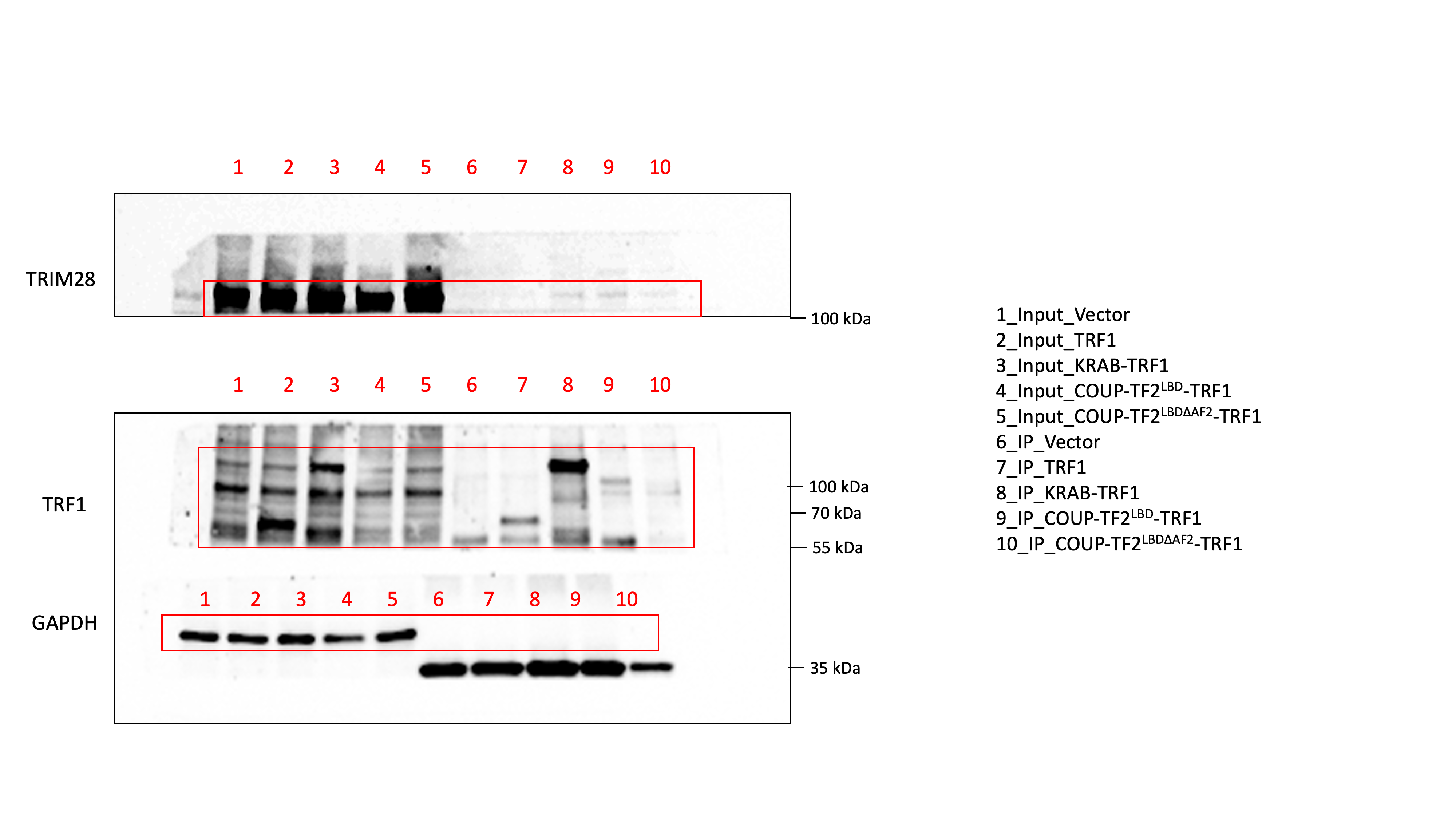

Supplement: Supplementary file 10 — Figure EV5 Source Data [file 44318_2026_760_MOESM10_ESM.zip › Figure EV5/G/Raw_blot_annotated_Supplymentary.tiff]

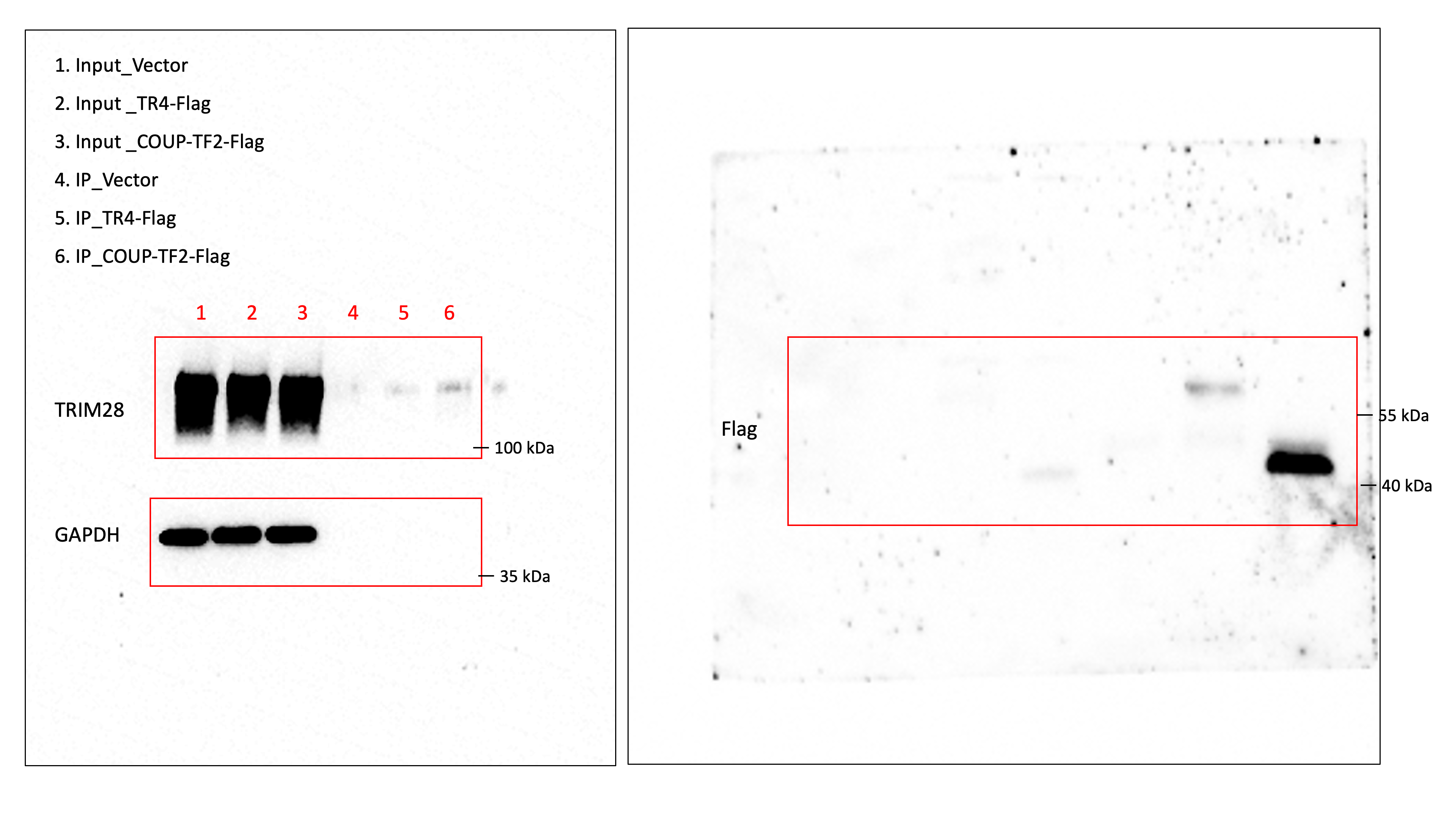

Supplement: Supplementary file 10 — Figure EV5 Source Data [file 44318_2026_760_MOESM10_ESM.zip › Figure EV5/F/Raw_blot_annotated_Supplymentary.tiff]

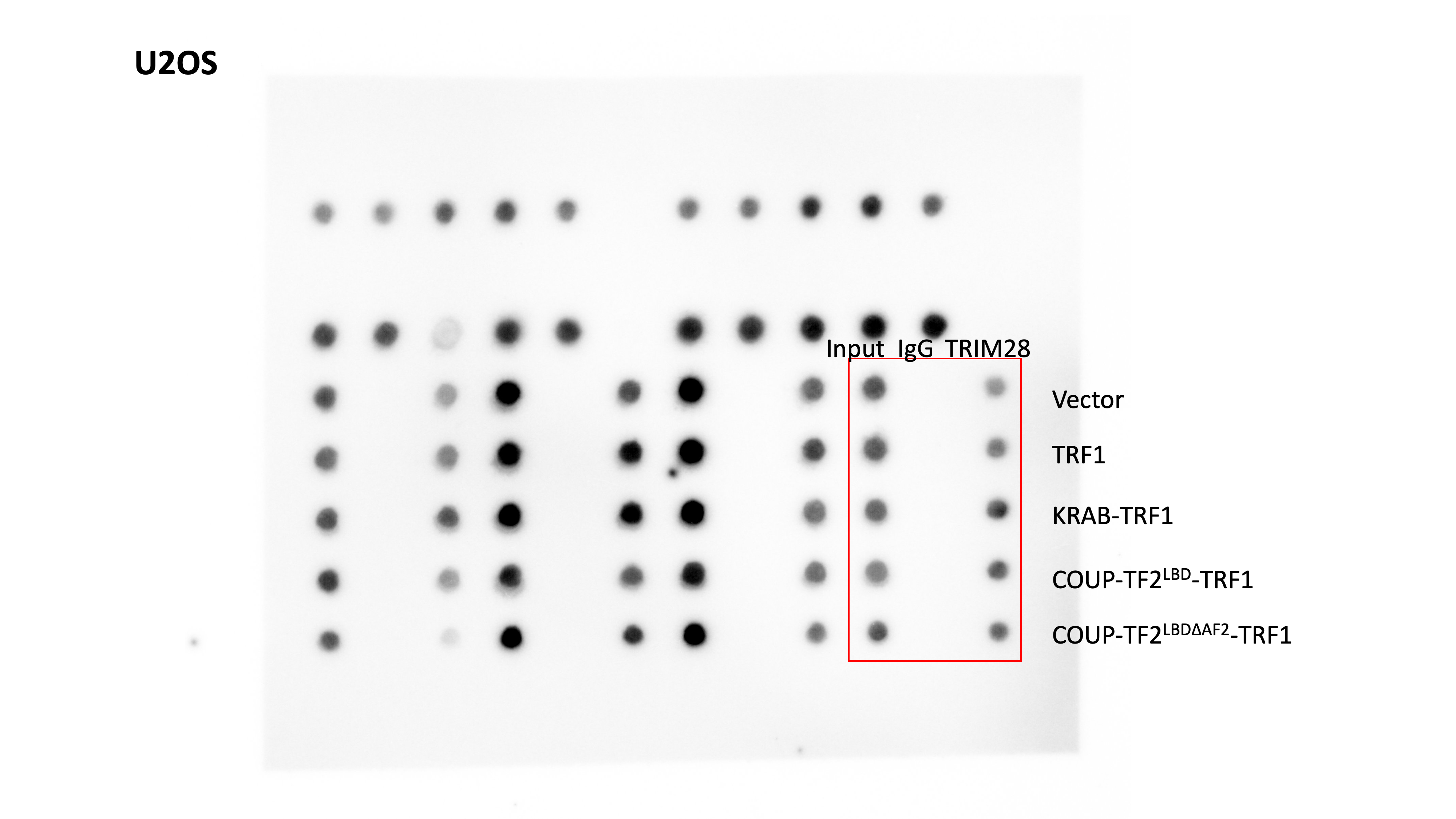

Supplement: Supplementary file 10 — Figure EV5 Source Data [file 44318_2026_760_MOESM10_ESM.zip › Figure EV5/A-D/Raw2_dotblot_annotated_Supplymentary.tiff]

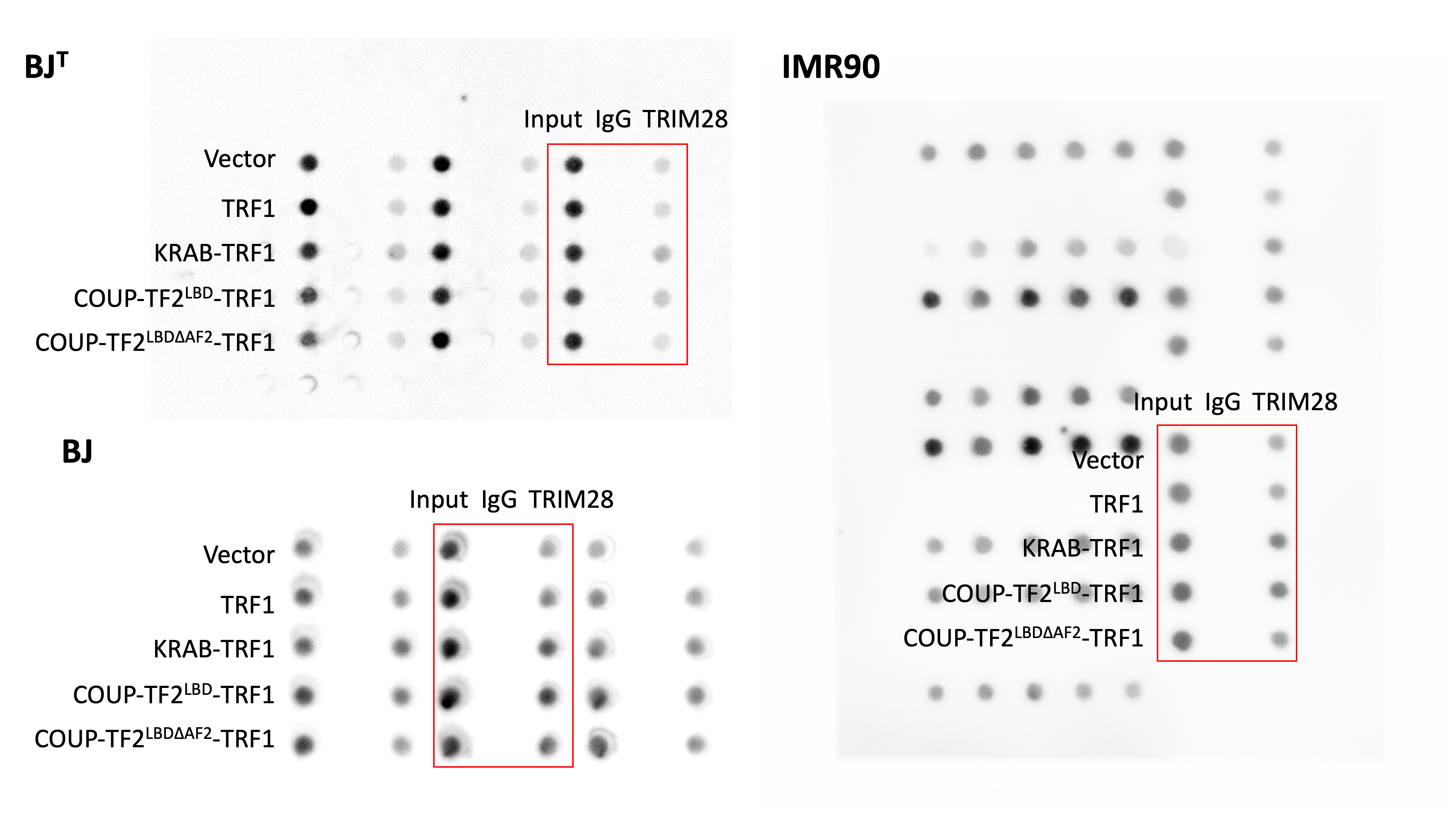

Supplement: Supplementary file 10 — Figure EV5 Source Data [file 44318_2026_760_MOESM10_ESM.zip › Figure EV5/A-D/Raw1_dotblot_annotated_Supplymentary.tiff]

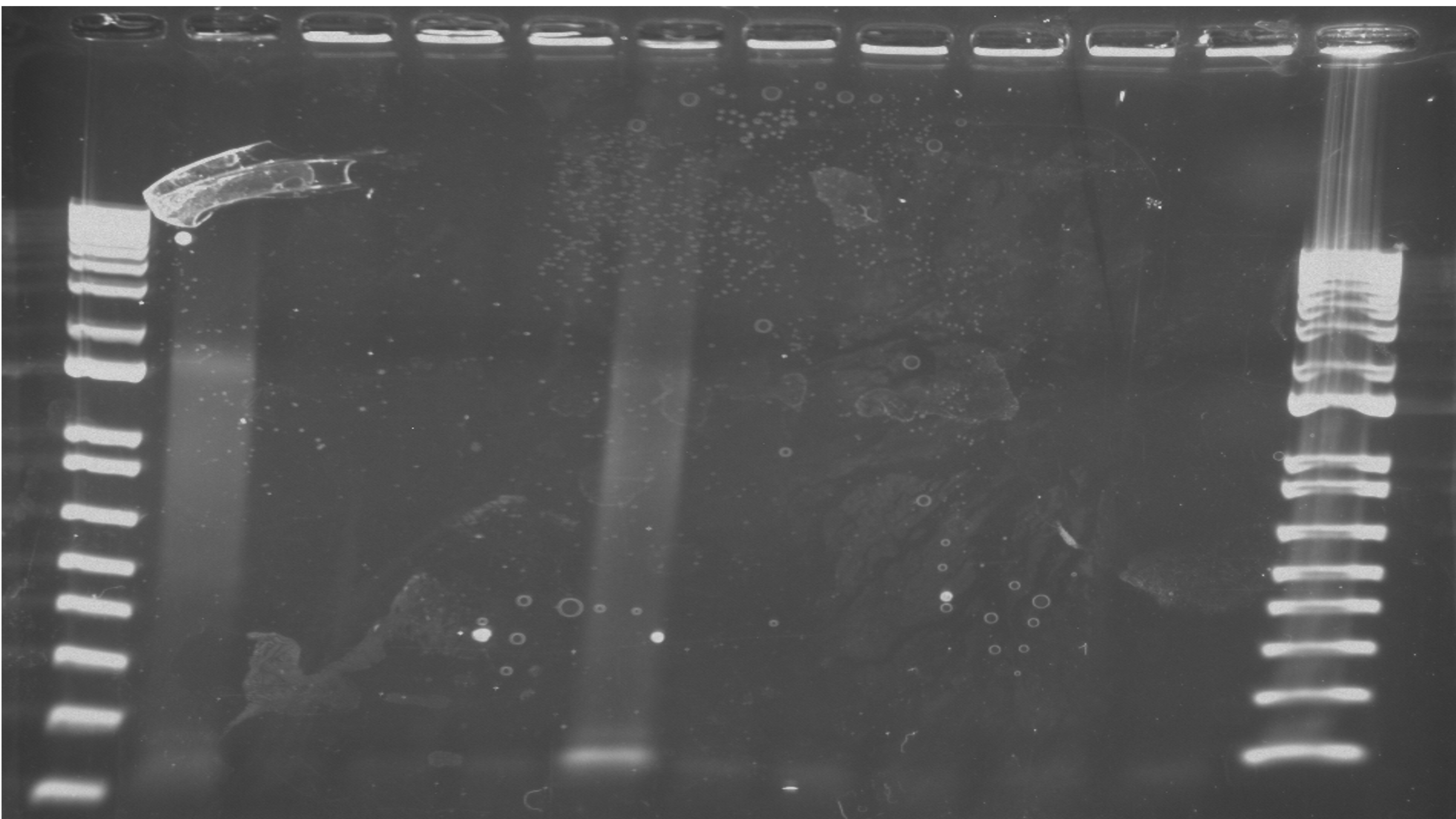

Supplement: Supplementary file 10 — Figure EV5 Source Data [file 44318_2026_760_MOESM10_ESM.zip › Figure EV5/E/Raw_Gel_Supplymentary.tiff]

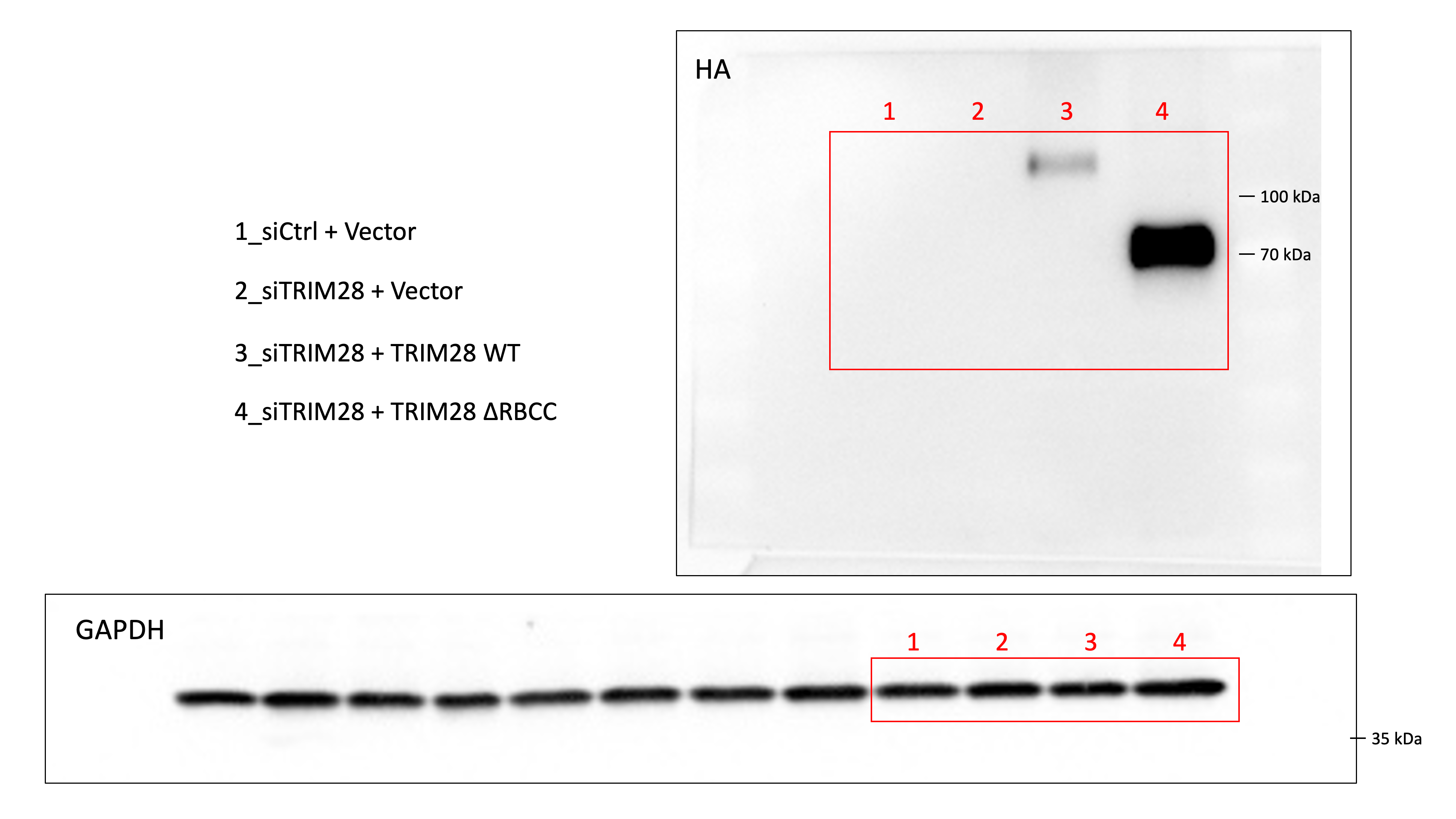

Supplement: Supplementary file 11 — Figure EFV6 Source Data [file 44318_2026_760_MOESM11_ESM.zip › FIgure EV6/A/RawBblot_Supplymentary.tiff]
