# Supplementary material for: Orphan nuclear receptors recruit TRIM28 to promote telomeric H3K9me3 for the ALT pathway
Source: EMBO J. 2026 Mar 31;45(10):3444–70. doi: 10.1038/s44318-026-00760-w (PMC13187458; doi:10.1038/s44318-026-00760-w)
Supplement: Supplementary file 12 — Expanded View Figures [file 44318_2026_760_MOESM12_ESM.pdf]

Expanded View Figures

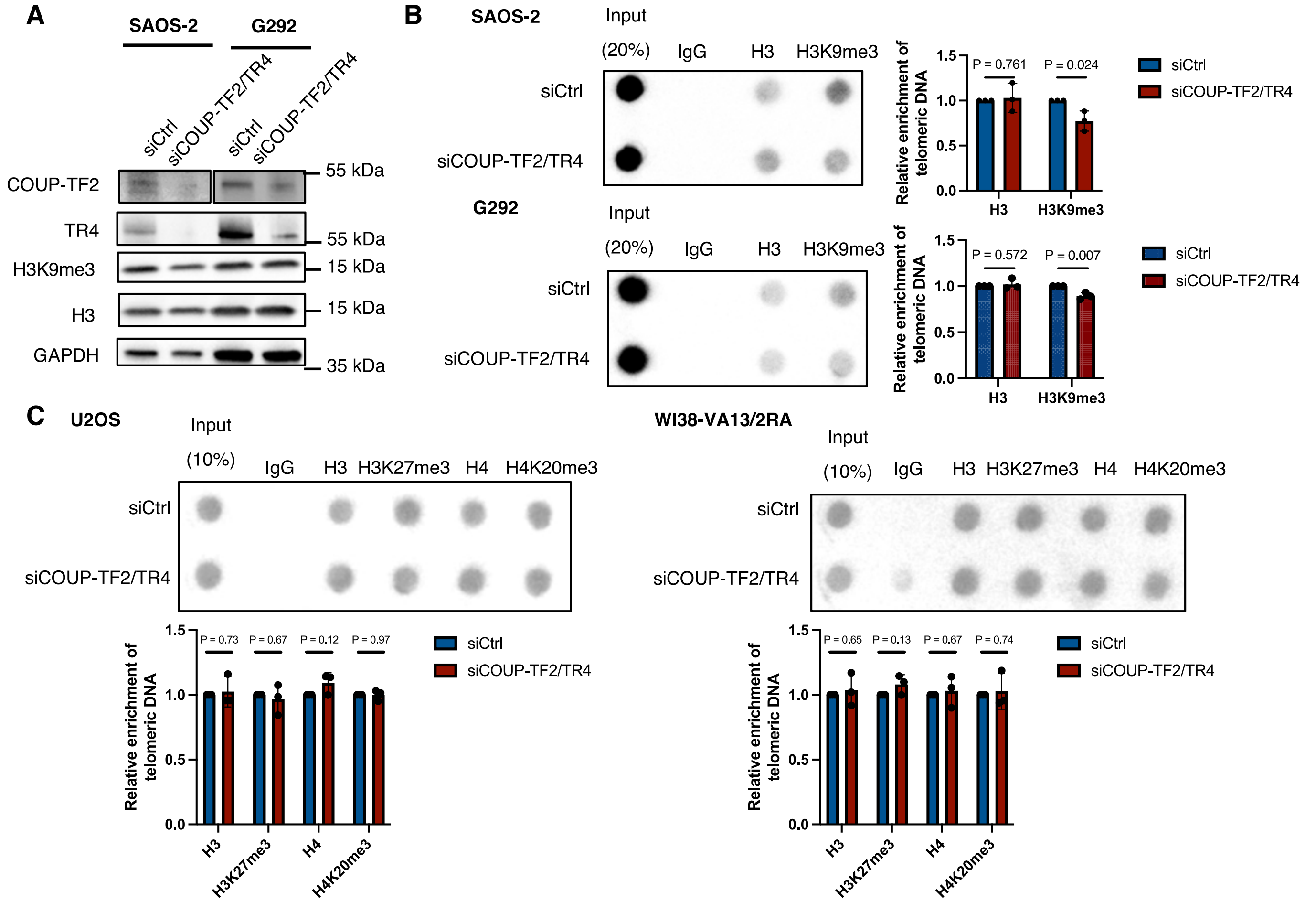

Figure EV1. Role of orphan nuclear receptors (NRs) in H3K9me3 activation at telomeres and characterization of COUP-TF2^{LBD}-TRF1 in promoting APB formation and ATDS.

(**A**) Western blot analysis showing the expression levels of COUP-TF2, TR4, H3, H3K9me3 and GAPDH in SAOS-2 and G292 cells following treatment for 6 days with specific siRNAs targeting these NRs. (**B**) Telomere-ChIP analysis of SAOS-2 and G292 cells 6 days after COUP-TF2 and TR4 knockdown to assess enrichment for telomeric DNA with the indicated histones and histone modifications (mean ± SD; $n = 3$ independent biological replicates). (**C**) Telomere-ChIP analysis of U2OS and WI38-VA13/2RA cells 6 days after COUP-TF2 and TR4 knockdown to assess enrichment for telomeric DNA with the indicated histones and histone modifications (mean ± SD; $n = 3$ independent biological replicates). (**B**, **C**) Statistical significance is noted as follows: ns$P > 0.05$, $^*P < 0.05$, as determined by the unpaired t test. Source data are available online for this figure.

 © The Author(s)

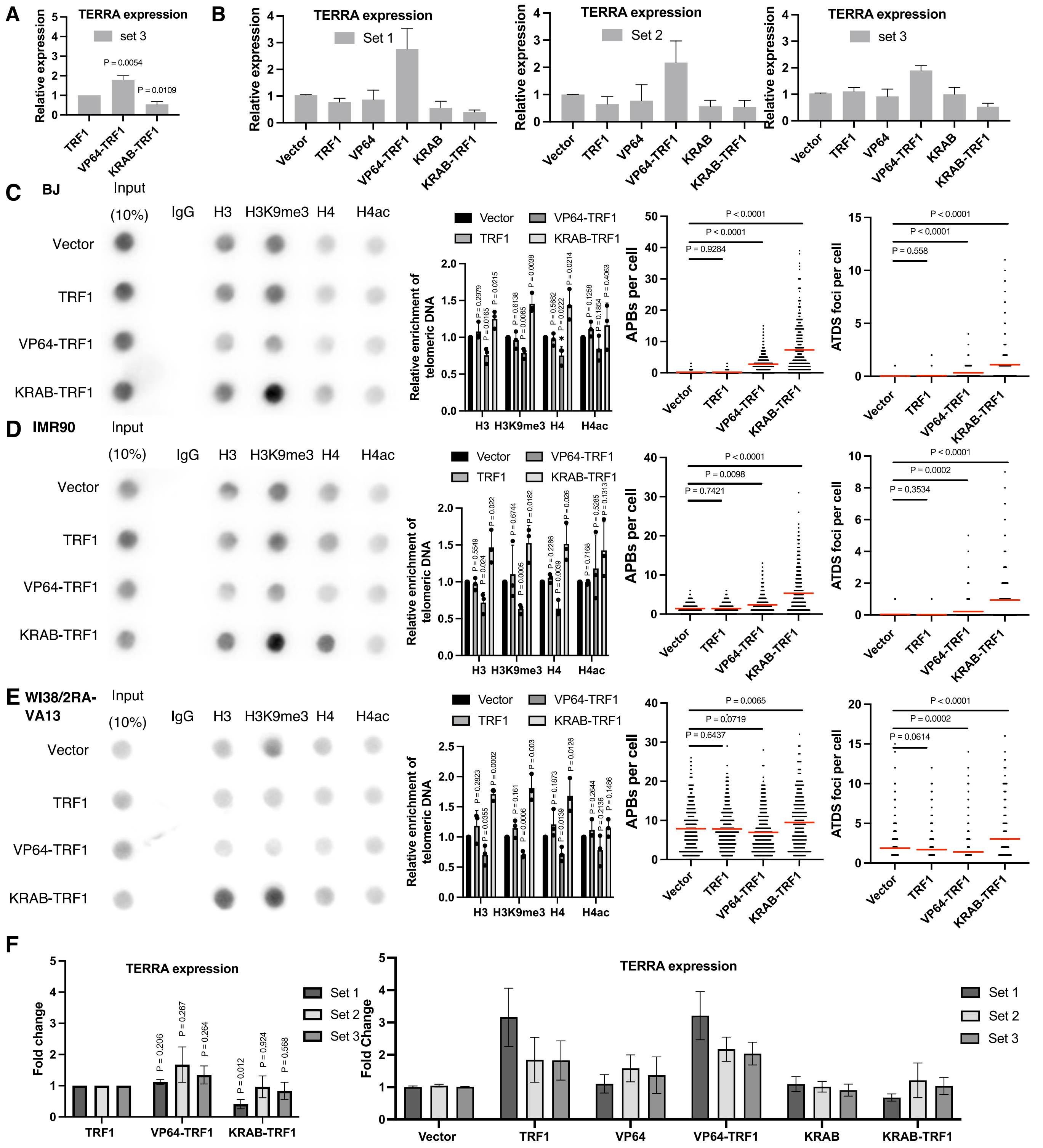
A
TERRA expression
set 3
Relative expression
P = 0.0054
P = 0.0109
TRF1
VP64-TRF1
KRAB-TRF1
B
TERRA expression
Set 1
Set 2
set 3
Relative expression
Vector
TRF1
VP64
VP64-TRF1
KRAB
KRAB-TRF1
C BJ
Input (10%)
IgG
H3
H3K9me3
H4
H4ac
Vector
TRF1
VP64-TRF1
KRAB-TRF1
Relative enrichment of telomeric DNA
APBs per cell
ATDS foci per cell
P < 0.0001
P = 0.9284
P = 0.558
D IMR90
P = 0.0098
P = 0.7421
P = 0.0002
P = 0.3534
E WI38/2RA-VA13
P = 0.0065
P = 0.0719
P = 0.6437
P = 0.0614
F
TERRA expression
Fold change
Fold Change
Set 1
Set 2
Set 3
P = 0.206
P = 0.267
P = 0.264
P = 0.012
P = 0.924
P = 0.568

Figure EV2. KRAB-TRF1 and VP64-TRF1 modulate telomeric chromatin and ALT-associated phenotypes in fibroblasts and ALT cells.

(**A**) Quantification of TERRA expression levels in VP64-TRF1 or KRAB-TRF1 BJ^T cells, normalized to those of TRF1 BJ^T cells, as determined by qPCR of RNA samples. TERRA transcripts from subtelomeric regions were quantified using primer set 3 targeting 5q, 7q, 9q, 10q, 13q, 16q, 20q, and 22q, with expression levels normalized to GAPDH. (**B**) Quantification of TERRA expression levels in KRAB-TRF1 or VP64-TRF1 BJ^T cells normalized to Flag BJ^T cells, as determined by qPCR of RNA samples. TERRA transcripts from subtelomeric regions were quantified using primer set 1 targeting Chr10q, primer set 2 targeting Chr15q, and primer set 3 targeting 5q, 7q, 9q, 10q, 13q, 16q, 20q, and 22q, with expression levels normalized to GAPDH. (**C–E**) Telomere-ChIP analysis in BJ, IMR90 and WI38-VA13/2RA cells expressing TRF1, KRAB-TRF1, or VP64-TRF1 reveals enrichment for telomeric DNA with various histones and histone modifications. Bar graphs present the quantification of telomeric DNA pulled down, normalized to vector-administered cells (mean ± SD; $n = 3$ independent biological replicates). First dot plot quantifies the number of APBs in individual BJ, IMR90, or WI38-VA13/2RA cells ($n > 150$). Left bar charts illustrate the percentages of APB-positive (APB +) cells, defined as those containing more than five APBs. Second dot plot quantifies the co-localization of telomeres with EdU in BJ^T cells ($n > 150$). Right bar charts display the percentage of ATDS-positive (ATDS +) cells containing more than three EdU+ Telomere foci. Red lines indicate the mean. (**F**) Quantification of TERRA expression in VP64-TRF1 or KRAB-TRF1 WI38-VA13/2RA cells relative to TRF1 WI38-VA13/2RA cells (left) or Flag WI38-VA13/2RA cells (right). TERRA transcripts were measured using primer set 1 targeting 10q, primer set 2 targeting 15q, and primer set 3 targeting 5q, 7q, 9q, 10q, 13q, 16q, 20q, and 22q. (**A**, **B**, **F**) (mean ± SEM; $n = 5$ biological independent experiments). (**A**, **F**) Statistical significance is noted as follows: ns$P > 0.05$, *$P < 0.05$, **$P < 0.01$, as determined by the unpaired *t* test. (**C–E**) Statistical significance is denoted as follows: ns$P > 0.05$, *$P < 0.05$, **$P < 0.01$, ***$P < 0.001$, ****$P < 0.0001$, as determined by Mann–Whitney *U* test. Source data are available online for this figure.

 © The Author(s)

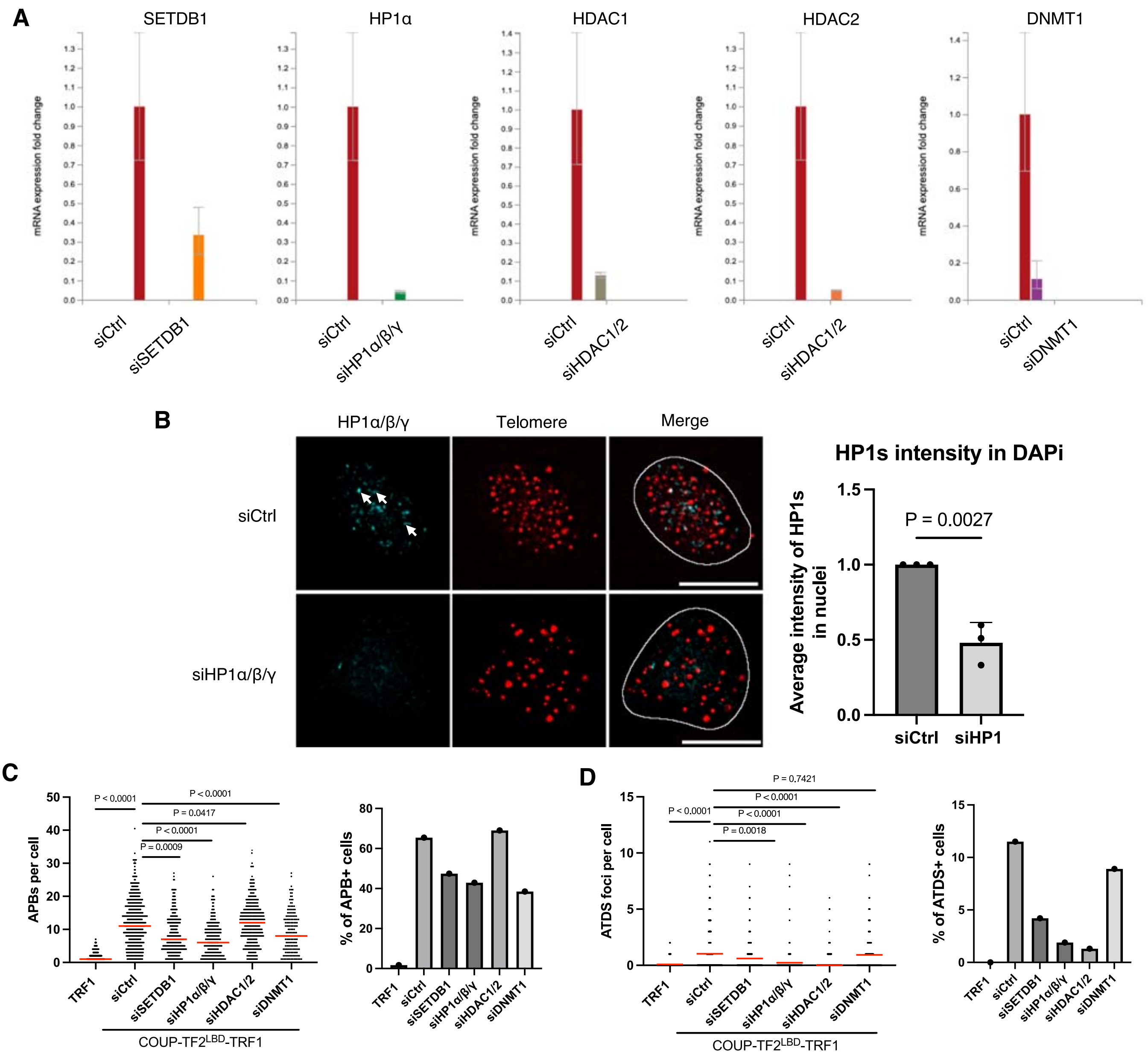

Figure EV3. Functional analysis of siRNA-mediated knockdown of heterochromatin-associated proteins in BJ^{T} cells expressing COUP-TF2^{LBD}-TRF1.

(**A**) qPCR and (**B**) IF data showing the gene silencing efficiency of siRNA sequences targeting specific RNAs in BJ^{T} cells expressing COUP-TF2^{LBD}-TRF1. For qPCR, data represent the mean of three technical replicates (mean ± SEM; $n = 3$). For IF, the bar chart indicates the average HP1 signal intensity per nucleus (mean ± SEM; $n = 3$ independent biological replicates). White outlines indicate DAPI segmentation and scale bar, 10 μm. White arrows indicate foci localized at telomeres. (**C**) Dot plots to quantify the number of APBs in TRF1 or COUP-TF2^{LBD}-TRF1 BJ^{T} cells treated with specific siRNAs targeting heterochromatin-associated proteins ($n > 150$ cells). (**D**) Dot plots showing quantification of EdU+ APB foci in TRF1 or COUP-TF2^{LBD}-TRF1 BJ^{T} cells treated with specific siRNAs targeting heterochromatin-associated proteins ($n > 150$ cells). Red lines in the dot plots indicate the mean. Statistical significance is denoted as follows: ns$P > 0.05$, **$P < 0.01$, ****$P < 0.0001$, as determined by Mann–Whitney *U* test. All knockdown experiments involved treating cells with specific siRNAs for 6 days. Source data are available online for this figure.

© The Author(s)

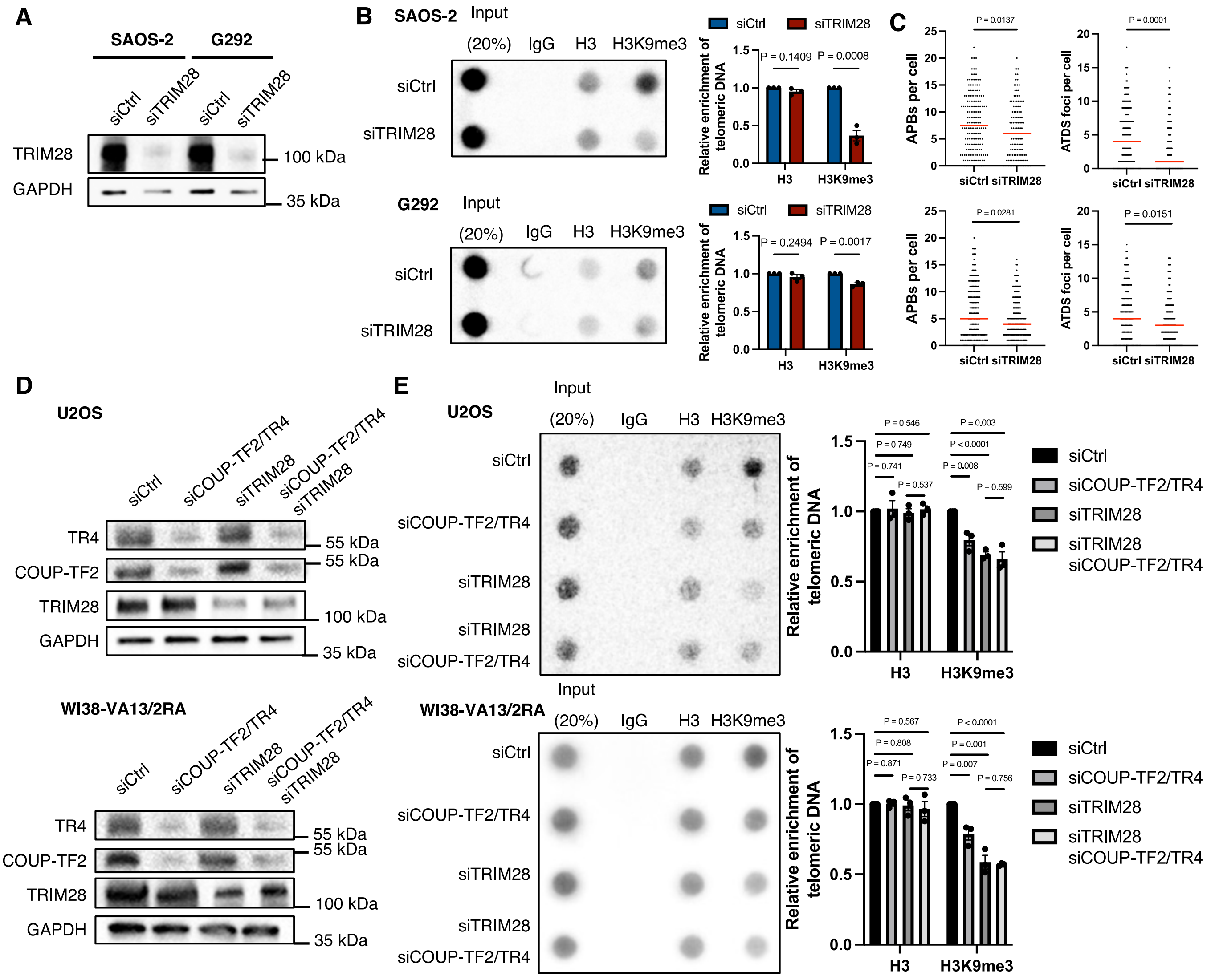

Figure EV4. TRIM28 depletion reduces telomeric H3K9me3, APBs, and ATDS in ALT cells and shows an epistatic effect with orphan NRs.

(**A**) Western blot analysis of TRIM28 expression in SAOS-2 and G292 cells after 6 days of treatment with TRIM28-targeting siRNAs. (**B**) Telomere-ChIP analysis of U2OS and WI38-VA13/2RA cells 6 days following TRIM28 knockdown to assess enrichment for telomeric DNA with specific histones and histone modifications. Bar graphs show quantitation of the telomeric DNA pulled down, normalized to input and control cells (mean ± SD; $n = 3$ independent biological replicates). (**C**) Dot plots show the quantification of APBs or EdU+ Telomere (ATDS) foci in individual SAOS-2 and G292 cells ($n > 150$). (**D**) Western blot analysis showing the expression levels of COUP-TF2, TR4, TRIM28 and GAPDH in U2OS and WI38-VA13/2RA cells following treatment for 6 days with specific siRNAs targeting these NRs, TRIM28 or both. (**E**) Telomere-ChIP analysis of U2OS and WI38-VA13/2RA cells 6 days after COUP-TF2/TR4, TRIM28 or both knockdown to assess enrichment for telomeric DNA with the indicated histones and histone modifications (mean ± SD; $n = 3$ independent biological replicates). (**B**, **E**) Statistical significance is noted as follows: ns$P > 0.05$, *$P < 0.05$, as determined by the unpaired *t* test. (**C**) Statistical significance is denoted as follows: ns$P > 0.05$, *$P < 0.05$, **$P < 0.01$, ***$P < 0.001$, ****$P < 0.0001$, as determined by Mann–Whitney *U* test. Source data are available online for this figure.

 © The Author(s)

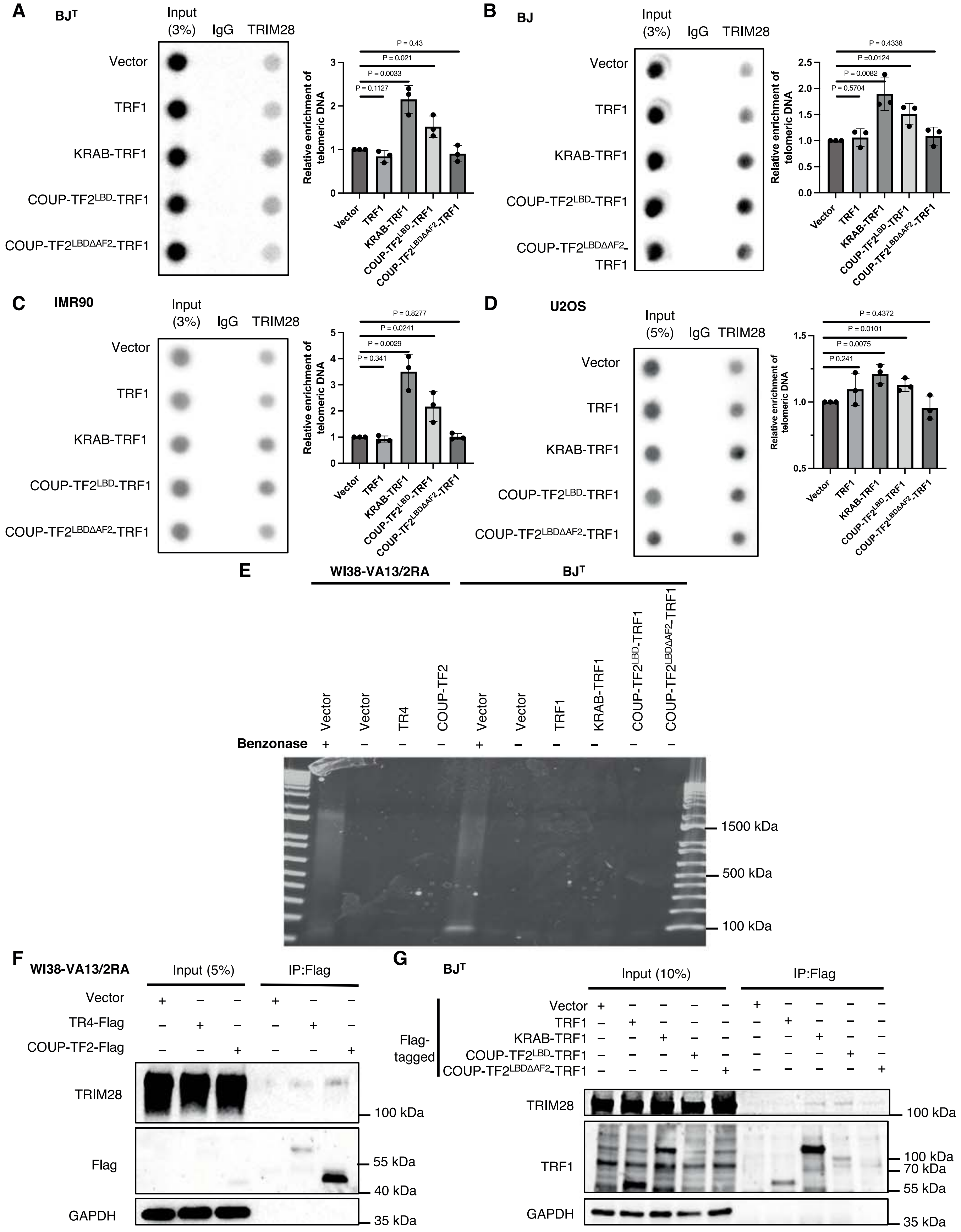

A
BJT
Input (3%)
IgG
TRIM28
Vector
TRF1
KRAB-TRF1
COUP-TF2LBD-TRF1
COUP-TF2LBDΔAF2-TRF1
Relative enrichment of telomeric DNA
P = 0.43
P = 0.021
P = 0.0033
P = 0.1127
B
BJ
Input (3%)
IgG
TRIM28
P = 0.4338
P =0.0124
P = 0.0082
P = 0.5704
C
IMR90
Input (3%)
IgG
TRIM28
P = 0.8277
P = 0.0241
P = 0.0029
P = 0.341
D
U2OS
Input (5%)
IgG
TRIM28
P = 0.4372
P = 0.0101
P = 0.0075
P 0.241
E
WI38-VA13/2RA
BJT
Benzonase
Vector
TR4
COUP-TF2
1500 kDa
500 kDa
100 kDa
F
WI38-VA13/2RA
Input (5%)
IP:Flag
Vector
TR4-Flag
COUP-TF2-Flag
TRIM28
Flag
GAPDH
100 kDa
55 kDa
40 kDa
35 kDa
G
BJT
Input (10%)
IP:Flag
Flag-tagged
TRIM28
TRF1
GAPDH
100 kDa
70 kDa
55 kDa
35 kDa

Figure EV5. COUP-TF2/TR4-dependent recruitment of TRIM28 to telomeres through DNA-independent interaction.

(**A–D**) Telomere-ChIP analysis of BJ^T, BJ, IMR90 and U2OS cells stably expressing fusion proteins to quantify enrichment of TRIM28-associated telomeric DNA. The bar graphs show quantitation of the telomeric DNA pulled down, normalized to control cells (mean ± SD; $n = 3$ independent biological replicates). (**E**) Agarose gel analysis of cell lysates from WI38-VA13/2RA and BJ^T cells with or without Benzonase treatment, showing effective DNA removal upon Benzonase digestion, as evidenced by the reduced nucleic acid signal. (**F**) Co-immunoprecipitation (IP) of TRIM28 with COUP-TF2/TR4 in WI38-VA13/2RA cells. Lysates (2×10^7 cells) were treated with Benzonase to degrade DNA, then subjected to IP using TRIM28 or Flag antibodies and analyzed by western blot, demonstrating that the interaction is DNA-independent. (**G**) Co-immunoprecipitation of TRIM28 with TRF1-based fusion proteins in BJ^T cells. Lysates were treated with Benzonase, followed by IP with TRIM28 or TRF1 antibodies and Western blot analysis, confirming that TRIM28 interacts with COUP-$TF2^{LBD}$-TRF1 and KRAB-TRF1 in a DNA-independent manner. (**A–D**) Statistical significance is noted as follows: ns$P > 0.05$, *$P < 0.05$, as determined by the unpaired *t* test. Source data are available online for this figure.

 © The Author(s)

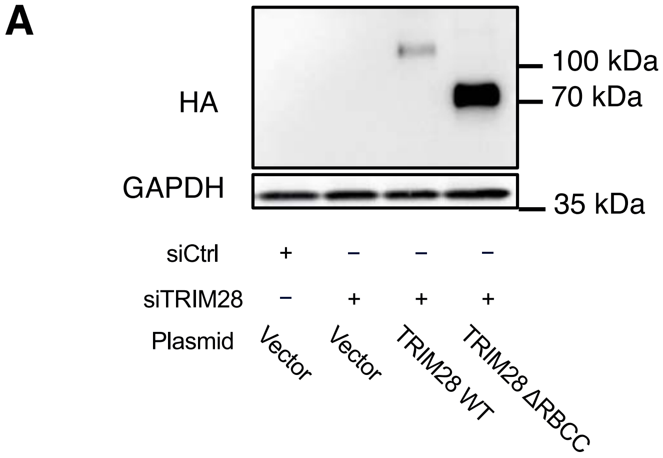

Figure EV6. Western blot analysis of mutant TRIM28 expression in WI38-VA13/2RA cells.

(**A**) Western blot analysis of WI38-VA13/2RA cells after 6 days of TRIM28 knockdown and 2 days of WT TRIM28 or mutant TRIM28 expression, confirming expression of the constructs using an anti-HA antibody. Source data are available online for this figure.

© The Author(s)
